# Urbanization and food consumption in India

**DOI:** 10.1038/s41598-020-73313-8

**Published:** 2020-10-14

**Authors:** Bhartendu Pandey, Meredith Reba, P. K. Joshi, Karen C. Seto

**Affiliations:** 1 Yale School of the Environment, Yale University, New Haven, CT 06511 USA; 2 School of Environmental Sciences, Jawaharlal Nehru University, New Mehrauli Road, Delhi, New Delhi 110067 India; 3 Special Centre for Disaster Research, Jawaharlal Nehru University, New Mehrauli Road, Delhi, New Delhi 110067 India

**Keywords:** Sustainability, Socioeconomic scenarios, Environmental impact

## Abstract

The shift towards urban living is changing food demand. Past studies on India show significant urban–rural differences in food consumption. However, a scientific understanding of the underlying relationships between urbanization and food consumption is limited. This study provides the first detailed analysis of how urbanization influences both quantity and diversity of food consumption in India by harnessing the strength of multiple datasets, including consumer expenditure surveys, satellite imagery, and census data. Our statistical analysis shows three main findings. First, in contrast to existing studies, we find that much of the variation in food consumption quantity is due to income and not urbanization. After controlling for income and state-level differences, our results show that average consumption is higher in urban than rural areas for fewer than 10% of all commodities. That is, there is nearly no difference in average consumption between urban and rural residents. Second, we find the influence of urbanization as a population share on food consumption diversity to be statistically insignificant (*p-value* > 0.1). Instead, the results show that infrastructure, market access, percentage working women in urban areas, and norms and institutions have a statistically significant influence. Third, all covariates of food consumption diversity we tested were found to be associated with urbanization. This suggests that urbanization influences on food consumption are both indirect and multidimensional. These results show that increases in the urban population size alone do not explain changes in food consumption in India. If we are to understand how food consumption may change in the future due to urbanization, the study points to the need for a more complex and multidimensional understanding of the urbanization process that goes beyond demographic shifts.

## Introduction

Over the next three decades, 2.3 billion more people will be living in urban areas worldwide^1^. High consumption levels of animal-based products, refined animal fat, edible oil, refined sugar, and alcohol characterize diets in urbanized societies with higher economic development^2^. Studies show that urbanizing countries are rapidly converging to these diets, increasing human health risks related to conditions such as obesity and hypertension, and non-communicable diseases such as diabetes, heart disease, and stroke^3, 4^. These dietary changes also raise concerns such as greater use of land, water, and energy resources^5^, greenhouse gas emissions^6^, inequitable access to healthy food^7^, and food security^8^. Based on these trends and linkages, impending urbanization and associated dietary changes pose significant human health and environmental sustainability challenges. To fully comprehend these implications, understanding how urbanization influences food consumption is essential. However, the pathways that undergird urbanization influences on food consumption and diets are less clear^9^.

Previous related studies considered urbanization as a demographic process (an increase in the urban share of the total population) and omitted the spatial, economic, social, and cultural changes that are concomitant with urbanization^10^. For example, Engel’s and Bennett’s laws assert that with rising incomes, the total share of spending on food decreases while diets shift from starchy staples to a more diversified consumption of meats, dairy, oils, fruits, and vegetables^11,12^. If urbanization drives income growth^13^, it follows that urbanization can lead to changes in food consumption mediated by increases in income. However, most existing studies do not recognize income as an urbanization influence and sometimes confound the two^14,15^. Studies have also reported unique urban effects—beyond income—on food consumption. These effects can be associated with increased food availability^16^, the opportunity cost of women’s time^17,18^, access to cooking and availability of cold storage facilities^19^, and exposure-mediated changes in taste and preferences^20^. These factors imply that urbanization influences on food consumption are complex and multidimensional, yet other factors are not well understood and frequently considered in isolation rather than collectively comprising the urbanization process.

The UN estimates that 90% of future urban population growth will take place in Asia and Africa, with China, India, and Nigeria accounting for one-third of the growth between 2018 and 2050. Diets and patterns of urbanization are likely different for countries at different phases of urbanization^21^. The present study focuses on India, a country in the early stages of an urban demographic transition and economic development. India serves as an important case study for how diets may change in an urbanizing society. This study investigates five questions : (1) Does urbanization (living in urban areas and as a demographic share) affect the quantity and diversity of food consumed by households? (2) How does the quantity and diversity of food consumed vary between large urban, small urban, and rural areas? (3) Are the observed variations in the quantity and diversity of food consumed due to income or urbanization or both? (4) How do urbanization dimensions such as infrastructure, market access, women’s participation in the workforce, and norms and institutions associate with food consumption in India? (5) How do factors associated with food consumption relate to living in urban areas and as a demographic share? These questions are aimed to advance our understanding of urbanization influences on food consumption. New knowledge contributions include: evaluating whether urban–rural differences remain significant after controlling for income and other covariates, assessing whether consumption varies by urban size, distinguishing between urban metropolitan and urban non-metropolitan areas, and comparing how urbanization influences differ between quantity and diversity of food consumed. One important innovation of this study is that it explicitly examines the significance of multiple dimensions of urbanization and not only the demographic component.

## Study area and data

### Study area

A little over one-third of India’s population currently lives in urban areas. Demographic projections suggest that India will be 50% urbanized by 2050. Much of this transition will concentrate in medium- and small-sized cities—with less than one million residents—that are also growing the fastest in India^22^. National food consumption statistics suggest that this impending urban transition can lead to large-scale dietary changes^23^ (Fig. S1). These statistics highlight that the average quantities of food consumed in urban areas are generally higher than in rural areas. Furthermore, the per-capita consumption of cereals, pulses, and sugar is declining in both urban and rural areas, whereas per capita consumption of other food commodities—such as animal products, oils, and fruits and vegetables—is increasing. However, changes in per-capita consumption are faster in rural areas than in urban areas for most food groups except sugar and spices. These patterns and trends provide preliminary evidence for urbanization influences on food consumption.

This study provides, to the best of our knowledge, the first detailed analysis of urbanization influences on the quantities of food consumed (across 124 commodities) and food consumption diversity in India. Past research has studied urbanization-food consumption linkages using two different measures: non-expenditure and expenditure-based measures. Non-expenditure based measures focused on quantities consumed or caloric intake, whereas expenditure-based measures focused on commodity-wise expenditures or expenditure shares on food^24^. Urbanization influences can vary across these different measures. The present study focuses on both the quantity and diversity of food consumed for five main reasons. First, national statistics show apparent urban–rural differences in the quantities of food consumed. Second, food consumption quantities link potential human health and environmental implications more directly than expenditure-based measures. Third, while data on food quantities can be used to calculate calorie intake, this conversion necessitates an assumption that for a given food item, the relationship between quantity and calorie content is constant. Fourth, following the same reasoning, a calorie intake-based measure of diversity can also be problematic. Finally, we expect that households living in urban areas tend to consume more and diversify their consumption due to increased food availability and accessibility, compared to rural areas.

This study examines variations in food consumption at the household, district, and state levels. The administrative hierarchy in India follows the following order: States/Union Territories (UTs), Districts, Sub-districts, and Towns (urban areas) and Villages (rural areas). As per the 2011 census, there were 28 states and 8 UTs, 641 districts, and 6,075 sub-districts (Fig. S2). Urban areas comprise two types of administrative units in India: Statutory towns and Census towns. Statutory towns are defined by statute, whereas Census towns are identified based on three criteria: a minimum population (5000), a minimum percentage of the male working population engaged in any non-agricultural activity (75%), and a minimum population density of 400 persons/km^2^. Rural areas are administrative areas not identified as urban and comprised of villages.

### Data

Since one of our primary aims is to examine multiple dimensions of urbanization, we use five datasets that provide different lenses on urban India and food consumption: (1) expenditure survey data for household food consumption^23^, (2) census data for demographic information^25^, (3) Global Human Settlements Layer (GHSL) (v1.0) for urban built-up area^26^, (4) DMSP/OLS nighttime lights (NTLs) for built-up infrastructure^27^, and (5) global accessibility data for travel time^28^ (Supplementary Text S1). The consumer expenditure survey collected by India’s National Sample Survey Office (NSSO) yields the quantity and diversity of food consumed at the household level^23^. This survey is nationally representative, with a sample size of 101,662 households spread across urban and rural areas of all states and UTs. Location information in the survey can be used to geocode all households at the district-level. By manually creating a lookup table linking state and district information in the survey and the census data, we classified all households into three groups: urban metropolitan (UM), urban non-metropolitan (UNM), and rural (R) households. All urban households located in districts with urban areas of population size greater than 1,000,000—recognized by the census of India as “*major urban centers*”^25^—were labeled as UM and the rest of the urban households as UNM. We labeled all of the remaining households as R. Census data also provides two variables that we used in this study: level of urbanization (share of urban population to total population) and % working women (share of the female population working in urban areas to total population).

We developed a proxy measure of infrastructure based on the GHSL-derived built-up area and DMSP/OLS NTLs. We aggregated built-up area estimates (sum of built-up area) and NTLs (average NTLs intensity) at the district-level from GHSL (~ 38 m spatial resolution) for the year 2014 and NTLs (~ 1 km spatial resolution) for the year 2011, respectively. Given the difference of three years between the two datasets, the analysis assumes negligible changes in district-level variations in the built-up area between 2011 and 2014. We calculated district-level infrastructure stock by multiplying aggregated built-up area and average NTLs. This measurement approach does not require the colocation of NTLs and built-up area at the pixel level, even though NTLs and built areas may colocate in certain areas. Furthermore, it leverages the two datasets in a complementary manner: GHSL measures impervious surface and estimates built-up area, and NTLs measure outdoor lighting. Finally, we calculated average district-level travel time to the nearest city of population size at least 50,000 (in 2000) using a global accessibility raster at ~ 1 km spatial resolution. This variable is used as a proxy for market access and assumes a negligible change in district-level accessibility variations over the 2000–2011 period. Lastly, we distinguished states with high food consumption diversity (µ_food diversity_ = 1.1) from low food diversity (µ_food diversity_ = 0.90) (Fig. S2). This variable inadvertently captures differences in norms and institutions related to food consumption.

## Methodology

The conventional approach observes the effect of increasing urban population share or the impact of living in urban areas or both on food consumption. There are at least two limitations to this approach. First, urban–rural comparisons assume that the urban–rural classifications are unbiased and that the dichotomy sufficiently captures fundamental differences between urban and rural living. This supposition is problematic as biases may exist in the classification process^29,30^ but also because of significant heterogeneities in the structure and functioning of urban areas^31^. Second, urban–rural differences in food consumption can be due to multiple factors and do not isolate the impacts of individual dimension^32^. These limitations also apply to approaches focusing on the urban share of the total population^33^. Still, previous studies focusing on India have chiefly reported urban–rural differences to emphasize urbanization influences, at the national^34–36^ and regional scales^37,38^. Findings from these studies broadly corroborate with national statistics and suggest that urbanization positively links with food consumption diversity in India.

**Figure 1:**
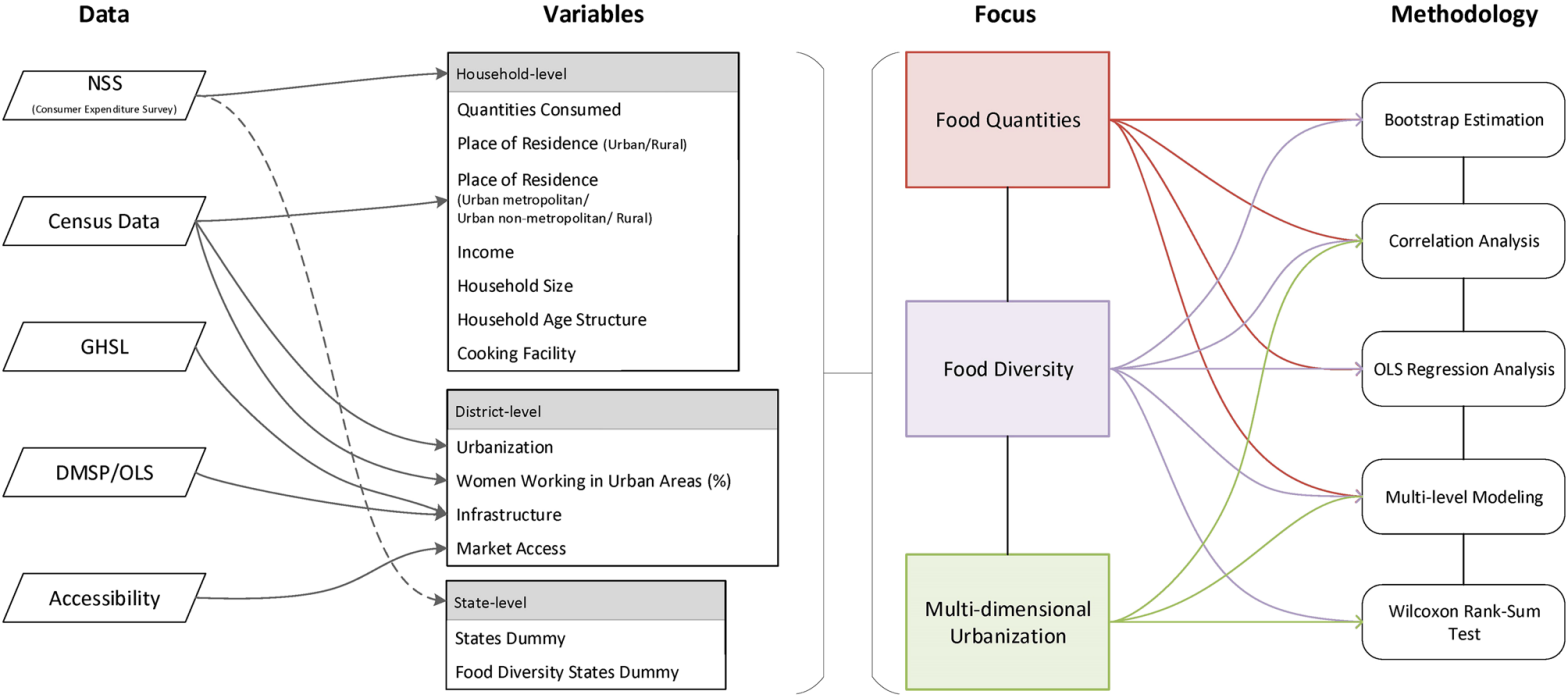
Flowchart outlining the data and methodology used in the study.

This study also offers some methodological advancements. In addition to examining average consumption differences between households residing in urban and areas, the present study quantifies differences in average food consumption between UM, UNM, and R areas. This supplementary analysis assesses whether the impact of living in urban areas differs between UM and UNM areas. It also evaluates whether the impact of living in urban areas holds after controlling for income and state-level differences, as these can also lead to urban–rural differences. Furthermore, it uses multi-source and multi-scale datasets, including satellite remote sensing-derived and census data products, to more comprehensively examine urbanization influences, i.e., by examining aspects of urbanization, such as women working in urban areas, infrastructure, and market access. Lastly, it employs five statistical approaches towards a thorough investigation of urbanization influences: analyzing urban–rural differences using bootstrap estimation, correlation analysis, ordinary least squares (OLS) regression modeling, multilevel modeling, and statistical hypothesis testing (using Wilcoxon rank-sum test) (Fig. 1).

Household-level quantities consumed data can help quantify food consumption diversity^39–41^. Here, food diversity is based on whether a household consumes a given commodity (Supplementary Text S2). The analysis uses binary outcome data for 124 food commodities combined into 13 food groups (*n*) to calculate a food diversity index using Eq. (1).

1 $${Food\,Diversity\,Index}_{i}= -\sum_{j=1}^{n}{p}_{i}\times log\left({p}_{i}\right)$$

where *p*_*i*_ is the proportion of food commodities consumed per *j*th food group by a household (*i*). A higher value of the food diversity index implies greater consumption diversity—higher different types and even distribution of food commodities consumed across food groups (Supplementary Text S2). In addition to urban–rural differences in the quantities and diversity of food consumed, we used a supplementary correlation analysis to investigate the associations between urbanization (share of the urban population to the total population) and food consumption (quantities and diversity). We then compared these correlations with correlations between income and food consumption. Additionally, we estimated OLS regression models to examine urbanization influences for food commodities where urban–rural differences indicated a possible influence. We also estimated an OLS model for food consumption diversity. We accounted for confounding factors, including income and state-level effects, to examine if factors beyond these influence food consumption. Furthermore, we estimated multilevel models with a three-level specification (household, district, and state) and examined the robustness of the results. We also contrasted multilevel models containing household-level characteristics with urbanization and urbanization-related variables (at the district- and state-levels) to investigate multidimensional urbanization influences on consumption diversity. The comparison criteria included interclass correlation coefficients (ICC), the magnitude and significance of the predictors, and the overall explanatory power of the models. Finally, we used the Wilcoxon rank-sum test to compare average levels of household characteristics between UM, UNM, and R areas and examined bivariate distributions of aggregated variables at the district level to assess broader urbanization influences. Section S2 in the supplementary text provides a detailed methodological description.

## Results

### Quantities of food consumed

**Figure 2:**
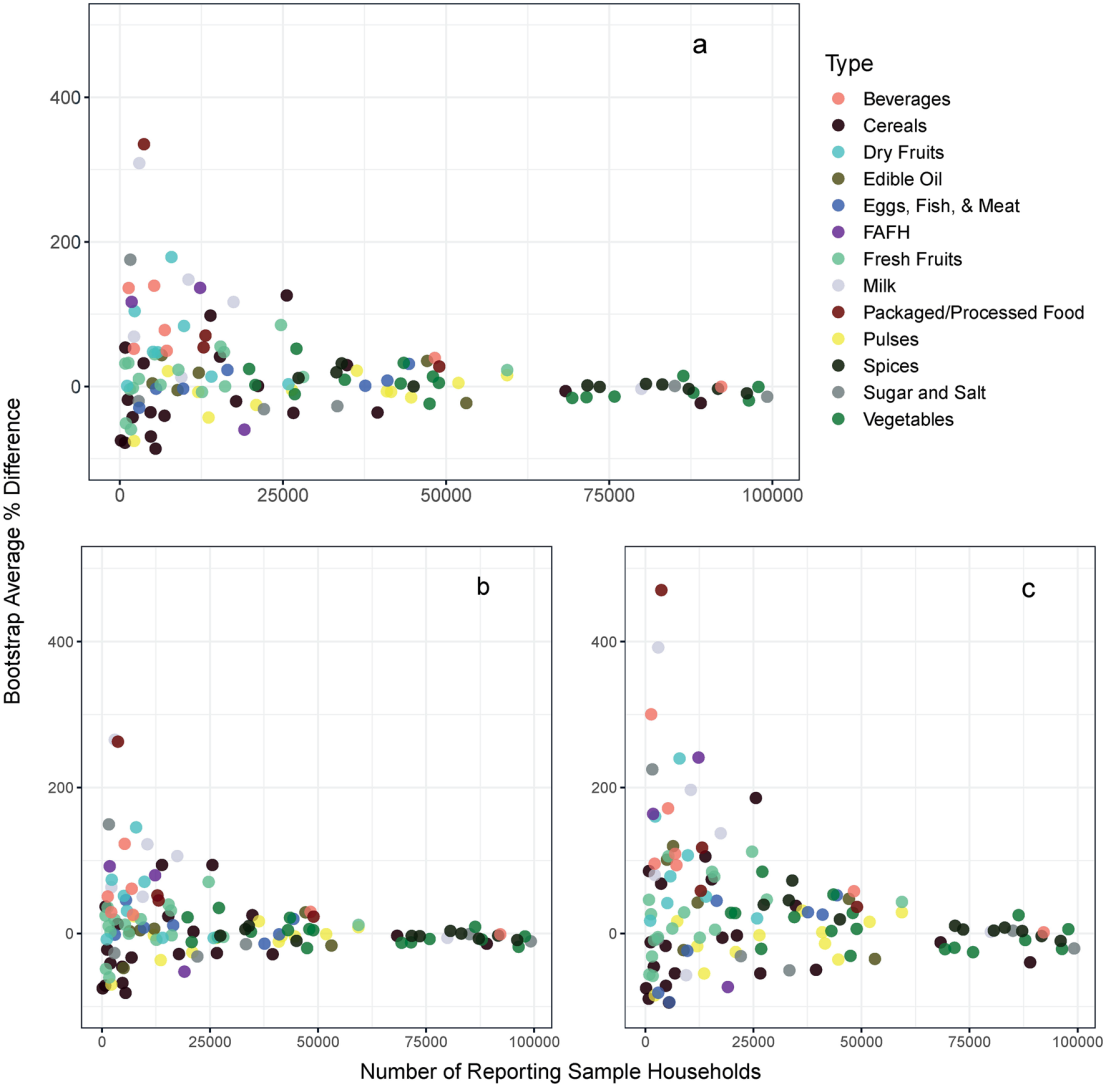
Bootstrap percentage differences in average household consumption by which consumption (**a**) in urban areas exceeds consumption in rural areas, (**b**) in (urban) non-metropolitan areas exceeds consumption in rural areas, and (**c**) in (urban) metropolitan areas exceeds consumption in rural areas.

**Figure 3:**
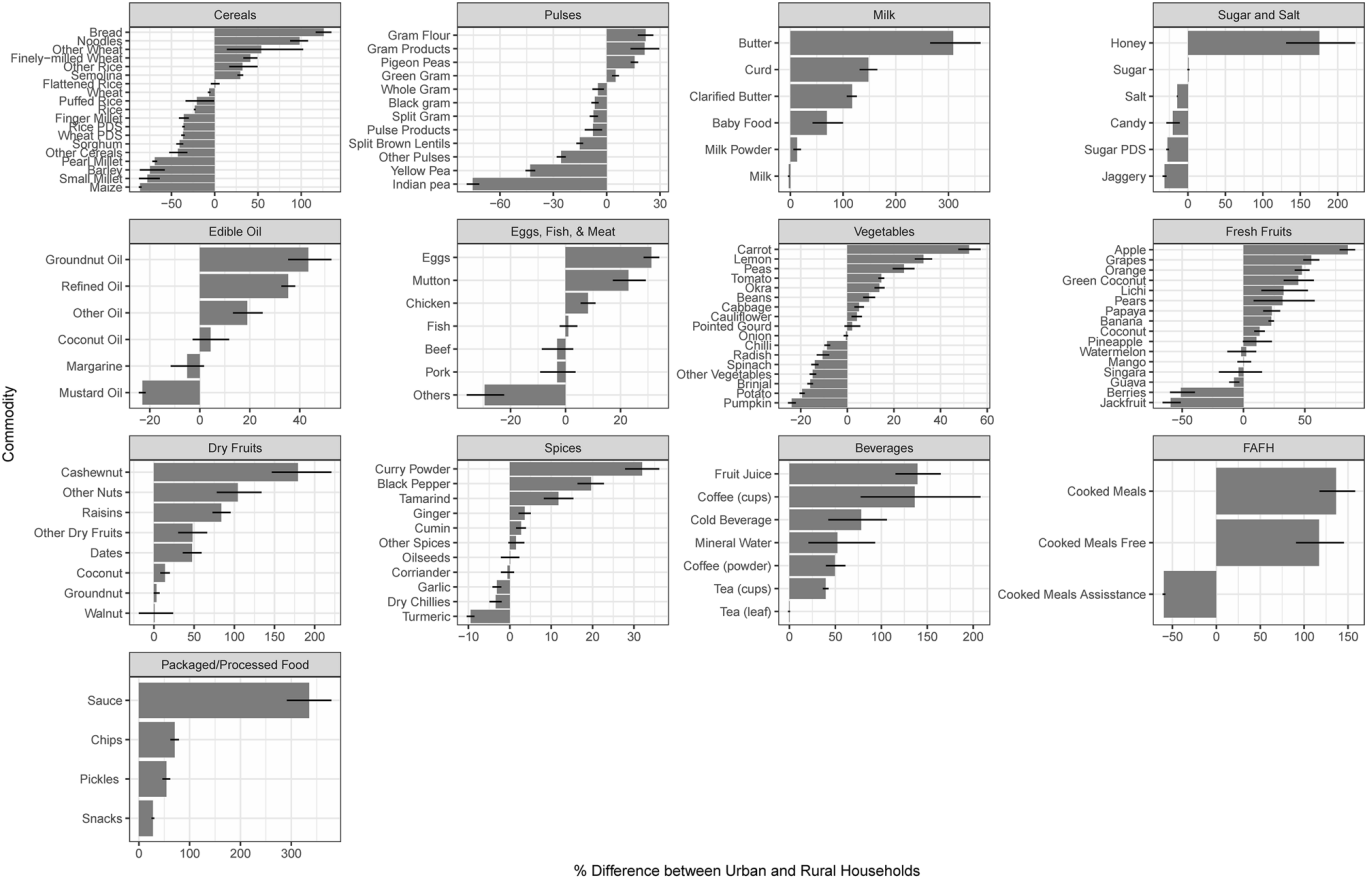
Bootstrap difference in average household consumption between urban and rural areas by commodity (n = 124) and commodity types. The x-axes show average bootstrap estimates of percentage difference by which average consumption by households in urban areas differs from households in rural areas. Positive values indicate greater average urban household consumption. The black line at the end of each bar shows the 95% confidence interval.

Much of the variations in food consumption quantity is due to income differences and not urban–rural differences or urbanization. This finding contrasts the conventional understanding that urbanization leads to higher consumption of certain food commodities. Results show that the average household consumption is higher amongst urban households than rural households for 62 of 124 food commodities, at the 0.05 significance level (Fig. 2a). These commodities include edible oil, spices, fruits and vegetables, dairy products, meat (eggs, chicken, and mutton), and processed foods (Fig. 3). These urban–rural differences are consistent with the literature that shows urbanization influences consumers to move away from traditional staples, towards increased consumption of fruits and vegetables, meat, food prepared away from home, and processed/packaged foods^42,43^. However, after controlling for income, results suggest that the urban–rural differences are significant for only eight commodities that are also consumed by fewer households, at the 0.05 significance level (Table S1). After also controlling for state-level differences, we find 12 commodities where average consumption is higher in urban areas at 0.05 significance level (Table S2). Overall, these results suggest that only living in urban areas does not increase the quantities of food consumed.

Differences between average quantities consumed in UM, UNM, and R areas also suggest that merely living in more urbanized areas is not associated with higher quantities of food consumed. Results indicate increased average consumption in UM areas followed by UNM and R areas for 19 commodities (Fig. 2b,c), with a more significant positive difference for commodities consumed by fewer households. Figures S4–S7 show a detailed comparison. After controlling for income, urban influence exists only for four food commodities (Table S3). After also controlling for state-level differences, results suggest three commodities where the average consumption increase follows the urban progression (UM > UNM > R areas), at the 0.05 significance level (Table S4). A robustness check from examining multilevel models further emphasizes limited positive urbanization influence (Tables S5–S66). Only eight commodities indicate a statistically significant urban effect after controlling for several household- and district-level characteristics.

**Figure 4:**
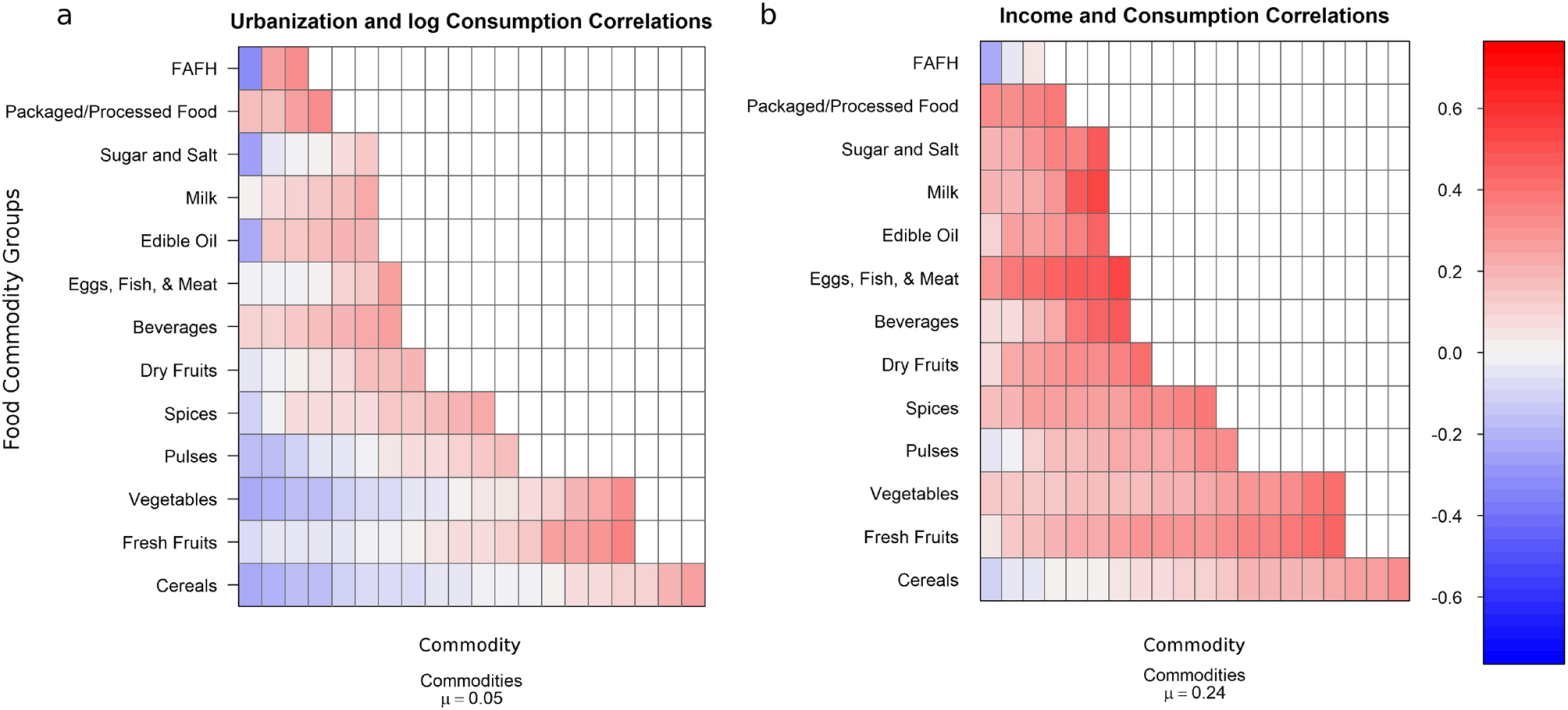
Correlations between (**a**) urbanization (share of urban population to total population) and household consumption (bootstrapped weighted averages) at the district level and (**b**) household income and household consumption. Each cell in the plots represents a commodity. Commodities µ is the mean correlation across all commodities.

We obtained similar results when examining the associations between the urban share of the total population and the quantities consumed. Cross-sectional variations in urbanization only weakly explain the variations in average quantities consumed by households across 124 commodities (Fig. 4a). Correlations between urbanization and average quantities of consumption are less than 0.60 at the state level and 0.33 at the district level. Household income better, albeit moderately, explains the variations in quantity and diversity of consumption at the household level (Fig. 4b): absolute values of Pearson’s correlation coefficient are less than 0.52. For the 124 commodities examined, correlations are between 0.40 and 0.52 for 13 commodities ranging from milk, sugar, edible oil, fresh and dry fruits, and beverages to eggs, fish, and meat.

### Diversity of food consumed

Contrary to the existing literature, results show that urbanization as a demographic share has an insignificant influence on food consumption diversity, after controlling for household-level characteristics. Instead, urbanization-related variables such as market access, infrastructure, percentage of urban women working, and norms and institutions better explain food consumption diversity compared with urbanization as a demographic share. Overall, a more nuanced relationship exists between urbanization and food consumption diversity in India than that described by an urban–rural dichotomy or urban population share.

**Figure 5:**
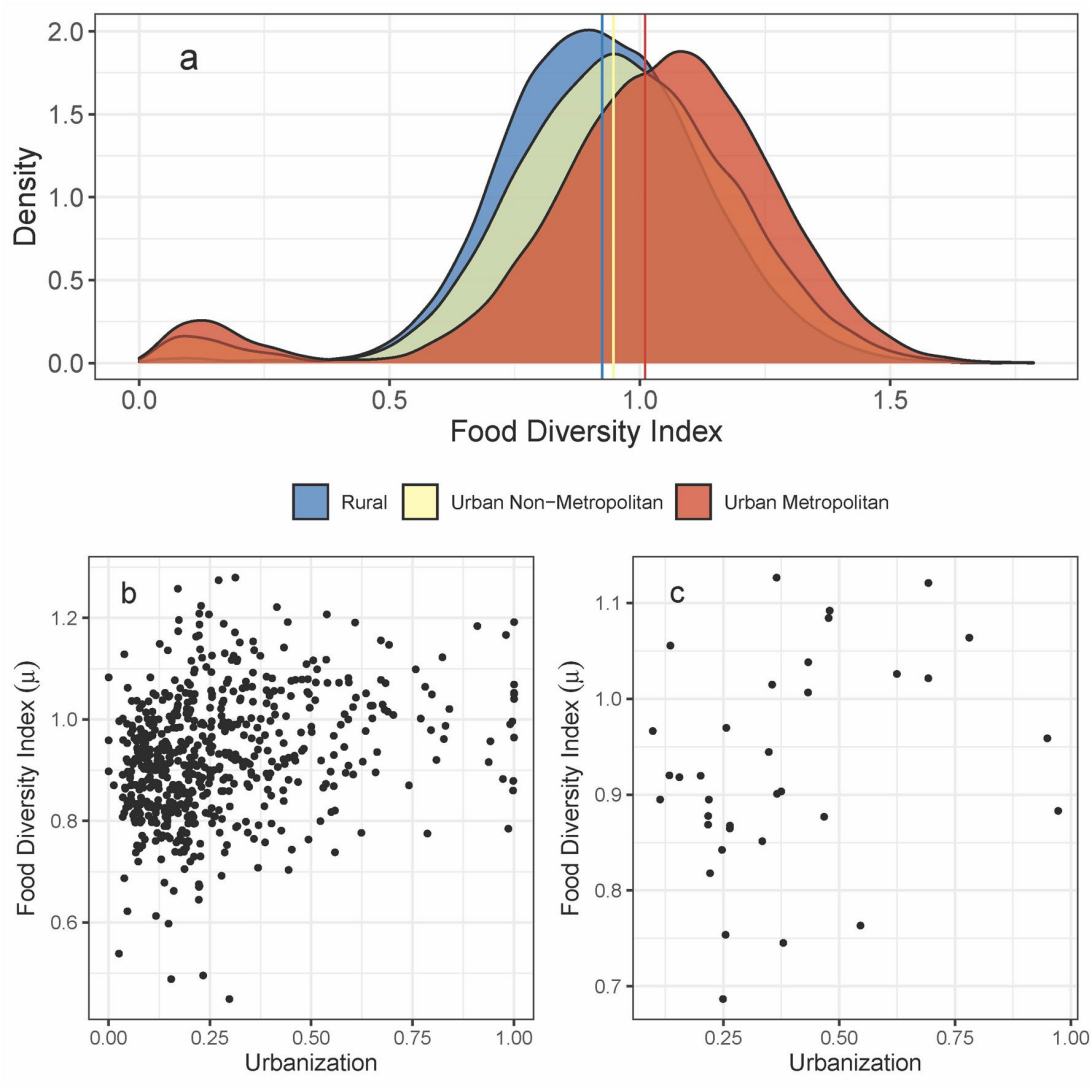
(**a**) Distribution of food diversity index for sample households in rural (59,695), urban non-metropolitan (27,333), and urban metropolitan areas (14,634), respectively. Vertical lines show the average of bootstrap estimates for the respective group of households. Average diversity indices for households in urban non-metropolitan and rural areas are more similar to each other than to the households in urban metropolitan areas is confirmed by a non-parametric Wilcoxon rank-sum test (p-values < 0.01). Diversity index (average entropy) and urbanization (share of urban population to total population) at the (**b**) district and (**c**) state-level.

The average food diversity index is 2.43% higher for UNM households as compared to R households and 6.67% higher for households in UM than in UNM areas (Fig. 5a). The household food diversity index is also moderately related to household income (*r* = 0.51). We find that the average food diversity index is higher for UM areas, followed by UNM areas and R areas, even after controlling for income and state-level differences (Table 1). These results corroborate with Popkin’s nutrition transition theory; diets are more varied in urban areas where technological proliferation and service sector development are more prevalent than in rural areas^43^. Increasing diversity related to urbanization may also be concomitant with supply chain spatial extension, resulting in higher food availability and access with higher urbanization levels^42,44^. Additionally, the results are consistent with the existing literature that has emphasized the positive influence of living in urban areas on food consumption diversity. However, the results show that urbanization influence is not due to increasing urban share to the total population but involves more nuanced changes that characterize urbanization.

**Table 1:** Regression estimates for household’s food diversity with (log) household income, urbanization levels (relative to rural), and high diversity states dummy variables relative to single-individual households as covariates.

	Food Diversity Index
Household income	0.13*** (0.001)
Urban non-metropolitan	0.01*** (0.001)
Urban metropolitan	0.02*** (0.002)
Diversity states	0.18*** (0.001)
Constant	− 0.28*** (0.01)
F Statistic (df = 2; 464,957)	16,732.25***
Observations	94,796
R^2^	0.42
Adjusted R^2^	0.42
Residual Std. Error	0.15 (df = 94,791)

***p < 0.01, Robust standard errors.

Multilevel null models suggest 41% and 36% of the total variation in the household-level food diversity index is due to between-districts and between-states variation, respectively (Table 2, Models 1–2). For the three-level model, 45% of the total variation is due to between states (35%) and districts within states (10%) (Table 2, Model 3). These baseline results suggest significant between-group variations at the state and district levels but that the three-level model captures more variations at the state and district levels. A chi-square test further confirms the three-level model as the best fit (p-value < 0.01). Accordingly, the three-level model suffices for a baseline model. Results obtained using the baseline model suggest that household income, household structure, access to a cooking facility, and place of residence all positively influence food diversity (Table 3, Model 1). Household size, on the other hand, has a negative influence. The direction of these correlations also corroborates with the literature. At aggregate scales, results show a modest but statistically significant correlation between urbanization and food consumption diversity: Pearson’s correlation coefficients are 0.30 (*p-value* < 0.01) and 0.29 (*p-value* < 0.01) at the state and district levels, respectively (Fig. 5b,c). However, after controlling for household characteristics under the multilevel model specification, the district-level urbanization variable shows an insignificant influence on the food diversity index. Furthermore, it does little to improve the model fit over the baseline model with household characteristics (Table 3, Model 1 and 2). In contrast, adding district- and state-level variables that capture different aspects of urbanization—infrastructure, market access, percentage of urban women working, and norms and institutions—significantly improves the model fit (in terms of AIC, BIC, and Adjusted R^2^ estimates) (Table 3, Model 3). *Ceteris paribus,* districts closer to major cities, have higher food diversity than those located farther away. Similarly, districts with more women working in urban areas have lower food diversity. Contrary to expectation, results show an insignificant influence of the infrastructure variable. Nonetheless, this may be due to the correlation between infrastructure and market access (*ρ* = − 0.71). Here infrastructure positively influences food consumption diversity after dropping the market access variable (Table 3, Model 4 and 5).

**Table 2:** Unconditional means model (null model) estimates.

	Food Diversity Index		
(Intercept)	0.947*** (0.937–0.957)	0.957*** (0.917–0.998)	0.952*** (0.910–0.993)
**Random effects**			
σ^2^	0.023	0.027	0.023
${\tau }^{2}/{{\tau }_{1}}^{2}$	0.016_district_	0.015_state_	0.004_district: state_
${{\tau }_{2}}^{2}$			0.015_state_
ICC	0.41	0.36	0.45
N	619 _district_	35_state_	619 _district_
			35_state_
Observations	94,796	94,796	94,796
Marginal R^2^/Conditional R^2^	0.000 / 0.407	0.000 / 0.361	0.000 / 0.451

***p < 0.001.

**Table 3:** Linear mixed-effects models for food consumption diversity in India using the household-, district-, and state-level covariates.

	Food Diversity Index				
	(1)	(2)	(3)	(4)	(5)
Intercept	− 0.3956** (− 0.4384 to − 0.3527)	− 0.3956** (− 0.4395 to − 0.3517)	− 0.3745** (− 0.4589 to − 0.2901)	− 0.3583** (− 0.4285 to − 0.2881)	− 0.4565** (− 0.4951 to − 0.4180)
Household income	0.1475** (0.1459–0.1491)	0.1475** (0.1459–0.1491)	0.1475** (0.1459–0.1491)	0.1475** (0.1459–0.1491)	0.1475** (0.1459–0.1491)
Household size	− 0.0130** (− 0.0155 to − 0.0105)	− 0.0130** (− 0.0155 to − 0.0105)	− 0.0130** (− 0.0155 to − 0.0105)	− 0.0130** (− 0.0155 to − 0.0105)	− 0.0130** (− 0.0155 to − 0.0105)
Household structure	0.0139** (0.0122–0.0156)	0.0139** (0.0122–0.0156)	0.0139** (0.0122–0.0156)	0.0139** (0.0122–0.0156)	0.0139** (0.0122–0.0156)
Cooking facility	0.0366** (0.0346–0.0387)	0.0366** (0.0346–0.0387)	0.0367** (0.0346–0.0387)	0.0367** (0.0346–0.0387)	0.0366** (0.0346–0.0387)
Urban non-metropolitan	0.0070** (0.0049–0.0091)	0.0070** (0.0049–0.0091)	0.0070** (0.0049–0.0091)	0.0070** (0.0049–0.0091)	0.0070** (0.0049–0.0091)
Urban metropolitan	0.0080** (0.0043–0.0118)	0.0080** (0.0042–0.0118)	0.0080** (0.0043–0.0118)	0.0081** (0.0043–0.0118)	0.0081** (0.0043–0.0119)
Urbanization		0.0000 (− 0.0283 to 0.0284)			
Infrastructure			0.0011 (− 0.0021 to 0.0043)		0.0031* (0.0005–0.0057)
Travel time to cities			− 0.0144* (− 0.0275 to − 0.0012)	− 0.0170** (− 0.0278 to − 0.0061)	
% Working women in urban areas			− 0.4487* (− 0.8257 to − 0.0717)	− 0.3897* (− 0.7294 to − 0.0501)	− 0.4302* (− 0.8079 to − 0.0526)
Diversity states			0.1699** (0.1083–0.2315)	0.1721** (0.1102–0.2339)	0.1695** (0.1078–0.2312)
**Random effects**					
σ^2^	0.01	0.01	0.01	0.01	0.01
(τ_2_)^2^	0.00_District:State_	0.00_District:State_	0.00_District:State_	0.00_District:State_	0.00_District:State_
(τ_1_)^2^	0.01_State_	0.01_State_	0.01_State_	0.01_State_	0.01_State_
ICC	0.55	0.55	0.41	0.41	0.41
N	619 _District_	619_District_	619_District_	619_District_	619_District_
	35_State_	35_State_	35_State_	35_State_	35_State_
Observations	94,796	94,796	94,796	94,796	94,796
Marginal R^2^/Conditional R^2^	0.250/0.661	0.250/0.661	0.441/0.670	0.440/0.671	0.442/0.671

*p < 0.05, **p < 0.01.

Differences between ICC estimates of different models further support our interpretation. The ICC estimate is expected to decrease upon adding district-level or state-level variables or both to the baseline model containing variables that account for household-level characteristics. However, results show no change in the ICC estimate upon adding the district-level urban share to the total population variable (Table 3, Model 1–2). The unchanged ICC estimate suggests that urbanization as a demographic share does not explain the variations in food consumption diversity. In contrast, adding variables that capture different aspects of urbanization reduces the ICC estimate by ~ 25% compared to the model with household characteristics only, indicating the importance of multiple urbanization process pathways (Table 3, Model 1 and 3).

### Multidimensional urbanization influences

**Figure 6:**
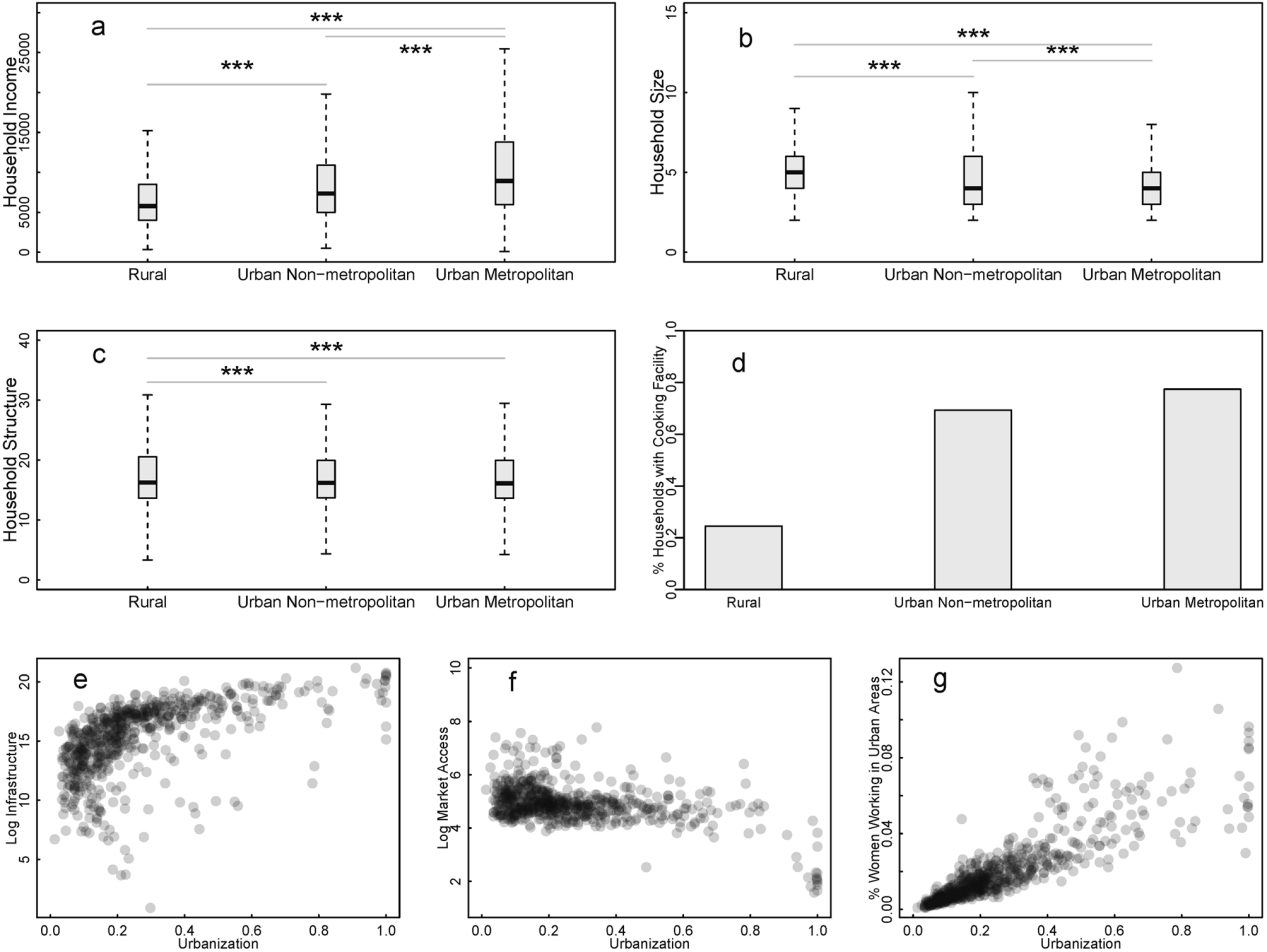
(**a**) Household income, (**b**) household size, (**c**) household structure, (**d**) % of households with cooking facility, (**e**) infrastructure, (**f**) market access and (**g**) % urban women working as correlates of urbanization. Horizontal bars in (**a**–**c**) show statistically-significant differences across the three groups (rural, urban non-metropolitan, and urban metropolitan). Statistical significance tests are based on the Wilcoxon rank-sum test (p-value < 0.01).

All the multi-scalar food consumption diversity determinants examined in the analysis are also associated with urbanization characterized as a demographic share or as the urban–rural difference (Fig. 6). Whereas household size and structure generally decrease with living in urban areas, results show that household income and access to a cooking facility generally increase. Similarly, infrastructure, market access, and the percentage of working women in urban areas are all associated with urbanization. States with high food consumption diversity also have districts with higher urbanization levels than lower food consumption diversity states: mean urbanization levels of the districts are 44% in states with high diversity and 26% in states with low diversity. Non-parametric Wilcoxon rank-sum tests confirm that these differences are statistically significant (p-values < 0.01). These results suggest that economic (income), demographic (household size and structure), socio-institutional (differences between states in food consumption diversity), and spatial (infrastructure and market access) dimensions of urbanization all play a role in shaping food consumption diversity in India.

## Discussion

Although a large body of work exists that examines the influence of urbanization on food consumption and diets, we have a limited understanding of the underlying pathways and mechanisms. It is unclear whether any universal pathway(s) exist and whether these influences differ across urbanizing regions by magnitude or characteristics or both. This understanding is especially important for rapidly urbanizing countries such as India. In this context, our analysis of urbanization and food consumption in India reveals several insights with implications for our collective understanding of urbanization and food consumption relationships.

*First*, the results suggest that examining urbanization influences through urban population shares to total population or urban–rural differences are insufficient to determine the broad-range of urbanization influences at play. They highlight both conceptual and methodological limitations of previous studies that emphasize urbanization influences on food consumption based solely on these variables. Urbanization is comprised of changes across multiple dimensions: demographic, spatial, economic, social, and cultural^45^. Urban–rural differences and demographic share did not help to identify and study the influence of these changes on consumption in the present study. The present study shows that much of the variation in average quantities consumed is due to income differences, with a limited role of urbanization (as a share of the total population and living in urban areas). Moreover, urbanization, as a demographic share, has an insignificant influence on food consumption diversity. In contrast, variables that explain food consumption diversity—such as increased market access, the increased value of women’s time while working in urban areas, infrastructure, and social norms (differences in food consumption diversity between states)—are closely intertwined with urbanization. Nonetheless, existing studies focusing on urbanizing countries seldom interpret the effects of these variables as urbanization-related.

*Second*, examining urbanization influences using measures predicated on administrative classifications can omit indirect influences leading to over or underestimation of the impacts. Results from the present study, consistent with Bennett’s law, suggest that our shared understanding of widespread increases in the consumption of animal-based products, edible oils, sugar, and other food commodities due to urbanization as a demographic share requires further consideration. Urbanization is frequently associated with changes in diets in high-level policy discourses, such as in the 2019 Eat-Lancet Commission Report^46^ and the 2019 and 2020 IFPRI Global Food Policy Reports^47,48^. Previous studies focusing on India have similarly attributed changes in average quantities of cereals, fruits and vegetables, animal-based products, sugar, edible oils, processed foods, beverages, and other food commodities consumed to urbanization^49,50^. Findings from this study suggest that urbanization, as a demographic share, may not be directly associated with changes in the quantities of food commodities consumed. Instead, urbanization and income are related in India, and urbanization influences on the quantities and diversity of food consumed may be indirect and influence quantities consumed through the income pathway. Similarly, other variables examined in the present study can also extend indirect influence.

*Third*, urbanization may have a more considerable influence on the diversity of food consumed than quantities in India. Results from the present study suggest that demographic, spatial, and institutional factors that could lead to significant urban–rural differences in food quantities consumed may not yet be pronounced in India, given its preliminary stages of urbanization. However, India, a lower-middle-income country, is projected to add ~ 400 million urban dwellers by 2050. Its urban land area has been forecasted to increase by up to 156,000 km^2^ by 2050, more than 500% increase over the 2000 extent^51^. These demographic and land changes will accompany other significant changes: demographic (household size and structure), economic (income), social (social ties and interactions), institutional (socio-cultural norms and regulations), spatial (infrastructure and built environment), and technological (efficiency gains in food production and food supply chains). The results suggest that all of these changes can influence food consumption. Consequently, results suggest that future urbanization in India could lead to significant increases in the household consumption of approximately half of the commodities considered in the study, which have positive income elasticity and significant urban–rural differences before controlling for income. These are a diverse set of commodities including processed foods and beverages (snacks, fruit juice, cold beverages, and others), animal-based products (eggs, chicken, and mutton), milk-based products (clarified butter, curd, and others), edible oil (refined oil), dry and fresh fruits, and others. With impending urbanization, results suggest that diet diversification in India could be fueled partly by the spatial dimensions of urbanization, such as infrastructure growth and improved market access.

*Fourth*, although the present results show broader urbanization influences in the case of food consumption diversity than quantities consumed in India, urbanization may also be leading to changes in other aspects of food consumption such as food away from home, food waste, and the consumption of value-added food commodities not considered in this study. Understanding these linkages will require a systematic investigation of different aspects of urbanization. However, to date, no conceptual model exists in the scientific literature to explain how different urbanization processes synergistically influence food consumption. Therefore, our results underline the need for a new conceptualization of urbanization and urbanization influences on food consumption. This conceptualization could play a pivotal role in scientific investigations aimed towards identifying specific leverage points to bring positive dietary changes within the purview of global urbanization.

The present study has some limitations that warrant further investigation. Findings from this study are based on correlations drawn from a single snapshot survey dataset and do not imply causation. Furthermore, this study only focused on regional variations in infrastructure, market access, and the percentage of urban women working. Significant heterogeneities at the intra-urban scale can exist along these dimensions, which are not sufficiently captured in the national survey used here. The survey lacks sufficient spatial detail, due to limited sample size, and restricts analysis to regional variations in food consumption, as opposed to intra-urban variations. Similarly, other aspects of urbanization, such as spatial urban form, the level of residence-workplace colocation, and travel and commuting behavior, remain unexplored, yet can influence food consumption^9^. From the food consumption standpoint, the survey dataset provides limited details on the food commodities consumed, which can influence the quantities and diversity estimates. The survey dataset used is also prone to recall errors due to the 30-day recall period. Finally, in the absence of a comprehensive conceptual framework to guide an analysis of urbanization influences on food consumption, the nature of the findings from the present study is more exploratory than confirmatory.

## Conclusions and future prospects

The present study reports findings from a detailed analysis of urbanization influences on the quantities of food consumed (across 124 commodities) and food consumption diversity in India. Contrasting with the existing literature, urban–rural differences estimated after controlling for income and state-level effects show limited evidence towards the impact of living in urban areas on the quantities of food consumed in India. Furthermore, results comparing average consumption between UM, UNM, and R areas also show limited urbanization influence. The study also finds a statistically insignificant influence of urbanization on food consumption diversity, which is surprising since past studies have frequently emphasized the influence of urbanization on food consumption diversity or dietary diversification. Instead, variables related to urbanization have a statistically significant influence—infrastructure, market access, percentage working women in urban areas, and norms and institutions. This study suggests that urbanization influences can be indirect and multidimensional. Based on these findings, we draw two broad conclusions. *First*, urban–rural differences and demographic shares are insufficient to determine the broad-range of urbanization influences at play. *Second*, besides income and other household characteristics, food consumption also depends on the spatial dimensions of urbanization, including market access and infrastructure.

These conclusions emphasize the need for a systematic examination of urbanization influences on food consumption and diets going forward. A systematic review supplemented with empirical analysis can lead to a comprehensive framework to contemplate the role of urbanization in shaping food consumption. Furthermore, comparative studies can advance existing knowledge. Here at least two questions that remain unanswered are: (1) Why does urbanization lead to different or similar food consumption outcomes within and across regional contexts? (2) What are the current and future human health and environmental sustainability implications of the differentiated outcomes caused by urbanization? Besides, spatially-detailed investigations are needed to examine inter- and intra-urban variations in food consumption. This will require a targeted, multi-city survey of food consumption and urban dimensions. Overall, as urban areas increasingly play a significant role in shaping our food behavior, a better understanding of how urbanization changes what, where, and when we consume can inform whether a sustainable urban food system future exists and what can be done now to achieve it.

## Supplementary information

Supplementary Information.

## Data Availability

Data aggregated from multiple sources that support the findings of this study are available from the corresponding author upon reasonable request. Detailed household-level data on consumer expenditure is available from https://mospi.nic.in/.

## Code availability

R scripts used in this study are available from the corresponding author upon reasonable request.
