# Supplementary material for: Urbanization and food consumption in India
Source: Sci Rep. 2020 Oct 14;10:17241. doi: 10.1038/s41598-020-73313-8 (PMC7560883; doi:10.1038/s41598-020-73313-8)
Supplement: Supplementary file 1 — Supplementary Information. [file 41598_2020_73313_MOESM1_ESM.docx]

**Supplementary Information**

**Urbanization and Food Consumption in India**

Bhartendu Pandey^a^, Meredith Reba^a^, P. K. Joshi^b, c^, Karen C. Seto^a^

^a^ Yale School of the Environment, Yale University, New Haven, CT 06511, U.S.A.

^b^ School of Environmental Sciences, Jawaharlal Nehru University, New Delhi, Delhi 110067, India

^c^ Special Centre for Disaster Research, Jawaharlal Nehru University, New Delhi, Delhi 110067, India

Table S1: Regression estimates for (log) household consumption of 62 food commodities with (log) household income and urban (relative to rural) variables as covariates.

| Food Item | Food Item Type | Intercept | Income | Urban | R^2^ |
| --- | --- | --- | --- | --- | --- |
| Other Rice | Cereals | 4.789*** | 0.291*** | -0.114*** | 0.04 |
| Finely-milled Wheat | Cereals | 4.505*** | 0.269*** | -0.072*** | 0.05 |
| Semolina | Cereals | 4.331*** | 0.268*** | -0.04*** | 0.06 |
| Noodles | Cereals | 3.191*** | 0.329*** | -0.031** | 0.07 |
| Bread | Cereals | 2.907*** | 0.43*** | 0.048*** | 0.1 |
| Other Wheat | Cereals | 6.073*** | 0.093* | -0.008 | 0 |
| Pigeon Peas | Pulses | 4.81*** | 0.272*** | -0.101*** | 0.06 |
| Green Gram | Pulses | 4.023*** | 0.294*** | -0.199*** | 0.08 |
| Gram Products | Pulses | 2.759*** | 0.373*** | -0.124*** | 0.11 |
| Gram Flour | Pulses | 3.172*** | 0.351*** | -0.102*** | 0.1 |
| Baby Food | Milk | 4.628*** | 0.222*** | -0.01 | 0.04 |
| Milk Powder | Milk | -0.224 | 0.719*** | -0.047*** | 0.22 |
| Curd | Milk | 2.961*** | 0.452*** | -0.15*** | 0.08 |
| Clarified Butter | Milk | 2.709*** | 0.386*** | -0.095*** | 0.06 |
| Butter | Milk | 3.179*** | 0.251*** | -0.091*** | 0.04 |
| Honey | Sugar and Salt | 2.004*** | 0.318*** | -0.006 | 0.05 |
| Groundnut Oil | Edible Oil | 2.861*** | 0.546*** | 0.002 | 0.19 |
| Refined Oil | Edible Oil | 4.626*** | 0.322*** | 0 | 0.07 |
| Other Oil | Edible Oil | 5.86*** | 0.153*** | 0.069*** | 0.01 |
| Eggs | Eggs, Fish, & Meat | -1.497*** | 0.461*** | 0.01* | 0.21 |
| Mutton | Eggs, Fish, & Meat | 2.451*** | 0.504*** | -0.126*** | 0.19 |
| Chicken | Eggs, Fish, & Meat | 2.972*** | 0.479*** | -0.112*** | 0.21 |
| Tomato | Vegetables | 4.46*** | 0.35*** | -0.023*** | 0.1 |
| Carrot | Vegetables | 3.643*** | 0.357*** | -0.072*** | 0.09 |
| Okra | Vegetables | 4.391*** | 0.296*** | -0.124*** | 0.07 |
| Cauliflower | Vegetables | 5.728*** | 0.191*** | -0.19*** | 0.04 |
| Cabbage | Vegetables | 5.619*** | 0.185*** | -0.15*** | 0.03 |
| Peas | Vegetables | 5.177*** | 0.22*** | -0.161*** | 0.03 |
| Beans | Vegetables | 4.59*** | 0.266*** | -0.177*** | 0.06 |
| Lemon | Vegetables | -1.547*** | 0.396*** | -0.053*** | 0.13 |
| Banana | Fresh Fruits | -0.797*** | 0.419*** | -0.061*** | 0.17 |
| Coconut | Fresh Fruits | -1.265*** | 0.338*** | -0.022* | 0.06 |
| Green Coconut | Fresh Fruits | -0.719*** | 0.244*** | -0.132*** | 0.05 |
| Orange | Fresh Fruits | -1.011*** | 0.374*** | -0.078*** | 0.12 |
| Papaya | Fresh Fruits | 4.771*** | 0.3*** | -0.157*** | 0.1 |
| Pears | Fresh Fruits | 3.784*** | 0.362*** | -0.23*** | 0.1 |
| Lichi | Fresh Fruits | 3.422*** | 0.387*** | -0.054 | 0.12 |
| Apple | Fresh Fruits | 2.533*** | 0.487*** | -0.033*** | 0.18 |
| Grapes | Fresh Fruits | 2.818*** | 0.422*** | -0.004 | 0.15 |
| Coconut | Dry Fruits | 2.001*** | 0.398*** | -0.07*** | 0.1 |
| Dates | Dry Fruits | 3.03*** | 0.308*** | -0.129*** | 0.06 |
| Cashewnut | Dry Fruits | -0.755*** | 0.578*** | 0.068*** | 0.17 |
| Other Nuts | Dry Fruits | 0.72*** | 0.484*** | -0.2*** | 0.13 |
| Raisins | Dry Fruits | 1.019*** | 0.404*** | 0.007 | 0.1 |
| Other Dry Fruits | Dry Fruits | 1.531*** | 0.393*** | -0.085*** | 0.06 |
| Ginger | Spices | 1.699*** | 0.406*** | -0.108*** | 0.12 |
| Cumin | Spices | 1.478*** | 0.326*** | -0.066*** | 0.11 |
| Black Pepper | Spices | 1.439*** | 0.269*** | -0.042*** | 0.06 |
| Tamarind | Spices | 3.497*** | 0.214*** | -0.094*** | 0.03 |
| Curry Powder | Spices | 1.596*** | 0.31*** | -0.01 | 0.07 |
| Tea (cups) | Beverages | 0.528*** | 0.262*** | 0.206*** | 0.06 |
| Coffee (cups) | Beverages | 0.347 | 0.208*** | 0.006 | 0.03 |
| Coffee (powder) | Beverages | 3.627*** | 0.118*** | -0.227*** | 0.02 |
| Mineral Water | Beverages | 6.641*** | 0.235*** | -0.043 | 0.01 |
| Cold Beverage | Beverages | 1.963*** | 0.575*** | -0.022 | 0.19 |
| Fruit Juice | Beverages | 2.257*** | 0.503*** | 0.022 | 0.14 |
| Cooked Meals | FAFH | 3.236*** | -0.114*** | 0.346*** | 0.03 |
| Cooked Meals Free | FAFH | 5.227*** | -0.268*** | 0.418*** | 0.1 |
| Snacks | Packaged/Processed Food | 1.842*** | 0.441*** | 0.017** | 0.14 |
| Chips | Packaged/Processed Food | 0.659*** | 0.46*** | 0.049*** | 0.1 |
| Pickles | Packaged/Processed Food | 1.279*** | 0.428*** | -0.12*** | 0.12 |
| Sauce | Packaged/Processed Food | 1.192*** | 0.432*** | 0.056* | 0.1 |
| Note:  ***p<0.01; **p<0.05; *p<0.1  Robust Standard Errors | | | | | |

Table S2: Regression estimates for (log) household consumption of 62 food commodities with (log) household income, urban (relative to rural), and state dummy variables as covariates.

| Food Item | Food Item Type | Intercept | Income | Urban | R^2^ |
| --- | --- | --- | --- | --- | --- |
| Other Rice | Cereals | 5.724*** | 0.289*** | -0.115*** | 0.24 |
| Finely-milled Wheat | Cereals | 4.536*** | 0.295*** | -0.06*** | 0.2 |
| Semolina | Cereals | 3.243 | 0.337*** | -0.065*** | 0.27 |
| Noodles | Cereals | 2.657 | 0.368*** | -0.028*** | 0.13 |
| Bread | Cereals | 4.185*** | 0.408*** | 0.073*** | 0.34 |
| Other Wheat | Cereals | 6.986*** | 0.127*** | -0.045*** | 0.23 |
| Pigeon Peas | Pulses | 3.277*** | 0.384** | -0.076 | 0.32 |
| Green Gram | Pulses | 3.637 | 0.322*** | -0.152*** | 0.18 |
| Gram Products | Pulses | 3.354 | 0.37 | -0.104*** | 0.21 |
| Gram Flour | Pulses | 2.32*** | 0.38*** | -0.092*** | 0.22 |
| Baby Food | Milk | 4.176 | 0.278*** | 0.029*** | 0.13 |
| Milk Powder | Milk | 1.805 | 0.544** | 0.015 | 0.44 |
| Curd | Milk | 4.196*** | 0.434*** | -0.127*** | 0.29 |
| Clarified Butter | Milk | 1.867*** | 0.502*** | -0.036*** | 0.56 |
| Butter | Milk | 3.189 | 0.317*** | -0.029*** | 0.31 |
| Honey | Sugar and Salt | 2.295 | 0.3 | -0.05 | 0.22 |
| Groundnut Oil | Edible Oil | 4.641*** | 0.412*** | -0.09*** | 0.45 |
| Refined Oil | Edible Oil | 3.004 | 0.43*** | -0.02*** | 0.53 |
| Other Oil | Edible Oil | 4.377 | 0.242 | 0.015*** | 0.55 |
| Eggs | Eggs, Fish, & Meat | -1.487 | 0.464*** | 0.007*** | 0.25 |
| Mutton | Eggs, Fish, & Meat | 2.791*** | 0.503*** | -0.096*** | 0.26 |
| Chicken | Eggs, Fish, & Meat | 3.446 | 0.447*** | -0.1*** | 0.26 |
| Tomato | Vegetables | 3.904*** | 0.412*** | -0.027*** | 0.26 |
| Carrot | Vegetables | 4.364*** | 0.339*** | -0.026*** | 0.34 |
| Okra | Vegetables | 4.562*** | 0.332*** | -0.087*** | 0.24 |
| Cauliflower | Vegetables | 5.289*** | 0.266*** | -0.1*** | 0.3 |
| Cabbage | Vegetables | 5.391*** | 0.246*** | -0.088*** | 0.32 |
| Peas | Vegetables | 4.777*** | 0.293*** | -0.064*** | 0.34 |
| Beans | Vegetables | 4.828*** | 0.289*** | -0.126*** | 0.19 |
| Lemon | Vegetables | -0.969*** | 0.371*** | -0.044*** | 0.27 |
| Banana | Fresh Fruits | -1.061 | 0.419*** | -0.059*** | 0.23 |
| Coconut | Fresh Fruits | -1.828*** | 0.276** | -0.079 | 0.62 |
| Green Coconut | Fresh Fruits | -0.174 | 0.221 | -0.104 | 0.16 |
| Orange | Fresh Fruits | -0.538*** | 0.355*** | -0.027*** | 0.27 |
| Papaya | Fresh Fruits | 4.904*** | 0.298*** | -0.087*** | 0.19 |
| Pears | Fresh Fruits | 4.293*** | 0.345*** | -0.194*** | 0.4 |
| Lichi | Fresh Fruits | 2.868 | 0.467* | -0.042*** | 0.25 |
| Apple | Fresh Fruits | 3.554*** | 0.454*** | 0.017*** | 0.32 |
| Grapes | Fresh Fruits | 3.154*** | 0.381*** | -0.004*** | 0.27 |
| Coconut | Dry Fruits | 1.371* | 0.412 | -0.1*** | 0.23 |
| Dates | Dry Fruits | 3.578*** | 0.282 | -0.141*** | 0.16 |
| Cashewnut | Dry Fruits | 0.263*** | 0.501*** | 0.052*** | 0.35 |
| Other Nuts | Dry Fruits | 1.181*** | 0.43 | -0.099*** | 0.31 |
| Raisins | Dry Fruits | 0.676*** | 0.419*** | 0.03*** | 0.3 |
| Other Dry Fruits | Dry Fruits | 2.619*** | 0.338*** | -0.036*** | 0.32 |
| Ginger | Spices | 1.794 | 0.392*** | -0.084*** | 0.29 |
| Cumin | Spices | 1.235*** | 0.34*** | -0.07*** | 0.19 |
| Black Pepper | Spices | 1.39*** | 0.287*** | -0.057*** | 0.12 |
| Tamarind | Spices | 2.398 | 0.318*** | -0.131*** | 0.36 |
| Curry Powder | Spices | 2.114 | 0.274*** | -0.04*** | 0.16 |
| Tea (cups) | Beverages | -0.255 | 0.289*** | 0.176*** | 0.14 |
| Coffee (cups) | Beverages | 0.296 | 0.253 | 0.028 | 0.12 |
| Coffee (powder) | Beverages | 1.313*** | 0.268 | -0.098 | 0.24 |
| Mineral Water | Beverages | 1.327 | 0.663 | -0.015 | 0.52 |
| Cold Beverage | Beverages | 2.543*** | 0.537*** | -0.016*** | 0.31 |
| Fruit Juice | Beverages | 2.893 | 0.456** | 0.01 | 0.21 |
| Cooked Meals | FAFH | 3.405 | -0.123*** | 0.31*** | 0.07 |
| Cooked Meals Free | FAFH | 5.101 | -0.256*** | 0.36*** | 0.18 |
| Snacks | Packaged/Processed Food | 1.15 | 0.459*** | 0.001*** | 0.22 |
| Chips | Packaged/Processed Food | 1.124 | 0.393*** | 0.076*** | 0.31 |
| Pickles | Packaged/Processed Food | 2.782*** | 0.344*** | -0.07*** | 0.32 |
| Sauce | Packaged/Processed Food | 1.777 | 0.399 | 0.053 | 0.21 |
| Note:  ***p<0.01; **p<0.05; *p<0.1  Robust Standard Errors | | | | | |

Table S3: Regression estimates for (log) household consumption of 19 food commodities with (log) household income, urban non-metropolitan, and urban metropolitan (relative to rural) variables as covariates.

| Food Item | Food Item Type | Intercept | Income | Non-Metropolitan | Metropolitan | R^2^ |
| --- | --- | --- | --- | --- | --- | --- |
| Finely-milled Wheat | Cereals | 4.498*** | 0.27*** | -0.066*** | -0.081*** | 0.05 |
| Pigeon Peas | Pulses | 4.749*** | 0.279*** | -0.056*** | -0.173*** | 0.06 |
| Other Oil | Edible Oil | 5.889*** | 0.15*** | 0.027 | 0.136*** | 0.01 |
| Eggs | Eggs, Fish, & Meat | -1.48*** | 0.459*** | -0.007 | 0.039*** | 0.21 |
| Mutton | Eggs, Fish, & Meat | 2.417*** | 0.507*** | -0.104*** | -0.163*** | 0.19 |
| Tomato | Vegetables | 4.467*** | 0.349*** | -0.029*** | -0.012* | 0.1 |
| Carrot | Vegetables | 3.61*** | 0.361*** | -0.043*** | -0.112*** | 0.09 |
| Okra | Vegetables | 4.343*** | 0.301*** | -0.083*** | -0.188*** | 0.07 |
| Lemon | Vegetables | -1.584*** | 0.401*** | -0.023*** | -0.1*** | 0.13 |
| Banana | Fresh Fruits | -0.802*** | 0.419*** | -0.056*** | -0.068*** | 0.17 |
| Green Coconut | Fresh Fruits | -0.792*** | 0.252*** | -0.076*** | -0.191*** | 0.06 |
| Orange | Fresh Fruits | -1.03*** | 0.376*** | -0.064*** | -0.099*** | 0.12 |
| Dates | Dry Fruits | 2.938*** | 0.318*** | -0.081*** | -0.199*** | 0.06 |
| Black Pepper | Spices | 1.439*** | 0.269*** | -0.042*** | -0.042*** | 0.06 |
| Curry Powder | Spices | 1.683*** | 0.3*** | -0.07*** | 0.085*** | 0.07 |
| Coffee (cups) | Beverages | 0.504 | 0.19*** | -0.118* | 0.123* | 0.04 |
| Coffee (powder) | Beverages | 3.63*** | 0.118*** | -0.23*** | -0.223*** | 0.02 |
| Cooked Meals | FAFH | 3.328*** | -0.124*** | 0.233*** | 0.484*** | 0.03 |
| Chips | Packaged/Processed Food | 0.731*** | 0.451*** | 0.003 | 0.125*** | 0.1 |
| Note:  ***p<0.01; **p<0.05; *p<0.1  Robust Standard Errors | | | | | | |

Table S4: Regression estimates for (log) household consumption of 19 food commodities with (log) household income, urban non-metropolitan, urban metropolitan (relative to rural), and state dummy variables as covariates.

| Food Item | Food Item Type | Intercept | Income | Non-Metropolitan | Metropolitan | R^2^ |
| --- | --- | --- | --- | --- | --- | --- |
| Finely-milled Wheat | Cereals | 4.527*** | 0.296*** | -0.055*** | -0.068*** | 0.2 |
| Pigeon Peas | Pulses | 3.25*** | 0.386** | -0.061 | -0.102 | 0.32 |
| Other Oil | Edible Oil | 4.382 | 0.241 | 0.011*** | 0.021* | 0.55 |
| Eggs | Eggs, Fish, & Meat | -1.461*** | 0.461*** | -0.013** | 0.046*** | 0.25 |
| Mutton | Eggs, Fish, & Meat | 2.776*** | 0.504*** | -0.085*** | -0.115*** | 0.26 |
| Tomato | Vegetables | 3.917*** | 0.41*** | -0.039*** | -0.001 | 0.26 |
| Carrot | Vegetables | 4.385*** | 0.336*** | -0.047*** | 0.004 | 0.34 |
| Okra | Vegetables | 4.588*** | 0.33*** | -0.104*** | -0.059*** | 0.24 |
| Lemon | Vegetables | -1.008*** | 0.374*** | -0.019*** | -0.087*** | 0.27 |
| Banana | Fresh Fruits | -1.067*** | 0.419*** | -0.055*** | -0.067*** | 0.23 |
| Green Coconut | Fresh Fruits | -0.205 | 0.227 | -0.056 | -0.159 | 0.16 |
| Orange | Fresh Fruits | -0.517*** | 0.353*** | -0.045*** | 0.003 | 0.27 |
| Dates | Dry Fruits | 3.53*** | 0.287 | -0.116*** | -0.175*** | 0.16 |
| Black Pepper | Spices | 1.389*** | 0.287*** | -0.056*** | -0.059*** | 0.12 |
| Curry Powder | Spices | 2.159*** | 0.271*** | -0.064*** | 0.002 | 0.16 |
| Coffee (cups) | Beverages | 0.398 | 0.244 | -0.042 | 0.097 | 0.12 |
| Coffee (powder) | Beverages | 1.305*** | 0.268 | -0.094 | -0.103 | 0.24 |
| Cooked Meals | FAFH | 3.459*** | -0.127*** | 0.258*** | 0.383*** | 0.08 |
| Chips | Packaged/Processed Food | 1.127*** | 0.393*** | 0.075*** | 0.079*** | 0.31 |
| Note:  ***p<0.01; **p<0.05; *p<0.1  Robust Standard Errors | | | | | | |

Table S5: Multi-level model estimates for (log) household consumption of *Other rice* with household- and district-level covariates.

|  | **Other Rice** | | **Other Rice** | | **Other Rice** | | **Other Rice** | |
| --- | --- | --- | --- | --- | --- | --- | --- | --- |
| *Predictors* | *Estimates* | *CI* | *Estimates* | *CI* | *Estimates* | *CI* | *Estimates* | *CI* |
| Intercept | 7.43 ^***^ | 7.15 – 7.71 | 4.90 ^***^ | 4.43 – 5.37 | 5.11 ^***^ | 4.63 – 5.60 | 5.14 ^***^ | 3.14 – 7.15 |
| Income |  |  | 0.28 ^***^ | 0.24 – 0.32 | 0.20 ^***^ | 0.16 – 0.25 | 0.20 ^***^ | 0.16 – 0.25 |
| Urban |  |  | -0.04 | -0.09 – 0.02 | -0.01 | -0.07 – 0.04 | -0.01 | -0.07 – 0.04 |
| Household Size |  |  |  |  | 0.35 ^***^ | 0.27 – 0.44 | 0.35 ^***^ | 0.27 – 0.43 |
| Household Structure |  |  |  |  | -0.03 | -0.08 – 0.02 | -0.03 | -0.08 – 0.02 |
| Cooking Facility |  |  |  |  | 0.06 | -0.00 – 0.12 | 0.06 | -0.00 – 0.12 |
| Infrastructure |  |  |  |  |  |  | -0.08 | -0.24 – 0.08 |
| Travel time to Cities |  |  |  |  |  |  | 0.08 | -0.17 – 0.33 |
| % Working Women in Urban Areas |  |  |  |  |  |  | 1.91 | -4.04 – 7.86 |
| **Random Effects** | | | | | | | | |
| σ^2^ | 0.54 | | 0.51 | | 0.50 | | 0.50 | |
| (τ_2_)^2^ | 0.46 _District:State_ | | 0.47 _District:State_ | | 0.47 _District:State_ | | 0.48 _District:State_ | |
| (τ_1_)^2^ | 0.52 _State_ | | 0.55 _State_ | | 0.57 _State_ | | 0.52 _State_ | |
| ICC | 0.64 | | 0.67 | | 0.68 | | 0.67 | |
| N | 47 _District_ | | 47 _District_ | | 47 _District_ | | 47 _District_ | |
|  | 33 _State_ | | 33 _State_ | | 33 _State_ | | 33 _State_ | |
| Observations | 3567 | | 3567 | | 3567 | | 3567 | |
| Marginal R^2^ / Conditional R^2^ | 0.000 / 0.645 | | 0.020 / 0.674 | | 0.029 / 0.687 | | 0.036 / 0.681 | |
| ** p<0.05   ** p<0.01   *** p<0.001* | | | | | | | | |

Table S6: Multi-level model estimates for (log) household consumption of *Finely-milled wheat* with household- and district-level covariates.

|  | **Finely-milled Wheat** | | **Finely-milled Wheat** | | **Finely-milled Wheat** | | **Finely-milled Wheat** | |
| --- | --- | --- | --- | --- | --- | --- | --- | --- |
| *Predictors* | *Estimates* | *CI* | *Estimates* | *CI* | *Estimates* | *CI* | *Estimates* | *CI* |
| Intercept | 6.93 ^***^ | 6.81 – 7.04 | 4.12 ^***^ | 3.92 – 4.32 | 4.43 ^***^ | 4.22 – 4.63 | 4.36 ^***^ | 3.77 – 4.95 |
| Income |  |  | 0.31 ^***^ | 0.29 – 0.33 | 0.23 ^***^ | 0.21 – 0.25 | 0.23 ^***^ | 0.21 – 0.25 |
| Urban |  |  | -0.05 ^***^ | -0.08 – -0.03 | -0.00 | -0.03 – 0.02 | -0.00 | -0.03 – 0.02 |
| Household Size |  |  |  |  | 0.36 ^***^ | 0.33 – 0.39 | 0.36 ^***^ | 0.33 – 0.39 |
| Household Structure |  |  |  |  | -0.04 ^***^ | -0.07 – -0.02 | -0.04 ^***^ | -0.07 – -0.02 |
| Cooking Facility |  |  |  |  | -0.02 | -0.04 – 0.01 | -0.02 | -0.04 – 0.01 |
| Infrastructure |  |  |  |  |  |  | -0.04 | -0.09 – 0.00 |
| Travel time to Cities |  |  |  |  |  |  | 0.05 | -0.02 – 0.13 |
| % Working Women in Urban Areas |  |  |  |  |  |  | 2.02 ^*^ | 0.02 – 4.01 |
| **Random Effects** | | | | | | | | |
| σ^2^ | 0.40 | | 0.37 | | 0.36 | | 0.36 | |
| (τ_2_)^2^ | 0.07 _District:State_ | | 0.07 _District:State_ | | 0.07 _District:State_ | | 0.08 _District:State_ | |
| (τ_1_)^2^ | 0.11 _State_ | | 0.11 _State_ | | 0.11 _State_ | | 0.07 _State_ | |
| ICC | 0.30 | | 0.33 | | 0.34 | | 0.29 | |
| N | 71 _District_ | | 71 _District_ | | 71 _District_ | | 71 _District_ | |
|  | 35 _State_ | | 35 _State_ | | 35 _State_ | | 35 _State_ | |
| Observations | 15064 | | 15064 | | 15064 | | 15064 | |
| Marginal R^2^ / Conditional R^2^ | 0.000 / 0.304 | | 0.058 / 0.367 | | 0.086 / 0.393 | | 0.097 / 0.356 | |
| ** p<0.05   ** p<0.01   *** p<0.001* | | | | | | | | |

Table S7: Multi-level model estimates for (log) household consumption of *Semolina* with household- and district-level covariates.

|  | **Semolina** | | **Semolina** | | **Semolina** | | **Semolina** | |
| --- | --- | --- | --- | --- | --- | --- | --- | --- |
| *Predictors* | *Estimates* | *CI* | *Estimates* | *CI* | *Estimates* | *CI* | *Estimates* | *CI* |
| Intercept | 6.67 ^***^ | 6.56 – 6.78 | 3.55 ^***^ | 3.40 – 3.70 | 3.90 ^***^ | 3.74 – 4.05 | 3.94 ^***^ | 3.51 – 4.37 |
| Income |  |  | 0.35 ^***^ | 0.33 – 0.36 | 0.25 ^***^ | 0.24 – 0.26 | 0.25 ^***^ | 0.24 – 0.26 |
| Urban |  |  | -0.04 ^***^ | -0.05 – -0.03 | 0.01 | -0.01 – 0.02 | 0.01 | -0.01 – 0.02 |
| Household Size |  |  |  |  | 0.36 ^***^ | 0.34 – 0.38 | 0.36 ^***^ | 0.34 – 0.38 |
| Household Structure |  |  |  |  | -0.02 ^*^ | -0.03 – -0.00 | -0.02 ^*^ | -0.03 – -0.00 |
| Cooking Facility |  |  |  |  | 0.00 | -0.01 – 0.02 | 0.00 | -0.01 – 0.02 |
| Infrastructure |  |  |  |  |  |  | -0.04 ^*^ | -0.07 – -0.01 |
| Travel time to Cities |  |  |  |  |  |  | 0.04 | -0.02 – 0.09 |
| % Working Women in Urban Areas |  |  |  |  |  |  | 0.60 | -0.87 – 2.07 |
| **Random Effects** | | | | | | | | |
| σ^2^ | 0.35 | | 0.31 | | 0.29 | | 0.29 | |
| (τ_2_)^2^ | 0.04 _District:State_ | | 0.05 _District:State_ | | 0.04 _District:State_ | | 0.04 _District:State_ | |
| (τ_1_)^2^ | 0.10 _State_ | | 0.11 _State_ | | 0.11 _State_ | | 0.09 _State_ | |
| ICC | 0.30 | | 0.33 | | 0.34 | | 0.32 | |
| N | 71 _District_ | | 71 _District_ | | 71 _District_ | | 71 _District_ | |
|  | 35 _State_ | | 35 _State_ | | 35 _State_ | | 35 _State_ | |
| Observations | 33782 | | 33782 | | 33782 | | 33782 | |
| Marginal R^2^ / Conditional R^2^ | 0.000 / 0.300 | | 0.087 / 0.388 | | 0.119 / 0.420 | | 0.125 / 0.402 | |
| ** p<0.05   ** p<0.01   *** p<0.001* | | | | | | | | |

Table S8: Multi-level model estimates for (log) household consumption of *Noodles* with household- and district-level covariates.

|  | **Noodles** | | **Noodles** | | **Noodles** | | **Noodles** | |
| --- | --- | --- | --- | --- | --- | --- | --- | --- |
| *Predictors* | *Estimates* | *CI* | *Estimates* | *CI* | *Estimates* | *CI* | *Estimates* | *CI* |
| Intercept | 6.22 ^***^ | 6.14 – 6.31 | 2.78 ^***^ | 2.57 – 3.00 | 3.00 ^***^ | 2.77 – 3.22 | 3.12 ^***^ | 2.53 – 3.71 |
| Income |  |  | 0.38 ^***^ | 0.35 – 0.40 | 0.32 ^***^ | 0.30 – 0.34 | 0.32 ^***^ | 0.30 – 0.34 |
| Urban |  |  | -0.02 | -0.05 – 0.01 | 0.02 | -0.01 – 0.05 | 0.02 | -0.01 – 0.05 |
| Household Size |  |  |  |  | 0.29 ^***^ | 0.25 – 0.33 | 0.29 ^***^ | 0.25 – 0.33 |
| Household Structure |  |  |  |  | -0.06 ^***^ | -0.09 – -0.03 | -0.06 ^***^ | -0.09 – -0.03 |
| Cooking Facility |  |  |  |  | -0.01 | -0.04 – 0.02 | -0.01 | -0.04 – 0.02 |
| Infrastructure |  |  |  |  |  |  | -0.05 ^*^ | -0.09 – -0.00 |
| Travel time to Cities |  |  |  |  |  |  | 0.02 | -0.05 – 0.09 |
| % Working Women in Urban Areas |  |  |  |  |  |  | 2.29 ^*^ | 0.33 – 4.24 |
| **Random Effects** | | | | | | | | |
| σ^2^ | 0.52 | | 0.47 | | 0.46 | | 0.46 | |
| (τ_2_)^2^ | 0.08 _District:State_ | | 0.08 _District:State_ | | 0.08 _District:State_ | | 0.08 _District:State_ | |
| (τ_1_)^2^ | 0.05 _State_ | | 0.06 _State_ | | 0.06 _State_ | | 0.04 _State_ | |
| ICC | 0.21 | | 0.23 | | 0.23 | | 0.21 | |
| N | 70 _District_ | | 70 _District_ | | 70 _District_ | | 70 _District_ | |
|  | 35 _State_ | | 35 _State_ | | 35 _State_ | | 35 _State_ | |
| Observations | 13331 | | 13331 | | 13331 | | 13331 | |
| Marginal R^2^ / Conditional R^2^ | 0.000 / 0.209 | | 0.079 / 0.290 | | 0.091 / 0.299 | | 0.097 / 0.283 | |
| ** p<0.05   ** p<0.01   *** p<0.001* | | | | | | | | |

Table S9: Multi-level model estimates for (log) household consumption of *Bread* with household- and district-level covariates.

|  | **Bread** | | **Bread** | | **Bread** | | **Bread** | |
| --- | --- | --- | --- | --- | --- | --- | --- | --- |
| *Predictors* | *Estimates* | *CI* | *Estimates* | *CI* | *Estimates* | *CI* | *Estimates* | *CI* |
| Intercept | 6.75 ^***^ | 6.59 – 6.90 | 3.07 ^***^ | 2.87 – 3.27 | 3.38 ^***^ | 3.18 – 3.58 | 3.69 ^***^ | 3.08 – 4.29 |
| Income |  |  | 0.40 ^***^ | 0.38 – 0.41 | 0.33 ^***^ | 0.31 – 0.35 | 0.33 ^***^ | 0.31 – 0.35 |
| Urban |  |  | 0.05 ^***^ | 0.04 – 0.07 | 0.06 ^***^ | 0.04 – 0.08 | 0.06 ^***^ | 0.04 – 0.08 |
| Household Size |  |  |  |  | 0.25 ^***^ | 0.23 – 0.28 | 0.26 ^***^ | 0.23 – 0.28 |
| Household Structure |  |  |  |  | -0.04 ^***^ | -0.06 – -0.02 | -0.04 ^***^ | -0.07 – -0.02 |
| Cooking Facility |  |  |  |  | 0.06 ^***^ | 0.04 – 0.09 | 0.06 ^***^ | 0.04 – 0.09 |
| Infrastructure |  |  |  |  |  |  | -0.03 | -0.07 – 0.02 |
| Travel time to Cities |  |  |  |  |  |  | -0.05 | -0.12 – 0.03 |
| % Working Women in Urban Areas |  |  |  |  |  |  | 3.20 ^**^ | 1.13 – 5.26 |
| **Random Effects** | | | | | | | | |
| σ^2^ | 0.50 | | 0.45 | | 0.44 | | 0.44 | |
| (τ_2_)^2^ | 0.10 _District:State_ | | 0.09 _District:State_ | | 0.09 _District:State_ | | 0.08 _District:State_ | |
| (τ_1_)^2^ | 0.19 _State_ | | 0.17 _State_ | | 0.16 _State_ | | 0.16 _State_ | |
| ICC | 0.37 | | 0.37 | | 0.36 | | 0.35 | |
| N | 71 _District_ | | 71 _District_ | | 71 _District_ | | 71 _District_ | |
|  | 35 _State_ | | 35 _State_ | | 35 _State_ | | 35 _State_ | |
| Observations | 24476 | | 24476 | | 24476 | | 24476 | |
| Marginal R^2^ / Conditional R^2^ | 0.000 / 0.369 | | 0.083 / 0.419 | | 0.090 / 0.420 | | 0.110 / 0.424 | |
| ** p<0.05   ** p<0.01   *** p<0.001* | | | | | | | | |

Table S10: Multi-level model estimates for (log) household consumption of *Other wheat* with household- and district-level covariates.

|  | **Other Wheat** | | **Other Wheat** | | **Other Wheat** | | **Other Wheat** | |
| --- | --- | --- | --- | --- | --- | --- | --- | --- |
| *Predictors* | *Estimates* | *CI* | *Estimates* | *CI* | *Estimates* | *CI* | *Estimates* | *CI* |
| Intercept | 7.02 ^***^ | 6.82 – 7.21 | 5.38 ^***^ | 4.51 – 6.25 | 5.63 ^***^ | 4.73 – 6.53 | 6.36 ^***^ | 4.26 – 8.45 |
| Income |  |  | 0.18 ^***^ | 0.09 – 0.28 | 0.14 ^**^ | 0.04 – 0.25 | 0.14 ^**^ | 0.04 – 0.25 |
| Urban |  |  | -0.06 | -0.18 – 0.07 | 0.03 | -0.11 – 0.17 | 0.03 | -0.11 – 0.17 |
| Household Size |  |  |  |  | 0.33 ^***^ | 0.16 – 0.50 | 0.33 ^***^ | 0.16 – 0.50 |
| Household Structure |  |  |  |  | -0.14 ^*^ | -0.25 – -0.02 | -0.14 ^*^ | -0.25 – -0.02 |
| Cooking Facility |  |  |  |  | -0.08 | -0.23 – 0.06 | -0.08 | -0.22 – 0.06 |
| Infrastructure |  |  |  |  |  |  | -0.08 | -0.24 – 0.08 |
| Travel time to Cities |  |  |  |  |  |  | -0.07 | -0.29 – 0.16 |
| % Working Women in Urban Areas |  |  |  |  |  |  | 1.75 | -4.16 – 7.65 |
| **Random Effects** | | | | | | | | |
| σ^2^ | 0.52 | | 0.51 | | 0.50 | | 0.50 | |
| (τ_2_)^2^ | 0.42 _District:State_ | | 0.44 _District:State_ | | 0.44 _District:State_ | | 0.45 _District:State_ | |
| (τ_1_)^2^ | 0.19 _State_ | | 0.19 _State_ | | 0.18 _State_ | | 0.17 _State_ | |
| ICC | 0.54 | | 0.55 | | 0.56 | | 0.56 | |
| N | 46 _District_ | | 46 _District_ | | 46 _District_ | | 46 _District_ | |
|  | 32 _State_ | | 32 _State_ | | 32 _State_ | | 32 _State_ | |
| Observations | 813 | | 813 | | 813 | | 813 | |
| Marginal R^2^ / Conditional R^2^ | 0.000 / 0.540 | | 0.012 / 0.560 | | 0.025 / 0.567 | | 0.029 / 0.569 | |
| ** p<0.05   ** p<0.01   *** p<0.001* | | | | | | | | |

Table S11: Multi-level model estimates for (log) household consumption of *Pigeon Peas* with household- and district-level covariates.

|  | **Pigeon Peas** | | **Pigeon Peas** | | **Pigeon Peas** | | **Pigeon Peas** | |
| --- | --- | --- | --- | --- | --- | --- | --- | --- |
| *Predictors* | *Estimates* | *CI* | *Estimates* | *CI* | *Estimates* | *CI* | *Estimates* | *CI* |
| Intercept | 6.96 ^***^ | 6.82 – 7.09 | 3.50 ^***^ | 3.33 – 3.67 | 4.05 ^***^ | 3.88 – 4.21 | 4.03 ^***^ | 3.42 – 4.65 |
| Income |  |  | 0.39 ^***^ | 0.38 – 0.40 | 0.25 ^***^ | 0.24 – 0.26 | 0.25 ^***^ | 0.24 – 0.26 |
| Urban |  |  | -0.04 ^***^ | -0.05 – -0.03 | 0.00 | -0.01 – 0.01 | 0.00 | -0.01 – 0.01 |
| Household Size |  |  |  |  | 0.46 ^***^ | 0.45 – 0.48 | 0.46 ^***^ | 0.45 – 0.48 |
| Household Structure |  |  |  |  | -0.02 ^**^ | -0.02 – -0.01 | -0.02 ^**^ | -0.02 – -0.01 |
| Cooking Facility |  |  |  |  | 0.03 ^***^ | 0.02 – 0.04 | 0.03 ^***^ | 0.02 – 0.04 |
| Infrastructure |  |  |  |  |  |  | -0.00 | -0.05 – 0.04 |
| Travel time to Cities |  |  |  |  |  |  | 0.01 | -0.07 – 0.10 |
| % Working Women in Urban Areas |  |  |  |  |  |  | -1.38 | -3.62 – 0.86 |
| **Random Effects** | | | | | | | | |
| σ^2^ | 0.34 | | 0.29 | | 0.26 | | 0.26 | |
| (τ_2_)^2^ | 0.10 _District:State_ | | 0.11 _District:State_ | | 0.11 _District:State_ | | 0.11 _District:State_ | |
| (τ_1_)^2^ | 0.14 _State_ | | 0.19 _State_ | | 0.18 _State_ | | 0.18 _State_ | |
| ICC | 0.41 | | 0.51 | | 0.52 | | 0.52 | |
| N | 71 _District_ | | 71 _District_ | | 71 _District_ | | 71 _District_ | |
|  | 34 _State_ | | 34 _State_ | | 34 _State_ | | 34 _State_ | |
| Observations | 56392 | | 56392 | | 56392 | | 56392 | |
| Marginal R^2^ / Conditional R^2^ | 0.000 / 0.414 | | 0.090 / 0.555 | | 0.134 / 0.588 | | 0.136 / 0.588 | |
| ** p<0.05   ** p<0.01   *** p<0.001* | | | | | | | | |

Table S12: Multi-level model estimates for (log) household consumption of *Green Gram* with household- and district-level covariates.

|  | **Green Gram** | | **Green Gram** | | **Green Gram** | | **Green Gram** | |
| --- | --- | --- | --- | --- | --- | --- | --- | --- |
| *Predictors* | *Estimates* | *CI* | *Estimates* | *CI* | *Estimates* | *CI* | *Estimates* | *CI* |
| Intercept | 6.65 ^***^ | 6.55 – 6.76 | 3.92 ^***^ | 3.79 – 4.05 | 4.32 ^***^ | 4.19 – 4.44 | 4.44 ^***^ | 3.98 – 4.90 |
| Income |  |  | 0.31 ^***^ | 0.30 – 0.32 | 0.21 ^***^ | 0.20 – 0.22 | 0.21 ^***^ | 0.20 – 0.22 |
| Urban |  |  | -0.12 ^***^ | -0.13 – -0.10 | -0.05 ^***^ | -0.07 – -0.04 | -0.05 ^***^ | -0.07 – -0.04 |
| Household Size |  |  |  |  | 0.42 ^***^ | 0.40 – 0.44 | 0.42 ^***^ | 0.40 – 0.44 |
| Household Structure |  |  |  |  | -0.04 ^***^ | -0.05 – -0.03 | -0.04 ^***^ | -0.05 – -0.03 |
| Cooking Facility |  |  |  |  | -0.03 ^***^ | -0.04 – -0.02 | -0.03 ^***^ | -0.04 – -0.02 |
| Infrastructure |  |  |  |  |  |  | -0.04 ^*^ | -0.08 – -0.01 |
| Travel time to Cities |  |  |  |  |  |  | 0.02 | -0.04 – 0.08 |
| % Working Women in Urban Areas |  |  |  |  |  |  | 1.60 | -0.01 – 3.21 |
| **Random Effects** | | | | | | | | |
| σ^2^ | 0.38 | | 0.34 | | 0.32 | | 0.32 | |
| (τ_2_)^2^ | 0.06 _District:State_ | | 0.07 _District:State_ | | 0.06 _District:State_ | | 0.06 _District:State_ | |
| (τ_1_)^2^ | 0.08 _State_ | | 0.07 _State_ | | 0.07 _State_ | | 0.05 _State_ | |
| ICC | 0.28 | | 0.29 | | 0.29 | | 0.26 | |
| N | 69 _District_ | | 69 _District_ | | 69 _District_ | | 69 _District_ | |
|  | 35 _State_ | | 35 _State_ | | 35 _State_ | | 35 _State_ | |
| Observations | 49790 | | 49790 | | 49790 | | 49790 | |
| Marginal R^2^ / Conditional R^2^ | 0.000 / 0.284 | | 0.068 / 0.336 | | 0.113 / 0.368 | | 0.119 / 0.345 | |
| ** p<0.05   ** p<0.01   *** p<0.001* | | | | | | | | |

Table S13: Multi-level model estimates for (log) household consumption of *Gram Products* with household- and district-level covariates.

|  | **Gram Products** | | **Gram Products** | | **Gram Products** | | **Gram Products** | |
| --- | --- | --- | --- | --- | --- | --- | --- | --- |
| *Predictors* | *Estimates* | *CI* | *Estimates* | *CI* | *Estimates* | *CI* | *Estimates* | *CI* |
| Intercept | 6.40 ^***^ | 6.27 – 6.52 | 3.11 ^***^ | 2.86 – 3.36 | 3.54 ^***^ | 3.29 – 3.79 | 4.05 ^***^ | 3.14 – 4.96 |
| Income |  |  | 0.37 ^***^ | 0.34 – 0.39 | 0.26 ^***^ | 0.23 – 0.29 | 0.26 ^***^ | 0.23 – 0.29 |
| Urban |  |  | -0.06 ^***^ | -0.09 – -0.03 | -0.05 ^**^ | -0.08 – -0.02 | -0.04 ^**^ | -0.07 – -0.01 |
| Household Size |  |  |  |  | 0.33 ^***^ | 0.29 – 0.38 | 0.33 ^***^ | 0.29 – 0.37 |
| Household Structure |  |  |  |  | 0.00 | -0.03 – 0.03 | 0.00 | -0.03 – 0.03 |
| Cooking Facility |  |  |  |  | 0.09 ^***^ | 0.06 – 0.12 | 0.09 ^***^ | 0.06 – 0.12 |
| Infrastructure |  |  |  |  |  |  | -0.11 ^**^ | -0.19 – -0.04 |
| Travel time to Cities |  |  |  |  |  |  | 0.03 | -0.09 – 0.14 |
| % Working Women in Urban Areas |  |  |  |  |  |  | 1.04 | -1.81 – 3.88 |
| **Random Effects** | | | | | | | | |
| σ^2^ | 0.35 | | 0.31 | | 0.30 | | 0.30 | |
| (τ_2_)^2^ | 0.13 _District:State_ | | 0.14 _District:State_ | | 0.14 _District:State_ | | 0.14 _District:State_ | |
| (τ_1_)^2^ | 0.09 _State_ | | 0.09 _State_ | | 0.07 _State_ | | 0.03 _State_ | |
| ICC | 0.39 | | 0.42 | | 0.42 | | 0.36 | |
| N | 46 _District_ | | 46 _District_ | | 46 _District_ | | 46 _District_ | |
|  | 33 _State_ | | 33 _State_ | | 33 _State_ | | 33 _State_ | |
| Observations | 7112 | | 7112 | | 7112 | | 7112 | |
| Marginal R^2^ / Conditional R^2^ | 0.000 / 0.387 | | 0.082 / 0.466 | | 0.105 / 0.482 | | 0.126 / 0.445 | |
| ** p<0.05   ** p<0.01   *** p<0.001* | | | | | | | | |

Table S14: Multi-level model estimates for (log) household consumption of *Gram Flour* with household- and district-level covariates.

|  | **Gram Flour** | | **Gram Flour** | | **Gram Flour** | | **Gram Flour** | |
| --- | --- | --- | --- | --- | --- | --- | --- | --- |
| *Predictors* | *Estimates* | *CI* | *Estimates* | *CI* | *Estimates* | *CI* | *Estimates* | *CI* |
| Intercept | 6.17 ^***^ | 6.07 – 6.26 | 2.75 ^***^ | 2.61 – 2.89 | 3.11 ^***^ | 2.98 – 3.25 | 2.93 ^***^ | 2.48 – 3.39 |
| Income |  |  | 0.38 ^***^ | 0.37 – 0.39 | 0.29 ^***^ | 0.28 – 0.30 | 0.29 ^***^ | 0.28 – 0.30 |
| Urban |  |  | -0.07 ^***^ | -0.08 – -0.06 | -0.01 | -0.03 – 0.00 | -0.01 | -0.03 – 0.00 |
| Household Size |  |  |  |  | 0.36 ^***^ | 0.34 – 0.38 | 0.36 ^***^ | 0.34 – 0.38 |
| Household Structure |  |  |  |  | -0.03 ^***^ | -0.05 – -0.02 | -0.03 ^***^ | -0.05 – -0.02 |
| Cooking Facility |  |  |  |  | -0.03 ^***^ | -0.05 – -0.02 | -0.03 ^***^ | -0.05 – -0.02 |
| Infrastructure |  |  |  |  |  |  | 0.00 | -0.03 – 0.03 |
| Travel time to Cities |  |  |  |  |  |  | 0.03 | -0.03 – 0.09 |
| % Working Women in Urban Areas |  |  |  |  |  |  | 1.18 | -0.47 – 2.83 |
| **Random Effects** | | | | | | | | |
| σ^2^ | 0.38 | | 0.33 | | 0.31 | | 0.31 | |
| (τ_2_)^2^ | 0.05 _District:State_ | | 0.05 _District:State_ | | 0.05 _District:State_ | | 0.05 _District:State_ | |
| (τ_1_)^2^ | 0.07 _State_ | | 0.08 _State_ | | 0.07 _State_ | | 0.07 _State_ | |
| ICC | 0.23 | | 0.28 | | 0.26 | | 0.26 | |
| N | 71 _District_ | | 71 _District_ | | 71 _District_ | | 71 _District_ | |
|  | 34 _State_ | | 34 _State_ | | 34 _State_ | | 34 _State_ | |
| Observations | 35442 | | 35442 | | 35442 | | 35442 | |
| Marginal R^2^ / Conditional R^2^ | 0.000 / 0.232 | | 0.104 / 0.353 | | 0.138 / 0.364 | | 0.138 / 0.366 | |
| ** p<0.05   ** p<0.01   *** p<0.001* | | | | | | | | |

Table S15: Multi-level model estimates for (log) household consumption of *Baby Food* with household- and district-level covariates.

|  | **Baby Food** | | **Baby Food** | | **Baby Food** | | **Baby Food** | |
| --- | --- | --- | --- | --- | --- | --- | --- | --- |
| *Predictors* | *Estimates* | *CI* | *Estimates* | *CI* | *Estimates* | *CI* | *Estimates* | *CI* |
| Intercept | 6.61 ^***^ | 6.53 – 6.70 | 4.15 ^***^ | 3.69 – 4.62 | 4.05 ^***^ | 3.49 – 4.61 | 3.80 ^***^ | 2.92 – 4.68 |
| Income |  |  | 0.27 ^***^ | 0.22 – 0.32 | 0.29 ^***^ | 0.23 – 0.35 | 0.29 ^***^ | 0.23 – 0.35 |
| Urban |  |  | 0.01 | -0.06 – 0.07 | 0.02 | -0.05 – 0.09 | 0.01 | -0.05 – 0.08 |
| Household Size |  |  |  |  | -0.04 | -0.13 – 0.05 | -0.04 | -0.13 – 0.05 |
| Household Structure |  |  |  |  | -0.01 | -0.13 – 0.10 | -0.01 | -0.13 – 0.10 |
| Cooking Facility |  |  |  |  | -0.05 | -0.13 – 0.02 | -0.06 | -0.13 – 0.02 |
| Infrastructure |  |  |  |  |  |  | 0.01 | -0.05 – 0.06 |
| Travel time to Cities |  |  |  |  |  |  | 0.04 | -0.05 – 0.13 |
| % Working Women in Urban Areas |  |  |  |  |  |  | 1.68 | -0.57 – 3.92 |
| **Random Effects** | | | | | | | | |
| σ^2^ | 0.47 | | 0.45 | | 0.45 | | 0.45 | |
| (τ_2_)^2^ | 0.05 _District:State_ | | 0.04 _District:State_ | | 0.04 _District:State_ | | 0.04 _District:State_ | |
| (τ_1_)^2^ | 0.04 _State_ | | 0.05 _State_ | | 0.05 _State_ | | 0.05 _State_ | |
| ICC | 0.16 | | 0.16 | | 0.16 | | 0.16 | |
| N | 51 _District_ | | 51 _District_ | | 51 _District_ | | 51 _District_ | |
|  | 35 _State_ | | 35 _State_ | | 35 _State_ | | 35 _State_ | |
| Observations | 2161 | | 2161 | | 2161 | | 2161 | |
| Marginal R^2^ / Conditional R^2^ | 0.000 / 0.156 | | 0.052 / 0.208 | | 0.053 / 0.208 | | 0.058 / 0.212 | |
| ** p<0.05   ** p<0.01   *** p<0.001* | | | | | | | | |

Table S16: Multi-level model estimates for (log) household consumption of *Milk Powder* with household- and district-level covariates.

|  | **Milk Powder** | | **Milk Powder** | | **Milk Powder** | | **Milk Powder** | |
| --- | --- | --- | --- | --- | --- | --- | --- | --- |
| *Predictors* | *Estimates* | *CI* | *Estimates* | *CI* | *Estimates* | *CI* | *Estimates* | *CI* |
| Intercept | 6.30 ^***^ | 6.13 – 6.47 | 1.29 ^***^ | 1.01 – 1.57 | 1.73 ^***^ | 1.42 – 2.03 | 1.66 ^***^ | 0.76 – 2.55 |
| Income |  |  | 0.56 ^***^ | 0.53 – 0.58 | 0.48 ^***^ | 0.45 – 0.51 | 0.48 ^***^ | 0.45 – 0.51 |
| Urban |  |  | 0.05 ^***^ | 0.02 – 0.09 | 0.03 ^*^ | 0.00 – 0.07 | 0.03 ^*^ | 0.00 – 0.07 |
| Household Size |  |  |  |  | 0.15 ^***^ | 0.11 – 0.20 | 0.15 ^***^ | 0.11 – 0.20 |
| Household Structure |  |  |  |  | -0.02 | -0.05 – 0.02 | -0.02 | -0.05 – 0.02 |
| Cooking Facility |  |  |  |  | 0.12 ^***^ | 0.09 – 0.16 | 0.12 ^***^ | 0.09 – 0.16 |
| Infrastructure |  |  |  |  |  |  | -0.03 | -0.09 – 0.03 |
| Travel time to Cities |  |  |  |  |  |  | 0.04 | -0.08 – 0.15 |
| % Working Women in Urban Areas |  |  |  |  |  |  | 1.05 | -1.90 – 4.01 |
| **Random Effects** | | | | | | | | |
| σ^2^ | 0.52 | | 0.44 | | 0.43 | | 0.43 | |
| (τ_2_)^2^ | 0.13 _District:State_ | | 0.11 _District:State_ | | 0.11 _District:State_ | | 0.11 _District:State_ | |
| (τ_1_)^2^ | 0.22 _State_ | | 0.16 _State_ | | 0.15 _State_ | | 0.15 _State_ | |
| ICC | 0.40 | | 0.38 | | 0.38 | | 0.37 | |
| N | 54 _District_ | | 54 _District_ | | 54 _District_ | | 54 _District_ | |
|  | 34 _State_ | | 34 _State_ | | 34 _State_ | | 34 _State_ | |
| Observations | 8861 | | 8861 | | 8861 | | 8861 | |
| Marginal R^2^ / Conditional R^2^ | 0.000 / 0.398 | | 0.140 / 0.467 | | 0.145 / 0.471 | | 0.161 / 0.475 | |
| ** p<0.05   ** p<0.01   *** p<0.001* | | | | | | | | |

Table S17: Multi-level model estimates for (log) household consumption of *Curd* with household- and district-level covariates.

|  | **Curd** | | **Curd** | | **Curd** | | **Curd** | |
| --- | --- | --- | --- | --- | --- | --- | --- | --- |
| *Predictors* | *Estimates* | *CI* | *Estimates* | *CI* | *Estimates* | *CI* | *Estimates* | *CI* |
| Intercept | 6.88 ^***^ | 6.71 – 7.06 | 2.59 ^***^ | 2.29 – 2.90 | 2.58 ^***^ | 2.26 – 2.89 | 3.05 ^***^ | 2.19 – 3.91 |
| Income |  |  | 0.48 ^***^ | 0.45 – 0.51 | 0.46 ^***^ | 0.43 – 0.50 | 0.47 ^***^ | 0.44 – 0.50 |
| Urban |  |  | -0.11 ^***^ | -0.15 – -0.08 | -0.09 ^***^ | -0.13 – -0.05 | -0.09 ^***^ | -0.13 – -0.05 |
| Household Size |  |  |  |  | 0.10 ^***^ | 0.05 – 0.15 | 0.10 ^***^ | 0.05 – 0.15 |
| Household Structure |  |  |  |  | 0.00 | -0.03 – 0.04 | 0.00 | -0.03 – 0.04 |
| Cooking Facility |  |  |  |  | -0.04 ^*^ | -0.08 – -0.00 | -0.04 ^*^ | -0.08 – -0.00 |
| Infrastructure |  |  |  |  |  |  | -0.05 | -0.11 – 0.02 |
| Travel time to Cities |  |  |  |  |  |  | -0.03 | -0.14 – 0.07 |
| % Working Women in Urban Areas |  |  |  |  |  |  | -1.43 | -4.23 – 1.37 |
| **Random Effects** | | | | | | | | |
| σ^2^ | 0.67 | | 0.60 | | 0.59 | | 0.59 | |
| (τ_2_)^2^ | 0.12 _District:State_ | | 0.12 _District:State_ | | 0.12 _District:State_ | | 0.11 _District:State_ | |
| (τ_1_)^2^ | 0.22 _State_ | | 0.21 _State_ | | 0.22 _State_ | | 0.22 _State_ | |
| ICC | 0.34 | | 0.36 | | 0.36 | | 0.36 | |
| N | 67 _District_ | | 67 _District_ | | 67 _District_ | | 67 _District_ | |
|  | 34 _State_ | | 34 _State_ | | 34 _State_ | | 34 _State_ | |
| Observations | 10038 | | 10038 | | 10038 | | 10038 | |
| Marginal R^2^ / Conditional R^2^ | 0.000 / 0.336 | | 0.081 / 0.409 | | 0.083 / 0.411 | | 0.083 / 0.411 | |
| ** p<0.05   ** p<0.01   *** p<0.001* | | | | | | | | |

Table S18: Multi-level model estimates for (log) household consumption of *Clarified Butter* with household- and district-level covariates.

|  | **Clarified Butter** | | **Clarified Butter** | | **Clarified Butter** | | **Clarified Butter** | |
| --- | --- | --- | --- | --- | --- | --- | --- | --- |
| *Predictors* | *Estimates* | *CI* | *Estimates* | *CI* | *Estimates* | *CI* | *Estimates* | *CI* |
| Intercept | 5.98 ^***^ | 5.77 – 6.18 | 1.38 ^***^ | 1.13 – 1.64 | 1.62 ^***^ | 1.36 – 1.89 | 1.20 ^***^ | 0.56 – 1.84 |
| Income |  |  | 0.50 ^***^ | 0.48 – 0.51 | 0.43 ^***^ | 0.42 – 0.45 | 0.43 ^***^ | 0.42 – 0.45 |
| Urban |  |  | -0.03 ^**^ | -0.05 – -0.01 | -0.03 ^*^ | -0.05 – -0.01 | -0.03 ^*^ | -0.05 – -0.01 |
| Household Size |  |  |  |  | 0.20 ^***^ | 0.17 – 0.23 | 0.20 ^***^ | 0.17 – 0.23 |
| Household Structure |  |  |  |  | -0.00 | -0.02 – 0.02 | -0.00 | -0.02 – 0.02 |
| Cooking Facility |  |  |  |  | 0.07 ^***^ | 0.04 – 0.09 | 0.07 ^***^ | 0.04 – 0.09 |
| Infrastructure |  |  |  |  |  |  | 0.02 | -0.03 – 0.07 |
| Travel time to Cities |  |  |  |  |  |  | 0.06 | -0.02 – 0.13 |
| % Working Women in Urban Areas |  |  |  |  |  |  | 0.13 | -1.96 – 2.22 |
| **Random Effects** | | | | | | | | |
| σ^2^ | 0.44 | | 0.37 | | 0.36 | | 0.36 | |
| (τ_2_)^2^ | 0.08 _District:State_ | | 0.07 _District:State_ | | 0.07 _District:State_ | | 0.07 _District:State_ | |
| (τ_1_)^2^ | 0.37 _State_ | | 0.38 _State_ | | 0.37 _State_ | | 0.37 _State_ | |
| ICC | 0.50 | | 0.55 | | 0.55 | | 0.55 | |
| N | 71 _District_ | | 71 _District_ | | 71 _District_ | | 71 _District_ | |
|  | 35 _State_ | | 35 _State_ | | 35 _State_ | | 35 _State_ | |
| Observations | 16771 | | 16771 | | 16771 | | 16771 | |
| Marginal R^2^ / Conditional R^2^ | 0.000 / 0.501 | | 0.098 / 0.595 | | 0.102 / 0.597 | | 0.102 / 0.599 | |
| ** p<0.05   ** p<0.01   *** p<0.001* | | | | | | | | |

Table S19: Multi-level model estimates for (log) household consumption of *Butter* with household- and district-level covariates.

|  | **Butter** | | **Butter** | | **Butter** | | **Butter** | |
| --- | --- | --- | --- | --- | --- | --- | --- | --- |
| *Predictors* | *Estimates* | *CI* | *Estimates* | *CI* | *Estimates* | *CI* | *Estimates* | *CI* |
| Intercept | 5.58 ^***^ | 5.44 – 5.72 | 2.34 ^***^ | 1.90 – 2.77 | 2.53 ^***^ | 2.08 – 2.97 | 2.60 ^***^ | 1.55 – 3.66 |
| Income |  |  | 0.34 ^***^ | 0.30 – 0.38 | 0.29 ^***^ | 0.24 – 0.34 | 0.29 ^***^ | 0.25 – 0.34 |
| Urban |  |  | 0.01 | -0.06 – 0.07 | 0.02 | -0.05 – 0.08 | 0.02 | -0.05 – 0.08 |
| Household Size |  |  |  |  | 0.19 ^***^ | 0.11 – 0.27 | 0.19 ^***^ | 0.11 – 0.27 |
| Household Structure |  |  |  |  | -0.02 | -0.07 – 0.03 | -0.02 | -0.07 – 0.03 |
| Cooking Facility |  |  |  |  | 0.05 | -0.02 – 0.13 | 0.05 | -0.03 – 0.13 |
| Infrastructure |  |  |  |  |  |  | -0.04 | -0.11 – 0.04 |
| Travel time to Cities |  |  |  |  |  |  | 0.02 | -0.10 – 0.14 |
| % Working Women in Urban Areas |  |  |  |  |  |  | 0.82 | -2.34 – 3.98 |
| **Random Effects** | | | | | | | | |
| σ^2^ | 0.40 | | 0.37 | | 0.36 | | 0.36 | |
| (τ_2_)^2^ | 0.12 _District:State_ | | 0.13 _District:State_ | | 0.12 _District:State_ | | 0.12 _District:State_ | |
| (τ_1_)^2^ | 0.12 _State_ | | 0.13 _State_ | | 0.12 _State_ | | 0.13 _State_ | |
| ICC | 0.38 | | 0.41 | | 0.40 | | 0.40 | |
| N | 52 _District_ | | 52 _District_ | | 52 _District_ | | 52 _District_ | |
|  | 33 _State_ | | 33 _State_ | | 33 _State_ | | 33 _State_ | |
| Observations | 2865 | | 2865 | | 2865 | | 2865 | |
| Marginal R^2^ / Conditional R^2^ | 0.000 / 0.377 | | 0.062 / 0.444 | | 0.065 / 0.442 | | 0.068 / 0.445 | |
| ** p<0.05   ** p<0.01   *** p<0.001* | | | | | | | | |

Table S20: Multi-level model estimates for (log) household consumption of *Honey* with household- and district-level covariates.

|  | **Honey** | | **Honey** | | **Honey** | | **Honey** | |
| --- | --- | --- | --- | --- | --- | --- | --- | --- |
| *Predictors* | *Estimates* | *CI* | *Estimates* | *CI* | *Estimates* | *CI* | *Estimates* | *CI* |
| Intercept | 5.09 ^***^ | 4.92 – 5.26 | 2.27 ^***^ | 1.62 – 2.91 | 2.48 ^***^ | 1.82 – 3.14 | 2.65 ^***^ | 1.41 – 3.88 |
| Income |  |  | 0.30 ^***^ | 0.24 – 0.37 | 0.30 ^***^ | 0.23 – 0.37 | 0.30 ^***^ | 0.23 – 0.37 |
| Urban |  |  | -0.07 | -0.16 – 0.03 | -0.08 | -0.18 – 0.03 | -0.08 | -0.19 – 0.03 |
| Household Size |  |  |  |  | 0.08 | -0.05 – 0.20 | 0.08 | -0.04 – 0.20 |
| Household Structure |  |  |  |  | -0.12 ^**^ | -0.21 – -0.03 | -0.12 ^**^ | -0.21 – -0.03 |
| Cooking Facility |  |  |  |  | 0.04 | -0.09 – 0.16 | 0.04 | -0.08 – 0.16 |
| Infrastructure |  |  |  |  |  |  | -0.13 ^**^ | -0.22 – -0.04 |
| Travel time to Cities |  |  |  |  |  |  | 0.08 | -0.05 – 0.21 |
| % Working Women in Urban Areas |  |  |  |  |  |  | 6.94 ^***^ | 3.63 – 10.24 |
| **Random Effects** | | | | | | | | |
| σ^2^ | 0.64 | | 0.61 | | 0.61 | | 0.61 | |
| (τ_2_)^2^ | 0.10 _District:State_ | | 0.10 _District:State_ | | 0.10 _District:State_ | | 0.09 _District:State_ | |
| (τ_1_)^2^ | 0.19 _State_ | | 0.17 _State_ | | 0.17 _State_ | | 0.11 _State_ | |
| ICC | 0.31 | | 0.31 | | 0.30 | | 0.25 | |
| N | 54 _District_ | | 54 _District_ | | 54 _District_ | | 54 _District_ | |
|  | 33 _State_ | | 33 _State_ | | 33 _State_ | | 33 _State_ | |
| Observations | 1574 | | 1574 | | 1574 | | 1574 | |
| Marginal R^2^ / Conditional R^2^ | 0.000 / 0.309 | | 0.041 / 0.335 | | 0.045 / 0.336 | | 0.084 / 0.310 | |
| ** p<0.05   ** p<0.01   *** p<0.001* | | | | | | | | |

Table S21: Multi-level model estimates for (log) household consumption of *Groundnut oil* with household- and district-level covariates.

|  | **Groundnut Oil** | | **Groundnut Oil** | | **Groundnut Oil** | | **Groundnut Oil** | |
| --- | --- | --- | --- | --- | --- | --- | --- | --- |
| *Predictors* | *Estimates* | *CI* | *Estimates* | *CI* | *Estimates* | *CI* | *Estimates* | *CI* |
| Intercept | 7.58 ^***^ | 7.41 – 7.75 | 4.32 ^***^ | 4.05 – 4.60 | 4.94 ^***^ | 4.66 – 5.22 | 4.24 ^***^ | 3.18 – 5.31 |
| Income |  |  | 0.37 ^***^ | 0.34 – 0.39 | 0.22 ^***^ | 0.19 – 0.25 | 0.22 ^***^ | 0.19 – 0.25 |
| Urban |  |  | -0.07 ^***^ | -0.10 – -0.03 | -0.02 | -0.05 – 0.01 | -0.02 | -0.05 – 0.01 |
| Household Size |  |  |  |  | 0.45 ^***^ | 0.41 – 0.50 | 0.45 ^***^ | 0.41 – 0.50 |
| Household Structure |  |  |  |  | -0.00 | -0.03 – 0.03 | -0.00 | -0.03 – 0.03 |
| Cooking Facility |  |  |  |  | 0.03 | -0.00 – 0.06 | 0.03 | -0.00 – 0.06 |
| Infrastructure |  |  |  |  |  |  | 0.08 | -0.01 – 0.16 |
| Travel time to Cities |  |  |  |  |  |  | 0.05 | -0.08 – 0.19 |
| % Working Women in Urban Areas |  |  |  |  |  |  | -0.03 | -3.05 – 2.98 |
| **Random Effects** | | | | | | | | |
| σ^2^ | 0.34 | | 0.30 | | 0.27 | | 0.27 | |
| (τ_2_)^2^ | 0.13 _District:State_ | | 0.13 _District:State_ | | 0.14 _District:State_ | | 0.14 _District:State_ | |
| (τ_1_)^2^ | 0.19 _State_ | | 0.18 _State_ | | 0.18 _State_ | | 0.18 _State_ | |
| ICC | 0.48 | | 0.51 | | 0.54 | | 0.54 | |
| N | 53 _District_ | | 53 _District_ | | 53 _District_ | | 53 _District_ | |
|  | 32 _State_ | | 32 _State_ | | 32 _State_ | | 32 _State_ | |
| Observations | 6191 | | 6191 | | 6191 | | 6191 | |
| Marginal R^2^ / Conditional R^2^ | 0.000 / 0.482 | | 0.075 / 0.551 | | 0.113 / 0.590 | | 0.116 / 0.593 | |
| ** p<0.05   ** p<0.01   *** p<0.001* | | | | | | | | |

Table S22: Multi-level model estimates for (log) household consumption of *Refined oil* with household- and district-level covariates.

|  | **Refined Oil** | | **Refined Oil** | | **Refined Oil** | | **Refined Oil** | |
| --- | --- | --- | --- | --- | --- | --- | --- | --- |
| *Predictors* | *Estimates* | *CI* | *Estimates* | *CI* | *Estimates* | *CI* | *Estimates* | *CI* |
| Intercept | 7.36 ^***^ | 7.17 – 7.55 | 3.57 ^***^ | 3.35 – 3.78 | 4.03 ^***^ | 3.82 – 4.25 | 3.36 ^***^ | 2.79 – 3.93 |
| Income |  |  | 0.42 ^***^ | 0.41 – 0.43 | 0.29 ^***^ | 0.28 – 0.30 | 0.29 ^***^ | 0.28 – 0.30 |
| Urban |  |  | 0.02 ^**^ | 0.01 – 0.03 | 0.04 ^***^ | 0.03 – 0.05 | 0.04 ^***^ | 0.03 – 0.05 |
| Household Size |  |  |  |  | 0.39 ^***^ | 0.37 – 0.40 | 0.39 ^***^ | 0.37 – 0.40 |
| Household Structure |  |  |  |  | 0.02 ^***^ | 0.01 – 0.03 | 0.02 ^***^ | 0.01 – 0.03 |
| Cooking Facility |  |  |  |  | 0.07 ^***^ | 0.06 – 0.09 | 0.07 ^***^ | 0.06 – 0.09 |
| Infrastructure |  |  |  |  |  |  | 0.02 | -0.02 – 0.06 |
| Travel time to Cities |  |  |  |  |  |  | 0.10 ^**^ | 0.03 – 0.17 |
| % Working Women in Urban Areas |  |  |  |  |  |  | 2.75 ^**^ | 0.88 – 4.62 |
| **Random Effects** | | | | | | | | |
| σ^2^ | 0.32 | | 0.27 | | 0.25 | | 0.25 | |
| (τ_2_)^2^ | 0.07 _District:State_ | | 0.08 _District:State_ | | 0.08 _District:State_ | | 0.08 _District:State_ | |
| (τ_1_)^2^ | 0.32 _State_ | | 0.35 _State_ | | 0.36 _State_ | | 0.35 _State_ | |
| ICC | 0.54 | | 0.62 | | 0.64 | | 0.64 | |
| N | 71 _District_ | | 71 _District_ | | 71 _District_ | | 71 _District_ | |
|  | 35 _State_ | | 35 _State_ | | 35 _State_ | | 35 _State_ | |
| Observations | 44918 | | 44918 | | 44918 | | 44918 | |
| Marginal R^2^ / Conditional R^2^ | 0.000 / 0.544 | | 0.096 / 0.655 | | 0.119 / 0.683 | | 0.127 / 0.684 | |
| ** p<0.05   ** p<0.01   *** p<0.001* | | | | | | | | |

Table S23: Multi-level model estimates for (log) household consumption of *Other oil* with household- and district-level covariates.

|  | **Other Oil** | | **Other Oil** | | **Other Oil** | | **Other Oil** | |
| --- | --- | --- | --- | --- | --- | --- | --- | --- |
| *Predictors* | *Estimates* | *CI* | *Estimates* | *CI* | *Estimates* | *CI* | *Estimates* | *CI* |
| Intercept | 7.08 ^***^ | 6.84 – 7.31 | 5.04 ^***^ | 4.75 – 5.34 | 5.56 ^***^ | 5.27 – 5.86 | 3.53 ^***^ | 2.43 – 4.63 |
| Income |  |  | 0.23 ^***^ | 0.21 – 0.25 | 0.10 ^***^ | 0.07 – 0.12 | 0.10 ^***^ | 0.07 – 0.12 |
| Urban |  |  | 0.02 | -0.01 – 0.04 | 0.06 ^***^ | 0.03 – 0.08 | 0.06 ^***^ | 0.03 – 0.08 |
| Household Size |  |  |  |  | 0.44 ^***^ | 0.40 – 0.47 | 0.44 ^***^ | 0.40 – 0.47 |
| Household Structure |  |  |  |  | -0.01 | -0.04 – 0.01 | -0.01 | -0.04 – 0.01 |
| Cooking Facility |  |  |  |  | -0.05 ^***^ | -0.07 – -0.02 | -0.05 ^***^ | -0.07 – -0.02 |
| Infrastructure |  |  |  |  |  |  | 0.11 ^**^ | 0.03 – 0.20 |
| Travel time to Cities |  |  |  |  |  |  | 0.26 ^***^ | 0.12 – 0.40 |
| % Working Women in Urban Areas |  |  |  |  |  |  | 3.58 ^*^ | 0.02 – 7.13 |
| **Random Effects** | | | | | | | | |
| σ^2^ | 0.37 | | 0.35 | | 0.33 | | 0.33 | |
| (τ_2_)^2^ | 0.18 _District:State_ | | 0.20 _District:State_ | | 0.20 _District:State_ | | 0.19 _District:State_ | |
| (τ_1_)^2^ | 0.42 _State_ | | 0.42 _State_ | | 0.41 _State_ | | 0.36 _State_ | |
| ICC | 0.62 | | 0.64 | | 0.65 | | 0.63 | |
| N | 54 _District_ | | 54 _District_ | | 54 _District_ | | 54 _District_ | |
|  | 34 _State_ | | 34 _State_ | | 34 _State_ | | 34 _State_ | |
| Observations | 11563 | | 11563 | | 11563 | | 11563 | |
| Marginal R^2^ / Conditional R^2^ | 0.000 / 0.620 | | 0.021 / 0.647 | | 0.045 / 0.665 | | 0.065 / 0.649 | |
| ** p<0.05   ** p<0.01   *** p<0.001* | | | | | | | | |

Table S24: Multi-level model estimates for (log) household consumption of *Eggs* with household- and district-level covariates.

|  | **Eggs** | | **Eggs** | | **Eggs** | | **Eggs** | |
| --- | --- | --- | --- | --- | --- | --- | --- | --- |
| *Predictors* | *Estimates* | *CI* | *Estimates* | *CI* | *Estimates* | *CI* | *Estimates* | *CI* |
| Intercept | 2.59 ^***^ | 2.54 – 2.65 | -1.41 ^***^ | -1.50 – -1.31 | -1.10 ^***^ | -1.20 – -1.00 | -1.41 ^***^ | -1.71 – -1.10 |
| Income |  |  | 0.45 ^***^ | 0.44 – 0.46 | 0.37 ^***^ | 0.36 – 0.38 | 0.37 ^***^ | 0.36 – 0.38 |
| Urban |  |  | -0.00 | -0.01 – 0.01 | 0.02 ^*^ | 0.00 – 0.03 | 0.02 ^*^ | 0.00 – 0.03 |
| Household Size |  |  |  |  | 0.26 ^***^ | 0.24 – 0.28 | 0.26 ^***^ | 0.24 – 0.28 |
| Household Structure |  |  |  |  | -0.01 ^*^ | -0.03 – -0.00 | -0.02 ^*^ | -0.03 – -0.00 |
| Cooking Facility |  |  |  |  | 0.03 ^***^ | 0.02 – 0.05 | 0.03 ^***^ | 0.02 – 0.04 |
| Infrastructure |  |  |  |  |  |  | 0.01 | -0.01 – 0.04 |
| Travel time to Cities |  |  |  |  |  |  | 0.04 ^*^ | 0.00 – 0.08 |
| % Working Women in Urban Areas |  |  |  |  |  |  | 1.54 ^**^ | 0.52 – 2.56 |
| **Random Effects** | | | | | | | | |
| σ^2^ | 0.35 | | 0.29 | | 0.28 | | 0.28 | |
| (τ_2_)^2^ | 0.03 _District:State_ | | 0.03 _District:State_ | | 0.03 _District:State_ | | 0.03 _District:State_ | |
| (τ_1_)^2^ | 0.02 _State_ | | 0.01 _State_ | | 0.01 _State_ | | 0.01 _State_ | |
| ICC | 0.14 | | 0.11 | | 0.12 | | 0.11 | |
| N | 71 _District_ | | 71 _District_ | | 71 _District_ | | 71 _District_ | |
|  | 35 _State_ | | 35 _State_ | | 35 _State_ | | 35 _State_ | |
| Observations | 42103 | | 42103 | | 42103 | | 42103 | |
| Marginal R^2^ / Conditional R^2^ | 0.000 / 0.142 | | 0.187 / 0.278 | | 0.202 / 0.296 | | 0.213 / 0.303 | |
| ** p<0.05   ** p<0.01   *** p<0.001* | | | | | | | | |

Table S25: Multi-level model estimates for (log) household consumption of *Mutton* with household- and district-level covariates.

|  | **Mutton** | | **Mutton** | | **Mutton** | | **Mutton** | |
| --- | --- | --- | --- | --- | --- | --- | --- | --- |
| *Predictors* | *Estimates* | *CI* | *Estimates* | *CI* | *Estimates* | *CI* | *Estimates* | *CI* |
| Intercept | 7.01 ^***^ | 6.93 – 7.08 | 2.48 ^***^ | 2.32 – 2.65 | 2.80 ^***^ | 2.63 – 2.97 | 2.79 ^***^ | 2.42 – 3.15 |
| Income |  |  | 0.50 ^***^ | 0.48 – 0.52 | 0.43 ^***^ | 0.41 – 0.45 | 0.43 ^***^ | 0.41 – 0.45 |
| Urban |  |  | -0.09 ^***^ | -0.11 – -0.07 | -0.04 ^***^ | -0.06 – -0.02 | -0.04 ^***^ | -0.06 – -0.02 |
| Household Size |  |  |  |  | 0.28 ^***^ | 0.25 – 0.31 | 0.28 ^***^ | 0.25 – 0.31 |
| Household Structure |  |  |  |  | -0.04 ^***^ | -0.06 – -0.02 | -0.04 ^***^ | -0.07 – -0.02 |
| Cooking Facility |  |  |  |  | -0.03 ^*^ | -0.05 – -0.00 | -0.03 ^*^ | -0.05 – -0.00 |
| Infrastructure |  |  |  |  |  |  | -0.02 | -0.04 – 0.01 |
| Travel time to Cities |  |  |  |  |  |  | 0.02 | -0.02 – 0.06 |
| % Working Women in Urban Areas |  |  |  |  |  |  | -0.09 | -1.22 – 1.04 |
| **Random Effects** | | | | | | | | |
| σ^2^ | 0.38 | | 0.31 | | 0.30 | | 0.30 | |
| (τ_2_)^2^ | 0.02 _District:State_ | | 0.02 _District:State_ | | 0.02 _District:State_ | | 0.02 _District:State_ | |
| (τ_1_)^2^ | 0.04 _State_ | | 0.04 _State_ | | 0.03 _State_ | | 0.02 _State_ | |
| ICC | 0.15 | | 0.15 | | 0.14 | | 0.12 | |
| N | 71 _District_ | | 71 _District_ | | 71 _District_ | | 71 _District_ | |
|  | 35 _State_ | | 35 _State_ | | 35 _State_ | | 35 _State_ | |
| Observations | 16018 | | 16018 | | 16018 | | 16018 | |
| Marginal R^2^ / Conditional R^2^ | 0.000 / 0.150 | | 0.180 / 0.306 | | 0.203 / 0.314 | | 0.207 / 0.302 | |
| ** p<0.05   ** p<0.01   *** p<0.001* | | | | | | | | |

Table S26: Multi-level model estimates for (log) household consumption of *Chicken* with household- and district-level covariates.

|  | **Chicken** | | **Chicken** | | **Chicken** | | **Chicken** | |
| --- | --- | --- | --- | --- | --- | --- | --- | --- |
| *Predictors* | *Estimates* | *CI* | *Estimates* | *CI* | *Estimates* | *CI* | *Estimates* | *CI* |
| Intercept | 7.22 ^***^ | 7.14 – 7.29 | 3.43 ^***^ | 3.33 – 3.53 | 3.75 ^***^ | 3.64 – 3.86 | 3.91 ^***^ | 3.60 – 4.21 |
| Income |  |  | 0.43 ^***^ | 0.42 – 0.44 | 0.35 ^***^ | 0.34 – 0.36 | 0.35 ^***^ | 0.34 – 0.36 |
| Urban |  |  | -0.09 ^***^ | -0.10 – -0.08 | -0.06 ^***^ | -0.07 – -0.04 | -0.06 ^***^ | -0.07 – -0.04 |
| Household Size |  |  |  |  | 0.29 ^***^ | 0.27 – 0.30 | 0.29 ^***^ | 0.27 – 0.30 |
| Household Structure |  |  |  |  | -0.02 ^***^ | -0.03 – -0.01 | -0.02 ^***^ | -0.03 – -0.01 |
| Cooking Facility |  |  |  |  | 0.00 | -0.01 – 0.02 | 0.00 | -0.01 – 0.02 |
| Infrastructure |  |  |  |  |  |  | -0.02 ^*^ | -0.04 – -0.00 |
| Travel time to Cities |  |  |  |  |  |  | -0.01 | -0.05 – 0.03 |
| % Working Women in Urban Areas |  |  |  |  |  |  | 0.72 | -0.27 – 1.72 |
| **Random Effects** | | | | | | | | |
| σ^2^ | 0.32 | | 0.27 | | 0.26 | | 0.26 | |
| (τ_2_)^2^ | 0.02 _District:State_ | | 0.02 _District:State_ | | 0.02 _District:State_ | | 0.02 _District:State_ | |
| (τ_1_)^2^ | 0.04 _State_ | | 0.02 _State_ | | 0.03 _State_ | | 0.02 _State_ | |
| ICC | 0.18 | | 0.14 | | 0.16 | | 0.15 | |
| N | 71 _District_ | | 71 _District_ | | 71 _District_ | | 71 _District_ | |
|  | 35 _State_ | | 35 _State_ | | 35 _State_ | | 35 _State_ | |
| Observations | 39364 | | 39364 | | 39364 | | 39364 | |
| Marginal R^2^ / Conditional R^2^ | 0.000 / 0.175 | | 0.165 / 0.284 | | 0.185 / 0.315 | | 0.190 / 0.312 | |
| ** p<0.05   ** p<0.01   *** p<0.001* | | | | | | | | |

Table S27: Multi-level model estimates for (log) household consumption of *Tomato* with household- and district-level covariates.

|  | **Tomato** | | **Tomato** | | **Tomato** | | **Tomato** | |
| --- | --- | --- | --- | --- | --- | --- | --- | --- |
| *Predictors* | *Estimates* | *CI* | *Estimates* | *CI* | *Estimates* | *CI* | *Estimates* | *CI* |
| Intercept | 7.53 ^***^ | 7.43 – 7.63 | 4.13 ^***^ | 4.01 – 4.25 | 4.52 ^***^ | 4.40 – 4.64 | 4.56 ^***^ | 4.10 – 5.02 |
| Income |  |  | 0.38 ^***^ | 0.38 – 0.39 | 0.28 ^***^ | 0.28 – 0.29 | 0.28 ^***^ | 0.28 – 0.29 |
| Urban |  |  | -0.02 ^***^ | -0.02 – -0.01 | 0.01 ^**^ | 0.00 – 0.02 | 0.01 ^**^ | 0.00 – 0.02 |
| Household Size |  |  |  |  | 0.32 ^***^ | 0.31 – 0.33 | 0.32 ^***^ | 0.31 – 0.33 |
| Household Structure |  |  |  |  | -0.00 | -0.01 – 0.00 | -0.00 | -0.01 – 0.00 |
| Cooking Facility |  |  |  |  | 0.03 ^***^ | 0.02 – 0.04 | 0.03 ^***^ | 0.02 – 0.04 |
| Infrastructure |  |  |  |  |  |  | 0.00 | -0.03 – 0.03 |
| Travel time to Cities |  |  |  |  |  |  | -0.02 | -0.08 – 0.04 |
| % Working Women in Urban Areas |  |  |  |  |  |  | 1.06 | -0.54 – 2.65 |
| **Random Effects** | | | | | | | | |
| σ^2^ | 0.36 | | 0.31 | | 0.30 | | 0.30 | |
| (τ_2_)^2^ | 0.07 _District:State_ | | 0.07 _District:State_ | | 0.06 _District:State_ | | 0.06 _District:State_ | |
| (τ_1_)^2^ | 0.08 _State_ | | 0.09 _State_ | | 0.09 _State_ | | 0.09 _State_ | |
| ICC | 0.28 | | 0.33 | | 0.34 | | 0.34 | |
| N | 71 _District_ | | 71 _District_ | | 71 _District_ | | 71 _District_ | |
|  | 35 _State_ | | 35 _State_ | | 35 _State_ | | 35 _State_ | |
| Observations | 82163 | | 82163 | | 82163 | | 82163 | |
| Marginal R^2^ / Conditional R^2^ | 0.000 / 0.281 | | 0.110 / 0.402 | | 0.131 / 0.426 | | 0.136 / 0.429 | |
| ** p<0.05   ** p<0.01   *** p<0.001* | | | | | | | | |

Table S28: Multi-level model estimates for (log) household consumption of *Carrot* with household- and district-level covariates.

|  | **Carrot** | | **Carrot** | | **Carrot** | | **Carrot** | |
| --- | --- | --- | --- | --- | --- | --- | --- | --- |
| *Predictors* | *Estimates* | *CI* | *Estimates* | *CI* | *Estimates* | *CI* | *Estimates* | *CI* |
| Intercept | 6.93 ^***^ | 6.80 – 7.06 | 4.02 ^***^ | 3.84 – 4.19 | 4.22 ^***^ | 4.04 – 4.39 | 4.08 ^***^ | 3.57 – 4.59 |
| Income |  |  | 0.32 ^***^ | 0.31 – 0.33 | 0.26 ^***^ | 0.24 – 0.27 | 0.26 ^***^ | 0.24 – 0.27 |
| Urban |  |  | -0.02 ^**^ | -0.04 – -0.01 | -0.01 | -0.02 – 0.01 | -0.01 | -0.02 – 0.01 |
| Household Size |  |  |  |  | 0.23 ^***^ | 0.21 – 0.25 | 0.23 ^***^ | 0.21 – 0.25 |
| Household Structure |  |  |  |  | 0.00 | -0.01 – 0.02 | 0.00 | -0.01 – 0.02 |
| Cooking Facility |  |  |  |  | 0.03 ^***^ | 0.01 – 0.05 | 0.03 ^***^ | 0.01 – 0.05 |
| Infrastructure |  |  |  |  |  |  | 0.03 | -0.01 – 0.07 |
| Travel time to Cities |  |  |  |  |  |  | 0.00 | -0.06 – 0.06 |
| % Working Women in Urban Areas |  |  |  |  |  |  | -1.16 | -2.79 – 0.47 |
| **Random Effects** | | | | | | | | |
| σ^2^ | 0.38 | | 0.35 | | 0.34 | | 0.34 | |
| (τ_2_)^2^ | 0.06 _District:State_ | | 0.05 _District:State_ | | 0.05 _District:State_ | | 0.05 _District:State_ | |
| (τ_1_)^2^ | 0.14 _State_ | | 0.14 _State_ | | 0.13 _State_ | | 0.13 _State_ | |
| ICC | 0.34 | | 0.36 | | 0.36 | | 0.35 | |
| N | 71 _District_ | | 71 _District_ | | 71 _District_ | | 71 _District_ | |
|  | 35 _State_ | | 35 _State_ | | 35 _State_ | | 35 _State_ | |
| Observations | 26056 | | 26056 | | 26056 | | 26056 | |
| Marginal R^2^ / Conditional R^2^ | 0.000 / 0.344 | | 0.066 / 0.404 | | 0.079 / 0.407 | | 0.080 / 0.404 | |
| ** p<0.05   ** p<0.01   *** p<0.001* | | | | | | | | |

Table S29: Multi-level model estimates for (log) household consumption of *Okra* with household- and district-level covariates.

|  | **Okra** | | **Okra** | | **Okra** | | **Okra** | |
| --- | --- | --- | --- | --- | --- | --- | --- | --- |
| *Predictors* | *Estimates* | *CI* | *Estimates* | *CI* | *Estimates* | *CI* | *Estimates* | *CI* |
| Intercept | 7.04 ^***^ | 6.95 – 7.14 | 4.39 ^***^ | 4.26 – 4.52 | 4.68 ^***^ | 4.55 – 4.81 | 4.70 ^***^ | 4.26 – 5.14 |
| Income |  |  | 0.30 ^***^ | 0.29 – 0.31 | 0.22 ^***^ | 0.21 – 0.23 | 0.22 ^***^ | 0.21 – 0.23 |
| Urban |  |  | -0.08 ^***^ | -0.09 – -0.07 | -0.04 ^***^ | -0.05 – -0.02 | -0.04 ^***^ | -0.05 – -0.02 |
| Household Size |  |  |  |  | 0.32 ^***^ | 0.30 – 0.33 | 0.32 ^***^ | 0.30 – 0.33 |
| Household Structure |  |  |  |  | -0.01 | -0.02 – 0.00 | -0.01 | -0.02 – 0.00 |
| Cooking Facility |  |  |  |  | -0.01 | -0.02 – 0.01 | -0.01 | -0.02 – 0.01 |
| Infrastructure |  |  |  |  |  |  | 0.00 | -0.03 – 0.03 |
| Travel time to Cities |  |  |  |  |  |  | -0.00 | -0.06 – 0.05 |
| % Working Women in Urban Areas |  |  |  |  |  |  | -0.71 | -2.21 – 0.78 |
| **Random Effects** | | | | | | | | |
| σ^2^ | 0.40 | | 0.37 | | 0.35 | | 0.35 | |
| (τ_2_)^2^ | 0.05 _District:State_ | | 0.05 _District:State_ | | 0.05 _District:State_ | | 0.05 _District:State_ | |
| (τ_1_)^2^ | 0.07 _State_ | | 0.08 _State_ | | 0.07 _State_ | | 0.07 _State_ | |
| ICC | 0.24 | | 0.26 | | 0.25 | | 0.25 | |
| N | 71 _District_ | | 71 _District_ | | 71 _District_ | | 71 _District_ | |
|  | 35 _State_ | | 35 _State_ | | 35 _State_ | | 35 _State_ | |
| Observations | 45765 | | 45765 | | 45765 | | 45765 | |
| Marginal R^2^ / Conditional R^2^ | 0.000 / 0.237 | | 0.065 / 0.307 | | 0.091 / 0.321 | | 0.092 / 0.319 | |
| ** p<0.05   ** p<0.01   *** p<0.001* | | | | | | | | |

Table S30: Multi-level model estimates for (log) household consumption of *Cauliflower* with household- and district-level covariates.

|  | **Cauliflower** | | **Cauliflower** | | **Cauliflower** | | **Cauliflower** | |
| --- | --- | --- | --- | --- | --- | --- | --- | --- |
| *Predictors* | *Estimates* | *CI* | *Estimates* | *CI* | *Estimates* | *CI* | *Estimates* | *CI* |
| Intercept | 7.36 ^***^ | 7.23 – 7.48 | 5.20 ^***^ | 5.04 – 5.36 | 5.57 ^***^ | 5.42 – 5.73 | 5.69 ^***^ | 5.25 – 6.13 |
| Income |  |  | 0.24 ^***^ | 0.23 – 0.25 | 0.15 ^***^ | 0.13 – 0.16 | 0.15 ^***^ | 0.13 – 0.16 |
| Urban |  |  | -0.09 ^***^ | -0.10 – -0.07 | -0.03 ^***^ | -0.05 – -0.02 | -0.03 ^***^ | -0.05 – -0.02 |
| Household Size |  |  |  |  | 0.35 ^***^ | 0.34 – 0.37 | 0.35 ^***^ | 0.34 – 0.37 |
| Household Structure |  |  |  |  | -0.01 ^*^ | -0.02 – -0.00 | -0.01 ^*^ | -0.02 – -0.00 |
| Cooking Facility |  |  |  |  | -0.02 ^**^ | -0.03 – -0.01 | -0.02 ^**^ | -0.03 – -0.01 |
| Infrastructure |  |  |  |  |  |  | -0.00 | -0.03 – 0.03 |
| Travel time to Cities |  |  |  |  |  |  | -0.02 | -0.07 – 0.04 |
| % Working Women in Urban Areas |  |  |  |  |  |  | -0.65 | -2.13 – 0.83 |
| **Random Effects** | | | | | | | | |
| σ^2^ | 0.34 | | 0.32 | | 0.31 | | 0.31 | |
| (τ_2_)^2^ | 0.05 _District:State_ | | 0.05 _District:State_ | | 0.05 _District:State_ | | 0.05 _District:State_ | |
| (τ_1_)^2^ | 0.14 _State_ | | 0.15 _State_ | | 0.14 _State_ | | 0.13 _State_ | |
| ICC | 0.35 | | 0.38 | | 0.37 | | 0.37 | |
| N | 71 _District_ | | 71 _District_ | | 71 _District_ | | 71 _District_ | |
|  | 35 _State_ | | 35 _State_ | | 35 _State_ | | 35 _State_ | |
| Observations | 41295 | | 41295 | | 41295 | | 41295 | |
| Marginal R^2^ / Conditional R^2^ | 0.000 / 0.353 | | 0.041 / 0.409 | | 0.072 / 0.420 | | 0.073 / 0.418 | |
| ** p<0.05   ** p<0.01   *** p<0.001* | | | | | | | | |

Table S31: Multi-level model estimates for (log) household consumption of *Cabbage* with household- and district-level covariates.

|  | **Cabbage** | | **Cabbage** | | **Cabbage** | | **Cabbage** | |
| --- | --- | --- | --- | --- | --- | --- | --- | --- |
| *Predictors* | *Estimates* | *CI* | *Estimates* | *CI* | *Estimates* | *CI* | *Estimates* | *CI* |
| Intercept | 7.32 ^***^ | 7.20 – 7.44 | 5.40 ^***^ | 5.25 – 5.54 | 5.72 ^***^ | 5.57 – 5.86 | 5.84 ^***^ | 5.44 – 6.25 |
| Income |  |  | 0.22 ^***^ | 0.21 – 0.23 | 0.12 ^***^ | 0.11 – 0.13 | 0.12 ^***^ | 0.11 – 0.13 |
| Urban |  |  | -0.07 ^***^ | -0.08 – -0.06 | -0.02 ^**^ | -0.03 – -0.00 | -0.02 ^**^ | -0.03 – -0.00 |
| Household Size |  |  |  |  | 0.36 ^***^ | 0.34 – 0.38 | 0.36 ^***^ | 0.34 – 0.38 |
| Household Structure |  |  |  |  | -0.00 | -0.02 – 0.01 | -0.00 | -0.02 – 0.01 |
| Cooking Facility |  |  |  |  | -0.03 ^***^ | -0.04 – -0.01 | -0.03 ^***^ | -0.04 – -0.01 |
| Infrastructure |  |  |  |  |  |  | -0.02 | -0.05 – 0.01 |
| Travel time to Cities |  |  |  |  |  |  | 0.00 | -0.05 – 0.05 |
| % Working Women in Urban Areas |  |  |  |  |  |  | 0.14 | -1.23 – 1.52 |
| **Random Effects** | | | | | | | | |
| σ^2^ | 0.32 | | 0.31 | | 0.29 | | 0.29 | |
| (τ_2_)^2^ | 0.04 _District:State_ | | 0.04 _District:State_ | | 0.04 _District:State_ | | 0.04 _District:State_ | |
| (τ_1_)^2^ | 0.12 _State_ | | 0.13 _State_ | | 0.11 _State_ | | 0.10 _State_ | |
| ICC | 0.33 | | 0.35 | | 0.35 | | 0.33 | |
| N | 71 _District_ | | 71 _District_ | | 71 _District_ | | 71 _District_ | |
|  | 35 _State_ | | 35 _State_ | | 35 _State_ | | 35 _State_ | |
| Observations | 46893 | | 46893 | | 46893 | | 46893 | |
| Marginal R^2^ / Conditional R^2^ | 0.000 / 0.332 | | 0.036 / 0.378 | | 0.073 / 0.397 | | 0.077 / 0.383 | |
| ** p<0.05   ** p<0.01   *** p<0.001* | | | | | | | | |

Table S32: Multi-level model estimates for (log) household consumption of *Peas* with household- and district-level covariates.

|  | **Peas** | | **Peas** | | **Peas** | | **Peas** | |
| --- | --- | --- | --- | --- | --- | --- | --- | --- |
| *Predictors* | *Estimates* | *CI* | *Estimates* | *CI* | *Estimates* | *CI* | *Estimates* | *CI* |
| Intercept | 6.90 ^***^ | 6.74 – 7.06 | 4.21 ^***^ | 3.99 – 4.43 | 4.46 ^***^ | 4.24 – 4.68 | 4.31 ^***^ | 3.65 – 4.97 |
| Income |  |  | 0.30 ^***^ | 0.28 – 0.32 | 0.23 ^***^ | 0.21 – 0.25 | 0.23 ^***^ | 0.21 – 0.25 |
| Urban |  |  | -0.03 ^***^ | -0.06 – -0.01 | 0.01 | -0.01 – 0.03 | 0.01 | -0.01 – 0.03 |
| Household Size |  |  |  |  | 0.28 ^***^ | 0.25 – 0.30 | 0.28 ^***^ | 0.25 – 0.30 |
| Household Structure |  |  |  |  | -0.01 | -0.03 – 0.01 | -0.01 | -0.03 – 0.01 |
| Cooking Facility |  |  |  |  | -0.02 | -0.04 – 0.01 | -0.02 | -0.04 – 0.01 |
| Infrastructure |  |  |  |  |  |  | -0.00 | -0.05 – 0.05 |
| Travel time to Cities |  |  |  |  |  |  | 0.03 | -0.05 – 0.12 |
| % Working Women in Urban Areas |  |  |  |  |  |  | -0.61 | -2.87 – 1.65 |
| **Random Effects** | | | | | | | | |
| σ^2^ | 0.43 | | 0.40 | | 0.39 | | 0.39 | |
| (τ_2_)^2^ | 0.10 _District:State_ | | 0.10 _District:State_ | | 0.10 _District:State_ | | 0.10 _District:State_ | |
| (τ_1_)^2^ | 0.20 _State_ | | 0.22 _State_ | | 0.20 _State_ | | 0.19 _State_ | |
| ICC | 0.42 | | 0.45 | | 0.44 | | 0.43 | |
| N | 71 _District_ | | 71 _District_ | | 71 _District_ | | 71 _District_ | |
|  | 35 _State_ | | 35 _State_ | | 35 _State_ | | 35 _State_ | |
| Observations | 19045 | | 19045 | | 19045 | | 19045 | |
| Marginal R^2^ / Conditional R^2^ | 0.000 / 0.417 | | 0.047 / 0.475 | | 0.059 / 0.473 | | 0.060 / 0.469 | |
| ** p<0.05   ** p<0.01   *** p<0.001* | | | | | | | | |

Table S33: Multi-level model estimates for (log) household consumption of *Beans* with household- and district-level covariates.

|  | **Beans** | | **Beans** | | **Beans** | | **Beans** | |
| --- | --- | --- | --- | --- | --- | --- | --- | --- |
| *Predictors* | *Estimates* | *CI* | *Estimates* | *CI* | *Estimates* | *CI* | *Estimates* | *CI* |
| Intercept | 6.96 ^***^ | 6.88 – 7.04 | 4.56 ^***^ | 4.43 – 4.69 | 4.79 ^***^ | 4.65 – 4.92 | 4.89 ^***^ | 4.38 – 5.40 |
| Income |  |  | 0.27 ^***^ | 0.26 – 0.28 | 0.20 ^***^ | 0.19 – 0.21 | 0.20 ^***^ | 0.19 – 0.21 |
| Urban |  |  | -0.09 ^***^ | -0.11 – -0.08 | -0.05 ^***^ | -0.07 – -0.04 | -0.05 ^***^ | -0.07 – -0.04 |
| Household Size |  |  |  |  | 0.30 ^***^ | 0.28 – 0.32 | 0.30 ^***^ | 0.28 – 0.32 |
| Household Structure |  |  |  |  | -0.02 ^*^ | -0.03 – -0.00 | -0.02 ^*^ | -0.03 – -0.00 |
| Cooking Facility |  |  |  |  | -0.02 ^*^ | -0.03 – -0.00 | -0.02 ^*^ | -0.03 – -0.00 |
| Infrastructure |  |  |  |  |  |  | -0.04 ^*^ | -0.08 – -0.01 |
| Travel time to Cities |  |  |  |  |  |  | 0.03 | -0.04 – 0.09 |
| % Working Women in Urban Areas |  |  |  |  |  |  | 0.51 | -1.24 – 2.26 |
| **Random Effects** | | | | | | | | |
| σ^2^ | 0.38 | | 0.35 | | 0.34 | | 0.34 | |
| (τ_2_)^2^ | 0.08 _District:State_ | | 0.08 _District:State_ | | 0.08 _District:State_ | | 0.08 _District:State_ | |
| (τ_1_)^2^ | 0.05 _State_ | | 0.05 _State_ | | 0.04 _State_ | | 0.03 _State_ | |
| ICC | 0.26 | | 0.27 | | 0.27 | | 0.25 | |
| N | 71 _District_ | | 71 _District_ | | 71 _District_ | | 71 _District_ | |
|  | 35 _State_ | | 35 _State_ | | 35 _State_ | | 35 _State_ | |
| Observations | 33025 | | 33025 | | 33025 | | 33025 | |
| Marginal R^2^ / Conditional R^2^ | 0.000 / 0.255 | | 0.055 / 0.310 | | 0.077 / 0.323 | | 0.087 / 0.311 | |
| ** p<0.05   ** p<0.01   *** p<0.001* | | | | | | | | |

Table S34: Multi-level model estimates for (log) household consumption of *Lemon* with household- and district-level covariates.

|  | **Lemon** | | **Lemon** | | **Lemon** | | **Lemon** | |
| --- | --- | --- | --- | --- | --- | --- | --- | --- |
| *Predictors* | *Estimates* | *CI* | *Estimates* | *CI* | *Estimates* | *CI* | *Estimates* | *CI* |
| Intercept | 2.02 ^***^ | 1.91 – 2.12 | -1.37 ^***^ | -1.50 – -1.24 | -1.19 ^***^ | -1.33 – -1.06 | -1.24 ^***^ | -1.69 – -0.78 |
| Income |  |  | 0.38 ^***^ | 0.37 – 0.39 | 0.33 ^***^ | 0.32 – 0.34 | 0.33 ^***^ | 0.32 – 0.34 |
| Urban |  |  | -0.02 ^***^ | -0.03 – -0.01 | -0.01 | -0.02 – 0.01 | -0.01 | -0.02 – 0.01 |
| Household Size |  |  |  |  | 0.15 ^***^ | 0.14 – 0.17 | 0.15 ^***^ | 0.14 – 0.17 |
| Household Structure |  |  |  |  | -0.01 | -0.02 – 0.00 | -0.01 | -0.02 – 0.00 |
| Cooking Facility |  |  |  |  | 0.01 | -0.00 – 0.02 | 0.01 | -0.00 – 0.02 |
| Infrastructure |  |  |  |  |  |  | 0.01 | -0.02 – 0.05 |
| Travel time to Cities |  |  |  |  |  |  | -0.00 | -0.06 – 0.06 |
| % Working Women in Urban Areas |  |  |  |  |  |  | -1.06 | -2.64 – 0.52 |
| **Random Effects** | | | | | | | | |
| σ^2^ | 0.36 | | 0.32 | | 0.31 | | 0.31 | |
| (τ_2_)^2^ | 0.06 _District:State_ | | 0.06 _District:State_ | | 0.06 _District:State_ | | 0.06 _District:State_ | |
| (τ_1_)^2^ | 0.09 _State_ | | 0.07 _State_ | | 0.07 _State_ | | 0.07 _State_ | |
| ICC | 0.29 | | 0.29 | | 0.29 | | 0.28 | |
| N | 71 _District_ | | 71 _District_ | | 71 _District_ | | 71 _District_ | |
|  | 35 _State_ | | 35 _State_ | | 35 _State_ | | 35 _State_ | |
| Observations | 41581 | | 41581 | | 41581 | | 41581 | |
| Marginal R^2^ / Conditional R^2^ | 0.000 / 0.287 | | 0.110 / 0.367 | | 0.114 / 0.370 | | 0.115 / 0.367 | |
| ** p<0.05   ** p<0.01   *** p<0.001* | | | | | | | | |

Table S35: Multi-level model estimates for (log) household consumption of *Banana* with household- and district-level covariates.

|  | **Banana** | | **Banana** | | **Banana** | | **Banana** | |
| --- | --- | --- | --- | --- | --- | --- | --- | --- |
| *Predictors* | *Estimates* | *CI* | *Estimates* | *CI* | *Estimates* | *CI* | *Estimates* | *CI* |
| Intercept | 2.94 ^***^ | 2.87 – 3.02 | -0.78 ^***^ | -0.88 – -0.68 | -0.59 ^***^ | -0.70 – -0.49 | -0.57 ^***^ | -0.90 – -0.24 |
| Income |  |  | 0.42 ^***^ | 0.41 – 0.42 | 0.37 ^***^ | 0.37 – 0.38 | 0.37 ^***^ | 0.37 – 0.38 |
| Urban |  |  | -0.05 ^***^ | -0.06 – -0.04 | -0.04 ^***^ | -0.05 – -0.03 | -0.04 ^***^ | -0.05 – -0.03 |
| Household Size |  |  |  |  | 0.14 ^***^ | 0.12 – 0.15 | 0.14 ^***^ | 0.12 – 0.15 |
| Household Structure |  |  |  |  | -0.02 ^**^ | -0.03 – -0.01 | -0.02 ^**^ | -0.03 – -0.01 |
| Cooking Facility |  |  |  |  | 0.03 ^***^ | 0.01 – 0.04 | 0.03 ^***^ | 0.01 – 0.04 |
| Infrastructure |  |  |  |  |  |  | 0.00 | -0.02 – 0.03 |
| Travel time to Cities |  |  |  |  |  |  | -0.01 | -0.05 – 0.03 |
| % Working Women in Urban Areas |  |  |  |  |  |  | -0.18 | -1.30 – 0.94 |
| **Random Effects** | | | | | | | | |
| σ^2^ | 0.35 | | 0.30 | | 0.30 | | 0.30 | |
| (τ_2_)^2^ | 0.03 _District:State_ | | 0.03 _District:State_ | | 0.03 _District:State_ | | 0.03 _District:State_ | |
| (τ_1_)^2^ | 0.04 _State_ | | 0.04 _State_ | | 0.04 _State_ | | 0.04 _State_ | |
| ICC | 0.18 | | 0.18 | | 0.19 | | 0.19 | |
| N | 71 _District_ | | 71 _District_ | | 71 _District_ | | 71 _District_ | |
|  | 35 _State_ | | 35 _State_ | | 35 _State_ | | 35 _State_ | |
| Observations | 56144 | | 56144 | | 56144 | | 56144 | |
| Marginal R^2^ / Conditional R^2^ | 0.000 / 0.177 | | 0.149 / 0.306 | | 0.153 / 0.310 | | 0.153 / 0.313 | |
| ** p<0.05   ** p<0.01   *** p<0.001* | | | | | | | | |

Table S36: Multi-level model estimates for (log) household consumption of *Coconut* with household- and district-level covariates.

|  | **Coconut** | | **Coconut** | | **Coconut** | | **Coconut** | |
| --- | --- | --- | --- | --- | --- | --- | --- | --- |
| *Predictors* | *Estimates* | *CI* | *Estimates* | *CI* | *Estimates* | *CI* | *Estimates* | *CI* |
| Intercept | 1.29 ^***^ | 1.04 – 1.53 | -1.20 ^***^ | -1.47 – -0.93 | -1.11 ^***^ | -1.38 – -0.83 | -1.31 ^***^ | -2.04 – -0.58 |
| Income |  |  | 0.27 ^***^ | 0.26 – 0.28 | 0.23 ^***^ | 0.22 – 0.24 | 0.23 ^***^ | 0.22 – 0.24 |
| Urban |  |  | -0.08 ^***^ | -0.09 – -0.07 | -0.06 ^***^ | -0.08 – -0.05 | -0.07 ^***^ | -0.08 – -0.05 |
| Household Size |  |  |  |  | 0.16 ^***^ | 0.14 – 0.18 | 0.16 ^***^ | 0.14 – 0.18 |
| Household Structure |  |  |  |  | 0.02 ^**^ | 0.01 – 0.03 | 0.02 ^**^ | 0.01 – 0.03 |
| Cooking Facility |  |  |  |  | 0.01 | -0.00 – 0.03 | 0.01 | -0.00 – 0.03 |
| Infrastructure |  |  |  |  |  |  | -0.01 | -0.06 – 0.05 |
| Travel time to Cities |  |  |  |  |  |  | 0.03 | -0.06 – 0.13 |
| % Working Women in Urban Areas |  |  |  |  |  |  | 2.88 ^*^ | 0.63 – 5.13 |
| **Random Effects** | | | | | | | | |
| σ^2^ | 0.24 | | 0.22 | | 0.21 | | 0.21 | |
| (τ_2_)^2^ | 0.08 _District:State_ | | 0.08 _District:State_ | | 0.09 _District:State_ | | 0.08 _District:State_ | |
| (τ_1_)^2^ | 0.49 _State_ | | 0.51 _State_ | | 0.52 _State_ | | 0.49 _State_ | |
| ICC | 0.70 | | 0.73 | | 0.74 | | 0.73 | |
| N | 59 _District_ | | 59 _District_ | | 59 _District_ | | 59 _District_ | |
|  | 34 _State_ | | 34 _State_ | | 34 _State_ | | 34 _State_ | |
| Observations | 22881 | | 22881 | | 22881 | | 22881 | |
| Marginal R^2^ / Conditional R^2^ | 0.000 / 0.704 | | 0.035 / 0.743 | | 0.038 / 0.752 | | 0.045 / 0.744 | |
| ** p<0.05   ** p<0.01   *** p<0.001* | | | | | | | | |

Table S37: Multi-level model estimates for (log) household consumption of *Green* *coconut* with household- and district-level covariates.

|  | **Green Coconut** | | **Green Coconut** | | **Green Coconut** | | **Green Coconut** | |
| --- | --- | --- | --- | --- | --- | --- | --- | --- |
| *Predictors* | *Estimates* | *CI* | *Estimates* | *CI* | *Estimates* | *CI* | *Estimates* | *CI* |
| Intercept | 1.39 ^***^ | 1.22 – 1.55 | -0.88 ^***^ | -1.20 – -0.56 | -0.86 ^***^ | -1.20 – -0.53 | -1.64 ^***^ | -2.58 – -0.69 |
| Income |  |  | 0.25 ^***^ | 0.22 – 0.28 | 0.24 ^***^ | 0.21 – 0.28 | 0.25 ^***^ | 0.21 – 0.28 |
| Urban |  |  | -0.05 ^*^ | -0.09 – -0.01 | -0.04 | -0.09 – 0.00 | -0.04 | -0.08 – 0.00 |
| Household Size |  |  |  |  | 0.03 | -0.02 – 0.09 | 0.03 | -0.03 – 0.09 |
| Household Structure |  |  |  |  | -0.01 | -0.05 – 0.03 | -0.01 | -0.05 – 0.03 |
| Cooking Facility |  |  |  |  | -0.01 | -0.05 – 0.04 | -0.01 | -0.06 – 0.04 |
| Infrastructure |  |  |  |  |  |  | 0.02 | -0.06 – 0.09 |
| Travel time to Cities |  |  |  |  |  |  | 0.13 ^*^ | 0.02 – 0.23 |
| % Working Women in Urban Areas |  |  |  |  |  |  | 1.10 | -1.43 – 3.63 |
| **Random Effects** | | | | | | | | |
| σ^2^ | 0.38 | | 0.36 | | 0.36 | | 0.36 | |
| (τ_2_)^2^ | 0.06 _District:State_ | | 0.07 _District:State_ | | 0.07 _District:State_ | | 0.07 _District:State_ | |
| (τ_1_)^2^ | 0.17 _State_ | | 0.16 _State_ | | 0.16 _State_ | | 0.15 _State_ | |
| ICC | 0.38 | | 0.39 | | 0.39 | | 0.38 | |
| N | 46 _District_ | | 46 _District_ | | 46 _District_ | | 46 _District_ | |
|  | 31 _State_ | | 31 _State_ | | 31 _State_ | | 31 _State_ | |
| Observations | 4446 | | 4446 | | 4446 | | 4446 | |
| Marginal R^2^ / Conditional R^2^ | 0.000 / 0.384 | | 0.041 / 0.412 | | 0.041 / 0.414 | | 0.046 / 0.407 | |
| ** p<0.05   ** p<0.01   *** p<0.001* | | | | | | | | |

Table S38: Multi-level model estimates for (log) household consumption of *Orange* with household- and district-level covariates.

|  | **Orange** | | **Orange** | | **Orange** | | **Orange** | |
| --- | --- | --- | --- | --- | --- | --- | --- | --- |
| *Predictors* | *Estimates* | *CI* | *Estimates* | *CI* | *Estimates* | *CI* | *Estimates* | *CI* |
| Intercept | 2.37 ^***^ | 2.27 – 2.47 | -0.87 ^***^ | -1.04 – -0.70 | -0.75 ^***^ | -0.93 – -0.57 | -0.81 ^***^ | -1.29 – -0.33 |
| Income |  |  | 0.36 ^***^ | 0.34 – 0.37 | 0.33 ^***^ | 0.31 – 0.35 | 0.33 ^***^ | 0.31 – 0.35 |
| Urban |  |  | -0.03 ^**^ | -0.05 – -0.01 | -0.02 ^*^ | -0.04 – -0.00 | -0.02 ^*^ | -0.05 – -0.00 |
| Household Size |  |  |  |  | 0.11 ^***^ | 0.08 – 0.13 | 0.11 ^***^ | 0.08 – 0.13 |
| Household Structure |  |  |  |  | -0.01 | -0.03 – 0.01 | -0.01 | -0.03 – 0.01 |
| Cooking Facility |  |  |  |  | 0.02 | -0.00 – 0.04 | 0.02 | -0.00 – 0.04 |
| Infrastructure |  |  |  |  |  |  | -0.02 | -0.06 – 0.02 |
| Travel time to Cities |  |  |  |  |  |  | 0.03 | -0.03 – 0.09 |
| % Working Women in Urban Areas |  |  |  |  |  |  | 1.45 | -0.14 – 3.05 |
| **Random Effects** | | | | | | | | |
| σ^2^ | 0.34 | | 0.30 | | 0.30 | | 0.30 | |
| (τ_2_)^2^ | 0.05 _District:State_ | | 0.05 _District:State_ | | 0.05 _District:State_ | | 0.05 _District:State_ | |
| (τ_1_)^2^ | 0.08 _State_ | | 0.07 _State_ | | 0.07 _State_ | | 0.07 _State_ | |
| ICC | 0.28 | | 0.29 | | 0.29 | | 0.28 | |
| N | 70 _District_ | | 70 _District_ | | 70 _District_ | | 70 _District_ | |
|  | 35 _State_ | | 35 _State_ | | 35 _State_ | | 35 _State_ | |
| Observations | 15274 | | 15274 | | 15274 | | 15274 | |
| Marginal R^2^ / Conditional R^2^ | 0.000 / 0.278 | | 0.103 / 0.360 | | 0.105 / 0.360 | | 0.109 / 0.356 | |
| ** p<0.05   ** p<0.01   *** p<0.001* | | | | | | | | |

Table S39: Multi-level model estimates for (log) household consumption of *Papaya* with household- and district-level covariates.

|  | **Papaya** | | **Papaya** | | **Papaya** | | **Papaya** | |
| --- | --- | --- | --- | --- | --- | --- | --- | --- |
| *Predictors* | *Estimates* | *CI* | *Estimates* | *CI* | *Estimates* | *CI* | *Estimates* | *CI* |
| Intercept | 7.46 ^***^ | 7.38 – 7.54 | 4.71 ^***^ | 4.51 – 4.91 | 4.80 ^***^ | 4.59 – 5.01 | 4.83 ^***^ | 4.35 – 5.30 |
| Income |  |  | 0.30 ^***^ | 0.28 – 0.33 | 0.28 ^***^ | 0.26 – 0.30 | 0.28 ^***^ | 0.26 – 0.31 |
| Urban |  |  | -0.07 ^***^ | -0.09 – -0.04 | -0.02 | -0.05 – 0.01 | -0.02 | -0.05 – 0.01 |
| Household Size |  |  |  |  | 0.16 ^***^ | 0.12 – 0.19 | 0.15 ^***^ | 0.12 – 0.19 |
| Household Structure |  |  |  |  | -0.03 ^*^ | -0.06 – -0.01 | -0.03 ^*^ | -0.06 – -0.01 |
| Cooking Facility |  |  |  |  | -0.06 ^***^ | -0.09 – -0.02 | -0.05 ^***^ | -0.09 – -0.02 |
| Infrastructure |  |  |  |  |  |  | -0.02 | -0.05 – 0.01 |
| Travel time to Cities |  |  |  |  |  |  | 0.02 | -0.04 – 0.07 |
| % Working Women in Urban Areas |  |  |  |  |  |  | -0.73 | -2.33 – 0.87 |
| **Random Effects** | | | | | | | | |
| σ^2^ | 0.33 | | 0.30 | | 0.29 | | 0.29 | |
| (τ_2_)^2^ | 0.04 _District:State_ | | 0.04 _District:State_ | | 0.04 _District:State_ | | 0.04 _District:State_ | |
| (τ_1_)^2^ | 0.05 _State_ | | 0.04 _State_ | | 0.04 _State_ | | 0.03 _State_ | |
| ICC | 0.20 | | 0.21 | | 0.21 | | 0.19 | |
| N | 70 _District_ | | 70 _District_ | | 70 _District_ | | 70 _District_ | |
|  | 35 _State_ | | 35 _State_ | | 35 _State_ | | 35 _State_ | |
| Observations | 8694 | | 8694 | | 8694 | | 8694 | |
| Marginal R^2^ / Conditional R^2^ | 0.000 / 0.201 | | 0.093 / 0.287 | | 0.099 / 0.287 | | 0.098 / 0.273 | |
| ** p<0.05   ** p<0.01   *** p<0.001* | | | | | | | | |

Table S40: Multi-level model estimates for (log) household consumption of *Pears* with household- and district-level covariates.

|  | **Pears** | | **Pears** | | **Pears** | | **Pears** | |
| --- | --- | --- | --- | --- | --- | --- | --- | --- |
| *Predictors* | *Estimates* | *CI* | *Estimates* | *CI* | *Estimates* | *CI* | *Estimates* | *CI* |
| Intercept | 6.99 ^***^ | 6.80 – 7.18 | 3.83 ^***^ | 3.16 – 4.51 | 4.02 ^***^ | 3.29 – 4.75 | 3.88 ^***^ | 2.54 – 5.21 |
| Income |  |  | 0.35 ^***^ | 0.28 – 0.42 | 0.33 ^***^ | 0.24 – 0.41 | 0.33 ^***^ | 0.24 – 0.41 |
| Urban |  |  | -0.18 ^***^ | -0.28 – -0.09 | -0.14 ^*^ | -0.25 – -0.03 | -0.15 ^**^ | -0.26 – -0.04 |
| Household Size |  |  |  |  | 0.15 ^*^ | 0.03 – 0.28 | 0.16 ^*^ | 0.03 – 0.28 |
| Household Structure |  |  |  |  | -0.07 | -0.17 – 0.03 | -0.07 | -0.17 – 0.03 |
| Cooking Facility |  |  |  |  | -0.05 | -0.16 – 0.06 | -0.05 | -0.16 – 0.06 |
| Infrastructure |  |  |  |  |  |  | -0.00 | -0.09 – 0.08 |
| Travel time to Cities |  |  |  |  |  |  | 0.03 | -0.12 – 0.17 |
| % Working Women in Urban Areas |  |  |  |  |  |  | 1.94 | -2.46 – 6.33 |
| **Random Effects** | | | | | | | | |
| σ^2^ | 0.34 | | 0.30 | | 0.30 | | 0.30 | |
| (τ_2_)^2^ | 0.05 _District:State_ | | 0.05 _District:State_ | | 0.05 _District:State_ | | 0.05 _District:State_ | |
| (τ_1_)^2^ | 0.20 _State_ | | 0.17 _State_ | | 0.17 _State_ | | 0.16 _State_ | |
| ICC | 0.43 | | 0.42 | | 0.42 | | 0.42 | |
| N | 51 _District_ | | 51 _District_ | | 51 _District_ | | 51 _District_ | |
|  | 28 _State_ | | 28 _State_ | | 28 _State_ | | 28 _State_ | |
| Observations | 820 | | 820 | | 820 | | 820 | |
| Marginal R^2^ / Conditional R^2^ | 0.000 / 0.433 | | 0.080 / 0.470 | | 0.086 / 0.469 | | 0.090 / 0.471 | |
| ** p<0.05   ** p<0.01   *** p<0.001* | | | | | | | | |

Table S41: Multi-level model estimates for (log) household consumption of *Lichi* with household- and district-level covariates.

|  | **Lichi** | | **Lichi** | | **Lichi** | | **Lichi** | |
| --- | --- | --- | --- | --- | --- | --- | --- | --- |
| *Predictors* | *Estimates* | *CI* | *Estimates* | *CI* | *Estimates* | *CI* | *Estimates* | *CI* |
| Intercept | 6.84 ^***^ | 6.73 – 6.96 | 2.49 ^***^ | 1.92 – 3.06 | 2.96 ^***^ | 2.35 – 3.58 | 2.60 ^***^ | 1.41 – 3.80 |
| Income |  |  | 0.48 ^***^ | 0.41 – 0.54 | 0.40 ^***^ | 0.33 – 0.48 | 0.41 ^***^ | 0.34 – 0.48 |
| Urban |  |  | -0.05 | -0.13 – 0.03 | -0.06 | -0.15 – 0.03 | -0.05 | -0.14 – 0.04 |
| Household Size |  |  |  |  | 0.20 ^***^ | 0.11 – 0.30 | 0.20 ^***^ | 0.11 – 0.30 |
| Household Structure |  |  |  |  | -0.07 | -0.15 – 0.00 | -0.07 | -0.15 – 0.00 |
| Cooking Facility |  |  |  |  | 0.10 ^*^ | 0.01 – 0.20 | 0.11 ^*^ | 0.02 – 0.20 |
| Infrastructure |  |  |  |  |  |  | 0.05 | -0.03 – 0.14 |
| Travel time to Cities |  |  |  |  |  |  | 0.03 | -0.11 – 0.16 |
| % Working Women in Urban Areas |  |  |  |  |  |  | -4.54 ^*^ | -8.58 – -0.50 |
| **Random Effects** | | | | | | | | |
| σ^2^ | 0.36 | | 0.30 | | 0.29 | | 0.29 | |
| (τ_2_)^2^ | 0.13 _District:State_ | | 0.12 _District:State_ | | 0.12 _District:State_ | | 0.13 _District:State_ | |
| (τ_1_)^2^ | 0.06 _State_ | | 0.07 _State_ | | 0.06 _State_ | | 0.06 _State_ | |
| ICC | 0.34 | | 0.39 | | 0.39 | | 0.38 | |
| N | 56 _District_ | | 56 _District_ | | 56 _District_ | | 56 _District_ | |
|  | 30 _State_ | | 30 _State_ | | 30 _State_ | | 30 _State_ | |
| Observations | 1276 | | 1276 | | 1276 | | 1276 | |
| Marginal R^2^ / Conditional R^2^ | 0.000 / 0.342 | | 0.151 / 0.480 | | 0.155 / 0.486 | | 0.159 / 0.481 | |
| ** p<0.05   ** p<0.01   *** p<0.001* | | | | | | | | |

Table S42: Multi-level model estimates for (log) household consumption of *Apple* with household- and district-level covariates.

|  | **Apple** | | **Apple** | | **Apple** | | **Apple** | |
| --- | --- | --- | --- | --- | --- | --- | --- | --- |
| *Predictors* | *Estimates* | *CI* | *Estimates* | *CI* | *Estimates* | *CI* | *Estimates* | *CI* |
| Intercept | 6.99 ^***^ | 6.89 – 7.09 | 2.83 ^***^ | 2.68 – 2.98 | 2.98 ^***^ | 2.82 – 3.14 | 2.88 ^***^ | 2.49 – 3.28 |
| Income |  |  | 0.45 ^***^ | 0.44 – 0.46 | 0.43 ^***^ | 0.41 – 0.44 | 0.43 ^***^ | 0.41 – 0.44 |
| Urban |  |  | 0.02 ^*^ | 0.00 – 0.04 | -0.01 | -0.03 – 0.01 | -0.01 | -0.02 – 0.01 |
| Household Size |  |  |  |  | 0.04 ^***^ | 0.02 – 0.06 | 0.04 ^***^ | 0.02 – 0.06 |
| Household Structure |  |  |  |  | -0.02 ^**^ | -0.04 – -0.01 | -0.02 ^**^ | -0.04 – -0.01 |
| Cooking Facility |  |  |  |  | 0.09 ^***^ | 0.07 – 0.11 | 0.09 ^***^ | 0.07 – 0.11 |
| Infrastructure |  |  |  |  |  |  | 0.01 | -0.02 – 0.04 |
| Travel time to Cities |  |  |  |  |  |  | 0.01 | -0.04 – 0.06 |
| % Working Women in Urban Areas |  |  |  |  |  |  | -0.57 | -1.84 – 0.70 |
| **Random Effects** | | | | | | | | |
| σ^2^ | 0.37 | | 0.31 | | 0.31 | | 0.31 | |
| (τ_2_)^2^ | 0.04 _District:State_ | | 0.03 _District:State_ | | 0.03 _District:State_ | | 0.03 _District:State_ | |
| (τ_1_)^2^ | 0.08 _State_ | | 0.07 _State_ | | 0.07 _State_ | | 0.07 _State_ | |
| ICC | 0.24 | | 0.25 | | 0.25 | | 0.25 | |
| N | 71 _District_ | | 71 _District_ | | 71 _District_ | | 71 _District_ | |
|  | 35 _State_ | | 35 _State_ | | 35 _State_ | | 35 _State_ | |
| Observations | 23468 | | 23468 | | 23468 | | 23468 | |
| Marginal R^2^ / Conditional R^2^ | 0.000 / 0.244 | | 0.152 / 0.360 | | 0.155 / 0.365 | | 0.154 / 0.363 | |
| ** p<0.05   ** p<0.01   *** p<0.001* | | | | | | | | |

Table S43: Multi-level model estimates for (log) household consumption of *Grapes* with household- and district-level covariates.

|  | **Grapes** | | **Grapes** | | **Grapes** | | **Grapes** | |
| --- | --- | --- | --- | --- | --- | --- | --- | --- |
| *Predictors* | *Estimates* | *CI* | *Estimates* | *CI* | *Estimates* | *CI* | *Estimates* | *CI* |
| Intercept | 6.62 ^***^ | 6.50 – 6.73 | 3.14 ^***^ | 2.96 – 3.32 | 3.38 ^***^ | 3.20 – 3.57 | 3.40 ^***^ | 2.89 – 3.91 |
| Income |  |  | 0.38 ^***^ | 0.36 – 0.40 | 0.33 ^***^ | 0.31 – 0.35 | 0.33 ^***^ | 0.31 – 0.35 |
| Urban |  |  | 0.01 | -0.01 – 0.03 | -0.00 | -0.02 – 0.02 | -0.00 | -0.02 – 0.02 |
| Household Size |  |  |  |  | 0.14 ^***^ | 0.11 – 0.17 | 0.14 ^***^ | 0.11 – 0.17 |
| Household Structure |  |  |  |  | -0.02 ^*^ | -0.04 – -0.00 | -0.02 ^*^ | -0.04 – -0.00 |
| Cooking Facility |  |  |  |  | 0.07 ^***^ | 0.05 – 0.10 | 0.07 ^***^ | 0.05 – 0.10 |
| Infrastructure |  |  |  |  |  |  | 0.02 | -0.02 – 0.06 |
| Travel time to Cities |  |  |  |  |  |  | -0.02 | -0.08 – 0.04 |
| % Working Women in Urban Areas |  |  |  |  |  |  | -0.69 | -2.38 – 0.99 |
| **Random Effects** | | | | | | | | |
| σ^2^ | 0.36 | | 0.31 | | 0.31 | | 0.31 | |
| (τ_2_)^2^ | 0.06 _District:State_ | | 0.05 _District:State_ | | 0.05 _District:State_ | | 0.05 _District:State_ | |
| (τ_1_)^2^ | 0.10 _State_ | | 0.08 _State_ | | 0.08 _State_ | | 0.08 _State_ | |
| ICC | 0.31 | | 0.29 | | 0.30 | | 0.29 | |
| N | 71 _District_ | | 71 _District_ | | 71 _District_ | | 71 _District_ | |
|  | 35 _State_ | | 35 _State_ | | 35 _State_ | | 35 _State_ | |
| Observations | 14736 | | 14736 | | 14736 | | 14736 | |
| Marginal R^2^ / Conditional R^2^ | 0.000 / 0.310 | | 0.111 / 0.373 | | 0.115 / 0.377 | | 0.117 / 0.377 | |
| ** p<0.05   ** p<0.01   *** p<0.001* | | | | | | | | |

Table S44: Multi-level model estimates for (log) household consumption of *Dry coconut* with household- and district-level covariates.

|  | **Dry Coconut** | | **Dry Coconut** | | **Dry Coconut** | | **Dry Coconut** | |
| --- | --- | --- | --- | --- | --- | --- | --- | --- |
| *Predictors* | *Estimates* | *CI* | *Estimates* | *CI* | *Estimates* | *CI* | *Estimates* | *CI* |
| Intercept | 5.64 ^***^ | 5.45 – 5.83 | 1.76 ^***^ | 1.51 – 2.02 | 2.08 ^***^ | 1.82 – 2.34 | 1.57 ^**^ | 0.59 – 2.56 |
| Income |  |  | 0.43 ^***^ | 0.41 – 0.45 | 0.36 ^***^ | 0.33 – 0.38 | 0.36 ^***^ | 0.33 – 0.38 |
| Urban |  |  | -0.08 ^***^ | -0.10 – -0.06 | -0.05 ^***^ | -0.07 – -0.02 | -0.05 ^***^ | -0.07 – -0.02 |
| Household Size |  |  |  |  | 0.25 ^***^ | 0.22 – 0.28 | 0.25 ^***^ | 0.22 – 0.28 |
| Household Structure |  |  |  |  | -0.02 ^*^ | -0.05 – -0.00 | -0.02 ^*^ | -0.05 – -0.00 |
| Cooking Facility |  |  |  |  | -0.00 | -0.03 – 0.02 | -0.00 | -0.03 – 0.02 |
| Infrastructure |  |  |  |  |  |  | -0.02 | -0.09 – 0.06 |
| Travel time to Cities |  |  |  |  |  |  | 0.11 | -0.02 – 0.23 |
| % Working Women in Urban Areas |  |  |  |  |  |  | 3.71 | -0.24 – 7.67 |
| **Random Effects** | | | | | | | | |
| σ^2^ | 0.42 | | 0.36 | | 0.36 | | 0.36 | |
| (τ_2_)^2^ | 0.15 _District:State_ | | 0.14 _District:State_ | | 0.14 _District:State_ | | 0.14 _District:State_ | |
| (τ_1_)^2^ | 0.22 _State_ | | 0.23 _State_ | | 0.23 _State_ | | 0.19 _State_ | |
| ICC | 0.47 | | 0.50 | | 0.51 | | 0.49 | |
| N | 70 _District_ | | 70 _District_ | | 70 _District_ | | 70 _District_ | |
|  | 33 _State_ | | 33 _State_ | | 33 _State_ | | 33 _State_ | |
| Observations | 13726 | | 13726 | | 13726 | | 13726 | |
| Marginal R^2^ / Conditional R^2^ | 0.000 / 0.469 | | 0.081 / 0.544 | | 0.090 / 0.558 | | 0.098 / 0.538 | |
| ** p<0.05   ** p<0.01   *** p<0.001* | | | | | | | | |

Table S45: Multi-level model estimates for (log) household consumption of *Dates* with household- and district-level covariates.

|  | **Dates** | | **Dates** | | **Dates** | | **Dates** | |
| --- | --- | --- | --- | --- | --- | --- | --- | --- |
| *Predictors* | *Estimates* | *CI* | *Estimates* | *CI* | *Estimates* | *CI* | *Estimates* | *CI* |
| Intercept | 5.84 ^***^ | 5.72 – 5.95 | 3.30 ^***^ | 2.99 – 3.61 | 3.64 ^***^ | 3.32 – 3.96 | 2.50 ^***^ | 1.41 – 3.59 |
| Income |  |  | 0.28 ^***^ | 0.25 – 0.31 | 0.21 ^***^ | 0.18 – 0.25 | 0.21 ^***^ | 0.18 – 0.25 |
| Urban |  |  | -0.09 ^***^ | -0.13 – -0.05 | -0.07 ^**^ | -0.11 – -0.02 | -0.07 ^**^ | -0.11 – -0.02 |
| Household Size |  |  |  |  | 0.31 ^***^ | 0.25 – 0.37 | 0.31 ^***^ | 0.25 – 0.37 |
| Household Structure |  |  |  |  | -0.08 ^***^ | -0.12 – -0.04 | -0.08 ^***^ | -0.12 – -0.04 |
| Cooking Facility |  |  |  |  | 0.05 ^*^ | 0.01 – 0.10 | 0.05 ^*^ | 0.01 – 0.10 |
| Infrastructure |  |  |  |  |  |  | 0.10 ^*^ | 0.01 – 0.19 |
| Travel time to Cities |  |  |  |  |  |  | 0.12 | -0.01 – 0.25 |
| % Working Women in Urban Areas |  |  |  |  |  |  | -0.94 | -4.54 – 2.67 |
| **Random Effects** | | | | | | | | |
| σ^2^ | 0.49 | | 0.47 | | 0.46 | | 0.46 | |
| (τ_2_)^2^ | 0.20 _District:State_ | | 0.20 _District:State_ | | 0.20 _District:State_ | | 0.20 _District:State_ | |
| (τ_1_)^2^ | 0.06 _State_ | | 0.05 _State_ | | 0.05 _State_ | | 0.04 _State_ | |
| ICC | 0.35 | | 0.35 | | 0.35 | | 0.34 | |
| N | 65 _District_ | | 65 _District_ | | 65 _District_ | | 65 _District_ | |
|  | 31 _State_ | | 31 _State_ | | 31 _State_ | | 31 _State_ | |
| Observations | 5588 | | 5588 | | 5588 | | 5588 | |
| Marginal R^2^ / Conditional R^2^ | 0.000 / 0.348 | | 0.041 / 0.379 | | 0.054 / 0.384 | | 0.060 / 0.384 | |
| ** p<0.05   ** p<0.01   *** p<0.001* | | | | | | | | |

Table S46: Multi-level model estimates for (log) household consumption of *Cashewnut* with household- and district-level covariates.

|  | **Cashewnut** | | **Cashewnut** | | **Cashewnut** | | **Cashewnut** | |
| --- | --- | --- | --- | --- | --- | --- | --- | --- |
| *Predictors* | *Estimates* | *CI* | *Estimates* | *CI* | *Estimates* | *CI* | *Estimates* | *CI* |
| Intercept | 4.95 ^***^ | 4.79 – 5.10 | 0.25 | -0.05 – 0.55 | 0.29 | -0.01 – 0.60 | 0.07 | -0.66 – 0.80 |
| Income |  |  | 0.49 ^***^ | 0.46 – 0.52 | 0.49 ^***^ | 0.46 – 0.52 | 0.49 ^***^ | 0.46 – 0.52 |
| Urban |  |  | 0.05 ^**^ | 0.01 – 0.09 | 0.01 | -0.03 – 0.05 | 0.01 | -0.03 – 0.05 |
| Household Size |  |  |  |  | -0.03 | -0.08 – 0.02 | -0.03 | -0.08 – 0.02 |
| Household Structure |  |  |  |  | -0.01 | -0.04 – 0.03 | -0.01 | -0.04 – 0.03 |
| Cooking Facility |  |  |  |  | 0.12 ^***^ | 0.08 – 0.17 | 0.12 ^***^ | 0.08 – 0.17 |
| Infrastructure |  |  |  |  |  |  | 0.00 | -0.05 – 0.06 |
| Travel time to Cities |  |  |  |  |  |  | 0.04 | -0.04 – 0.13 |
| % Working Women in Urban Areas |  |  |  |  |  |  | -0.76 | -3.12 – 1.59 |
| **Random Effects** | | | | | | | | |
| σ^2^ | 0.58 | | 0.50 | | 0.50 | | 0.50 | |
| (τ_2_)^2^ | 0.09 _District:State_ | | 0.07 _District:State_ | | 0.07 _District:State_ | | 0.07 _District:State_ | |
| (τ_1_)^2^ | 0.18 _State_ | | 0.14 _State_ | | 0.14 _State_ | | 0.13 _State_ | |
| ICC | 0.32 | | 0.30 | | 0.30 | | 0.29 | |
| N | 70 _District_ | | 70 _District_ | | 70 _District_ | | 70 _District_ | |
|  | 34 _State_ | | 34 _State_ | | 34 _State_ | | 34 _State_ | |
| Observations | 7626 | | 7626 | | 7626 | | 7626 | |
| Marginal R^2^ / Conditional R^2^ | 0.000 / 0.319 | | 0.124 / 0.382 | | 0.127 / 0.385 | | 0.125 / 0.380 | |
| ** p<0.05   ** p<0.01   *** p<0.001* | | | | | | | | |

Table S47: Multi-level model estimates for (log) household consumption of *Other nuts* with household- and district-level covariates.

|  | **Other Nuts** | | **Other Nuts** | | **Other Nuts** | | **Other Nuts** | |
| --- | --- | --- | --- | --- | --- | --- | --- | --- |
| *Predictors* | *Estimates* | *CI* | *Estimates* | *CI* | *Estimates* | *CI* | *Estimates* | *CI* |
| Intercept | 5.28 ^***^ | 5.12 – 5.44 | 1.30 ^***^ | 0.81 – 1.80 | 1.26 ^***^ | 0.74 – 1.77 | 1.18 | -0.16 – 2.52 |
| Income |  |  | 0.43 ^***^ | 0.38 – 0.48 | 0.42 ^***^ | 0.36 – 0.47 | 0.42 ^***^ | 0.37 – 0.48 |
| Urban |  |  | -0.12 ^***^ | -0.18 – -0.05 | -0.13 ^***^ | -0.21 – -0.06 | -0.13 ^***^ | -0.21 – -0.06 |
| Household Size |  |  |  |  | -0.03 | -0.11 – 0.06 | -0.03 | -0.11 – 0.06 |
| Household Structure |  |  |  |  | 0.05 | -0.01 – 0.11 | 0.05 | -0.01 – 0.11 |
| Cooking Facility |  |  |  |  | 0.04 | -0.04 – 0.12 | 0.04 | -0.04 – 0.12 |
| Infrastructure |  |  |  |  |  |  | -0.02 | -0.13 – 0.08 |
| Travel time to Cities |  |  |  |  |  |  | 0.04 | -0.11 – 0.19 |
| % Working Women in Urban Areas |  |  |  |  |  |  | 0.99 | -3.32 – 5.29 |
| **Random Effects** | | | | | | | | |
| σ^2^ | 0.44 | | 0.39 | | 0.39 | | 0.39 | |
| (τ_2_)^2^ | 0.19 _District:State_ | | 0.16 _District:State_ | | 0.16 _District:State_ | | 0.17 _District:State_ | |
| (τ_1_)^2^ | 0.14 _State_ | | 0.14 _State_ | | 0.14 _State_ | | 0.13 _State_ | |
| ICC | 0.43 | | 0.43 | | 0.43 | | 0.43 | |
| N | 51 _District_ | | 51 _District_ | | 51 _District_ | | 51 _District_ | |
|  | 33 _State_ | | 33 _State_ | | 33 _State_ | | 33 _State_ | |
| Observations | 2209 | | 2209 | | 2209 | | 2209 | |
| Marginal R^2^ / Conditional R^2^ | 0.000 / 0.429 | | 0.091 / 0.483 | | 0.093 / 0.486 | | 0.091 / 0.484 | |
| ** p<0.05   ** p<0.01   *** p<0.001* | | | | | | | | |

Table S48: Multi-level model estimates for (log) household consumption of *Raisins* with household- and district-level covariates.

|  | **Raisins** | | **Raisins** | | **Raisins** | | **Raisins** | |
| --- | --- | --- | --- | --- | --- | --- | --- | --- |
| *Predictors* | *Estimates* | *CI* | *Estimates* | *CI* | *Estimates* | *CI* | *Estimates* | *CI* |
| Intercept | 4.85 ^***^ | 4.71 – 4.98 | 0.77 ^***^ | 0.53 – 1.02 | 0.86 ^***^ | 0.60 – 1.11 | 0.84 ^**^ | 0.20 – 1.47 |
| Income |  |  | 0.43 ^***^ | 0.41 – 0.46 | 0.42 ^***^ | 0.40 – 0.45 | 0.42 ^***^ | 0.40 – 0.45 |
| Urban |  |  | 0.02 | -0.01 – 0.06 | -0.01 | -0.04 – 0.02 | -0.01 | -0.04 – 0.03 |
| Household Size |  |  |  |  | 0.00 | -0.04 – 0.04 | 0.00 | -0.04 – 0.04 |
| Household Structure |  |  |  |  | -0.01 | -0.04 – 0.02 | -0.01 | -0.04 – 0.02 |
| Cooking Facility |  |  |  |  | 0.08 ^***^ | 0.04 – 0.12 | 0.08 ^***^ | 0.04 – 0.12 |
| Infrastructure |  |  |  |  |  |  | -0.01 | -0.06 – 0.04 |
| Travel time to Cities |  |  |  |  |  |  | 0.02 | -0.06 – 0.09 |
| % Working Women in Urban Areas |  |  |  |  |  |  | -0.91 | -3.08 – 1.26 |
| **Random Effects** | | | | | | | | |
| σ^2^ | 0.48 | | 0.42 | | 0.42 | | 0.42 | |
| (τ_2_)^2^ | 0.07 _District:State_ | | 0.07 _District:State_ | | 0.07 _District:State_ | | 0.07 _District:State_ | |
| (τ_1_)^2^ | 0.14 _State_ | | 0.12 _State_ | | 0.12 _State_ | | 0.12 _State_ | |
| ICC | 0.31 | | 0.31 | | 0.31 | | 0.31 | |
| N | 71 _District_ | | 71 _District_ | | 71 _District_ | | 71 _District_ | |
|  | 35 _State_ | | 35 _State_ | | 35 _State_ | | 35 _State_ | |
| Observations | 9566 | | 9566 | | 9566 | | 9566 | |
| Marginal R^2^ / Conditional R^2^ | 0.000 / 0.305 | | 0.117 / 0.390 | | 0.119 / 0.393 | | 0.115 / 0.386 | |
| ** p<0.05   ** p<0.01   *** p<0.001* | | | | | | | | |

Table S49: Multi-level model estimates for (log) household consumption of *Other dry fruits* with household- and district-level covariates.

|  | **Other Dry Fruits** | | **Other Dry Fruits** | | **Other Dry Fruits** | | **Other Dry Fruits** | |
| --- | --- | --- | --- | --- | --- | --- | --- | --- |
| *Predictors* | *Estimates* | *CI* | *Estimates* | *CI* | *Estimates* | *CI* | *Estimates* | *CI* |
| Intercept | 5.21 ^***^ | 4.99 – 5.43 | 1.78 ^***^ | 1.37 – 2.20 | 1.75 ^***^ | 1.32 – 2.18 | 1.67 ^**^ | 0.64 – 2.70 |
| Income |  |  | 0.37 ^***^ | 0.33 – 0.41 | 0.37 ^***^ | 0.33 – 0.41 | 0.37 ^***^ | 0.33 – 0.41 |
| Urban |  |  | -0.03 | -0.08 – 0.02 | -0.01 | -0.07 – 0.04 | -0.01 | -0.06 – 0.05 |
| Household Size |  |  |  |  | 0.02 | -0.05 – 0.09 | 0.02 | -0.05 – 0.09 |
| Household Structure |  |  |  |  | 0.01 | -0.04 – 0.06 | 0.01 | -0.04 – 0.06 |
| Cooking Facility |  |  |  |  | -0.04 | -0.10 – 0.02 | -0.04 | -0.09 – 0.02 |
| Infrastructure |  |  |  |  |  |  | -0.06 | -0.13 – 0.02 |
| Travel time to Cities |  |  |  |  |  |  | 0.07 | -0.05 – 0.19 |
| % Working Women in Urban Areas |  |  |  |  |  |  | 1.50 | -1.88 – 4.88 |
| **Random Effects** | | | | | | | | |
| σ^2^ | 0.60 | | 0.56 | | 0.56 | | 0.56 | |
| (τ2)^2^ | 0.16 _District:State_ | | 0.15 _District:State_ | | 0.16 _District:State_ | | 0.16 _District:State_ | |
| (τ1)^2^ | 0.38 _State_ | | 0.36 _State_ | | 0.36 _State_ | | 0.30 _State_ | |
| ICC | 0.47 | | 0.48 | | 0.48 | | 0.45 | |
| N | 71 _District_ | | 71 _District_ | | 71 _District_ | | 71 _District_ | |
|  | 35 _State_ | | 35 _State_ | | 35 _State_ | | 35 _State_ | |
| Observations | 4931 | | 4931 | | 4931 | | 4931 | |
| Marginal R^2^ / Conditional R^2^ | 0.000 / 0.472 | | 0.051 / 0.505 | | 0.051 / 0.504 | | 0.058 / 0.481 | |
| ** p<0.05   ** p<0.01   *** p<0.001* | | | | | | | | |

Table S50: Multi-level model estimates for (log) household consumption of *Ginger* with household- and district-level covariates.

|  | **Ginger** | | **Ginger** | | **Ginger** | | **Ginger** | |
| --- | --- | --- | --- | --- | --- | --- | --- | --- |
| *Predictors* | *Estimates* | *CI* | *Estimates* | *CI* | *Estimates* | *CI* | *Estimates* | *CI* |
| Intercept | 5.36 ^***^ | 5.23 – 5.49 | 2.14 ^***^ | 2.00 – 2.28 | 2.43 ^***^ | 2.29 – 2.57 | 2.47 ^***^ | 1.92 – 3.02 |
| Income |  |  | 0.36 ^***^ | 0.36 – 0.37 | 0.28 ^***^ | 0.27 – 0.29 | 0.28 ^***^ | 0.27 – 0.29 |
| Urban |  |  | -0.05 ^***^ | -0.06 – -0.04 | -0.01 ^*^ | -0.02 – -0.00 | -0.01 ^*^ | -0.02 – -0.00 |
| Household Size |  |  |  |  | 0.29 ^***^ | 0.28 – 0.30 | 0.29 ^***^ | 0.28 – 0.30 |
| Household Structure |  |  |  |  | 0.00 | -0.01 – 0.01 | 0.00 | -0.01 – 0.01 |
| Cooking Facility |  |  |  |  | -0.01 ^*^ | -0.02 – -0.00 | -0.01 ^*^ | -0.02 – -0.00 |
| Infrastructure |  |  |  |  |  |  | 0.00 | -0.04 – 0.04 |
| Travel time to Cities |  |  |  |  |  |  | -0.01 | -0.09 – 0.06 |
| % Working Women in Urban Areas |  |  |  |  |  |  | 0.27 | -1.67 – 2.20 |
| **Random Effects** | | | | | | | | |
| σ^2^ | 0.38 | | 0.34 | | 0.33 | | 0.33 | |
| (τ_2_)^2^ | 0.09 _District:State_ | | 0.09 _District:State_ | | 0.09 _District:State_ | | 0.09 _District:State_ | |
| (τ_1_)^2^ | 0.14 _State_ | | 0.12 _State_ | | 0.12 _State_ | | 0.12 _State_ | |
| ICC | 0.38 | | 0.39 | | 0.39 | | 0.40 | |
| N | 71 _District_ | | 71 _District_ | | 71 _District_ | | 71 _District_ | |
|  | 35 _State_ | | 35 _State_ | | 35 _State_ | | 35 _State_ | |
| Observations | 77065 | | 77065 | | 77065 | | 77065 | |
| Marginal R^2^ / Conditional R^2^ | 0.000 / 0.376 | | 0.083 / 0.436 | | 0.100 / 0.453 | | 0.100 / 0.460 | |
| ** p<0.05   ** p<0.01   *** p<0.001* | | | | | | | | |

Table S51: Multi-level model estimates for (log) household consumption of *Cumin* with household- and district-level covariates.

|  | **Cumin** | | **Cumin** | | **Cumin** | | **Cumin** | |
| --- | --- | --- | --- | --- | --- | --- | --- | --- |
| *Predictors* | *Estimates* | *CI* | *Estimates* | *CI* | *Estimates* | *CI* | *Estimates* | *CI* |
| Intercept | 4.32 ^***^ | 4.24 – 4.41 | 1.49 ^***^ | 1.39 – 1.59 | 1.81 ^***^ | 1.71 – 1.92 | 1.59 ^***^ | 1.20 – 1.97 |
| Income |  |  | 0.32 ^***^ | 0.31 – 0.33 | 0.23 ^***^ | 0.22 – 0.24 | 0.23 ^***^ | 0.22 – 0.24 |
| Urban |  |  | -0.03 ^***^ | -0.04 – -0.03 | -0.00 | -0.01 – 0.01 | -0.00 | -0.01 – 0.01 |
| Household Size |  |  |  |  | 0.29 ^***^ | 0.27 – 0.30 | 0.29 ^***^ | 0.27 – 0.30 |
| Household Structure |  |  |  |  | 0.00 | -0.00 – 0.01 | 0.00 | -0.00 – 0.01 |
| Cooking Facility |  |  |  |  | 0.01 | -0.00 – 0.02 | 0.01 | -0.00 – 0.02 |
| Infrastructure |  |  |  |  |  |  | 0.03 ^*^ | 0.00 – 0.06 |
| Travel time to Cities |  |  |  |  |  |  | 0.02 | -0.03 – 0.07 |
| % Working Women in Urban Areas |  |  |  |  |  |  | -0.62 | -1.96 – 0.72 |
| **Random Effects** | | | | | | | | |
| σ^2^ | 0.33 | | 0.30 | | 0.28 | | 0.28 | |
| (τ_2_)^2^ | 0.04 _District:State_ | | 0.04 _District:State_ | | 0.04 _District:State_ | | 0.04 _District:State_ | |
| (τ_1_)^2^ | 0.05 _State_ | | 0.05 _State_ | | 0.05 _State_ | | 0.05 _State_ | |
| ICC | 0.23 | | 0.25 | | 0.25 | | 0.25 | |
| N | 71 _District_ | | 71 _District_ | | 71 _District_ | | 71 _District_ | |
|  | 35 _State_ | | 35 _State_ | | 35 _State_ | | 35 _State_ | |
| Observations | 79307 | | 79307 | | 79307 | | 79307 | |
| Marginal R^2^ / Conditional R^2^ | 0.000 / 0.226 | | 0.092 / 0.316 | | 0.114 / 0.339 | | 0.118 / 0.335 | |
| ** p<0.05   ** p<0.01   *** p<0.001* | | | | | | | | |

Table S52: Multi-level model estimates for (log) household consumption of *Black pepper* with household- and district-level covariates.

|  | **Black Pepper** | | **Black Pepper** | | **Black Pepper** | | **Black Pepper** | |
| --- | --- | --- | --- | --- | --- | --- | --- | --- |
| *Predictors* | *Estimates* | *CI* | *Estimates* | *CI* | *Estimates* | *CI* | *Estimates* | *CI* |
| Intercept | 3.87 ^***^ | 3.78 – 3.96 | 1.49 ^***^ | 1.35 – 1.62 | 1.78 ^***^ | 1.64 – 1.92 | 1.87 ^***^ | 1.27 – 2.48 |
| Income |  |  | 0.26 ^***^ | 0.25 – 0.28 | 0.19 ^***^ | 0.18 – 0.21 | 0.19 ^***^ | 0.18 – 0.21 |
| Urban |  |  | -0.03 ^***^ | -0.05 – -0.02 | -0.01 | -0.03 – 0.00 | -0.01 | -0.03 – 0.00 |
| Household Size |  |  |  |  | 0.26 ^***^ | 0.24 – 0.28 | 0.26 ^***^ | 0.24 – 0.28 |
| Household Structure |  |  |  |  | -0.02 ^*^ | -0.03 – -0.00 | -0.02 ^*^ | -0.03 – -0.00 |
| Cooking Facility |  |  |  |  | 0.02 ^*^ | 0.00 – 0.04 | 0.02 ^*^ | 0.00 – 0.04 |
| Infrastructure |  |  |  |  |  |  | -0.03 | -0.08 – 0.02 |
| Travel time to Cities |  |  |  |  |  |  | 0.01 | -0.07 – 0.08 |
| % Working Women in Urban Areas |  |  |  |  |  |  | 2.23 ^*^ | 0.04 – 4.43 |
| **Random Effects** | | | | | | | | |
| σ^2^ | 0.40 | | 0.38 | | 0.37 | | 0.37 | |
| (τ_2_)^2^ | 0.11 _District:State_ | | 0.11 _District:State_ | | 0.11 _District:State_ | | 0.11 _District:State_ | |
| (τ_1_)^2^ | 0.06 _State_ | | 0.05 _State_ | | 0.05 _State_ | | 0.04 _State_ | |
| ICC | 0.30 | | 0.30 | | 0.30 | | 0.29 | |
| N | 71 _District_ | | 71 _District_ | | 71 _District_ | | 71 _District_ | |
|  | 35 _State_ | | 35 _State_ | | 35 _State_ | | 35 _State_ | |
| Observations | 31998 | | 31998 | | 31998 | | 31998 | |
| Marginal R^2^ / Conditional R^2^ | 0.000 / 0.298 | | 0.050 / 0.333 | | 0.063 / 0.344 | | 0.065 / 0.338 | |
| ** p<0.05   ** p<0.01   *** p<0.001* | | | | | | | | |

Table S53: Multi-level model estimates for (log) household consumption of *Tamarind* with household- and district-level covariates.

|  | **Tamarind** | | **Tamarind** | | **Tamarind** | | **Tamarind** | |
| --- | --- | --- | --- | --- | --- | --- | --- | --- |
| *Predictors* | *Estimates* | *CI* | *Estimates* | *CI* | *Estimates* | *CI* | *Estimates* | *CI* |
| Intercept | 4.88 ^***^ | 4.70 – 5.07 | 2.57 ^***^ | 2.35 – 2.79 | 2.70 ^***^ | 2.48 – 2.93 | 3.06 ^***^ | 2.11 – 4.02 |
| Income |  |  | 0.26 ^***^ | 0.25 – 0.27 | 0.18 ^***^ | 0.17 – 0.20 | 0.18 ^***^ | 0.17 – 0.20 |
| Urban |  |  | -0.08 ^***^ | -0.10 – -0.06 | -0.04 ^***^ | -0.06 – -0.02 | -0.04 ^***^ | -0.06 – -0.02 |
| Household Size |  |  |  |  | 0.33 ^***^ | 0.30 – 0.35 | 0.33 ^***^ | 0.30 – 0.35 |
| Household Structure |  |  |  |  | 0.03 ^***^ | 0.02 – 0.05 | 0.03 ^***^ | 0.02 – 0.05 |
| Cooking Facility |  |  |  |  | -0.04 ^***^ | -0.06 – -0.02 | -0.04 ^***^ | -0.06 – -0.02 |
| Infrastructure |  |  |  |  |  |  | -0.02 | -0.10 – 0.05 |
| Travel time to Cities |  |  |  |  |  |  | -0.03 | -0.16 – 0.09 |
| % Working Women in Urban Areas |  |  |  |  |  |  | -2.15 | -5.20 – 0.90 |
| **Random Effects** | | | | | | | | |
| σ^2^ | 0.42 | | 0.39 | | 0.37 | | 0.37 | |
| (τ_2_)^2^ | 0.17 _District:State_ | | 0.17 _District:State_ | | 0.17 _District:State_ | | 0.17 _District:State_ | |
| (τ_1_)^2^ | 0.26 _State_ | | 0.25 _State_ | | 0.28 _State_ | | 0.30 _State_ | |
| ICC | 0.51 | | 0.52 | | 0.54 | | 0.55 | |
| N | 59 _District_ | | 59 _District_ | | 59 _District_ | | 59 _District_ | |
|  | 35 _State_ | | 35 _State_ | | 35 _State_ | | 35 _State_ | |
| Observations | 25883 | | 25883 | | 25883 | | 25883 | |
| Marginal R^2^ / Conditional R^2^ | 0.000 / 0.506 | | 0.032 / 0.533 | | 0.053 / 0.569 | | 0.056 / 0.580 | |
| ** p<0.05   ** p<0.01   *** p<0.001* | | | | | | | | |

Table S54: Multi-level model estimates for (log) household consumption of *Curry powder* with household- and district-level covariates.

|  | **Curry Powder** | | **Curry Powder** | | **Curry Powder** | | **Curry Powder** | |
| --- | --- | --- | --- | --- | --- | --- | --- | --- |
| *Predictors* | *Estimates* | *CI* | *Estimates* | *CI* | *Estimates* | *CI* | *Estimates* | *CI* |
| Intercept | 4.32 ^***^ | 4.21 – 4.44 | 1.91 ^***^ | 1.76 – 2.06 | 2.14 ^***^ | 1.98 – 2.30 | 1.54 ^***^ | 0.87 – 2.21 |
| Income |  |  | 0.27 ^***^ | 0.26 – 0.28 | 0.20 ^***^ | 0.19 – 0.22 | 0.20 ^***^ | 0.19 – 0.22 |
| Urban |  |  | -0.02 ^*^ | -0.04 – -0.00 | 0.01 | -0.01 – 0.03 | 0.01 | -0.01 – 0.03 |
| Household Size |  |  |  |  | 0.25 ^***^ | 0.23 – 0.28 | 0.25 ^***^ | 0.23 – 0.28 |
| Household Structure |  |  |  |  | -0.01 | -0.02 – 0.01 | -0.01 | -0.02 – 0.01 |
| Cooking Facility |  |  |  |  | 0.00 | -0.02 – 0.02 | 0.00 | -0.02 – 0.02 |
| Infrastructure |  |  |  |  |  |  | 0.01 | -0.04 – 0.06 |
| Travel time to Cities |  |  |  |  |  |  | 0.10 ^*^ | 0.02 – 0.19 |
| % Working Women in Urban Areas |  |  |  |  |  |  | 1.61 | -0.72 – 3.95 |
| **Random Effects** | | | | | | | | |
| σ^2^ | 0.44 | | 0.42 | | 0.41 | | 0.41 | |
| (τ_2_)^2^ | 0.13 _District:State_ | | 0.13 _District:State_ | | 0.13 _District:State_ | | 0.13 _District:State_ | |
| (τ_1_)^2^ | 0.10 _State_ | | 0.07 _State_ | | 0.07 _State_ | | 0.08 _State_ | |
| ICC | 0.34 | | 0.32 | | 0.34 | | 0.34 | |
| N | 70 _District_ | | 70 _District_ | | 70 _District_ | | 70 _District_ | |
|  | 35 _State_ | | 35 _State_ | | 35 _State_ | | 35 _State_ | |
| Observations | 32443 | | 32443 | | 32443 | | 32443 | |
| Marginal R^2^ / Conditional R^2^ | 0.000 / 0.337 | | 0.044 / 0.355 | | 0.055 / 0.373 | | 0.060 / 0.380 | |
| ** p<0.05   ** p<0.01   *** p<0.001* | | | | | | | | |

Table S55: Multi-level model estimates for (log) household consumption of *Tea (cups)* with household- and district-level covariates.

|  | **Tea (cups)** | | **Tea (cups)** | | **Tea (cups)** | | **Tea (cups)** | |
| --- | --- | --- | --- | --- | --- | --- | --- | --- |
| *Predictors* | *Estimates* | *CI* | *Estimates* | *CI* | *Estimates* | *CI* | *Estimates* | *CI* |
| Intercept | 2.88 ^***^ | 2.78 – 2.98 | -0.41 ^***^ | -0.56 – -0.26 | -0.30 ^***^ | -0.46 – -0.14 | -0.70 ^*^ | -1.29 – -0.10 |
| Income |  |  | 0.36 ^***^ | 0.35 – 0.38 | 0.36 ^***^ | 0.34 – 0.37 | 0.36 ^***^ | 0.34 – 0.37 |
| Urban |  |  | 0.09 ^***^ | 0.08 – 0.11 | 0.10 ^***^ | 0.08 – 0.12 | 0.10 ^***^ | 0.08 – 0.12 |
| Household Size |  |  |  |  | 0.07 ^***^ | 0.05 – 0.09 | 0.07 ^***^ | 0.05 – 0.09 |
| Household Structure |  |  |  |  | -0.06 ^***^ | -0.08 – -0.05 | -0.06 ^***^ | -0.08 – -0.05 |
| Cooking Facility |  |  |  |  | -0.01 | -0.03 – 0.01 | -0.01 | -0.03 – 0.01 |
| Infrastructure |  |  |  |  |  |  | 0.04 | -0.01 – 0.08 |
| Travel time to Cities |  |  |  |  |  |  | 0.03 | -0.05 – 0.11 |
| % Working Women in Urban Areas |  |  |  |  |  |  | 1.27 | -0.81 – 3.35 |
| **Random Effects** | | | | | | | | |
| σ^2^ | 0.59 | | 0.55 | | 0.55 | | 0.55 | |
| (τ_2_)^2^ | 0.12 _District:State_ | | 0.11 _District:State_ | | 0.11 _District:State_ | | 0.11 _District:State_ | |
| (τ_1_)^2^ | 0.07 _State_ | | 0.07 _State_ | | 0.07 _State_ | | 0.07 _State_ | |
| ICC | 0.25 | | 0.25 | | 0.25 | | 0.25 | |
| N | 71 _District_ | | 71 _District_ | | 71 _District_ | | 71 _District_ | |
|  | 35 _State_ | | 35 _State_ | | 35 _State_ | | 35 _State_ | |
| Observations | 44849 | | 44849 | | 44849 | | 44849 | |
| Marginal R^2^ / Conditional R^2^ | 0.000 / 0.247 | | 0.071 / 0.302 | | 0.072 / 0.304 | | 0.081 / 0.310 | |
| ** p<0.05   ** p<0.01   *** p<0.001* | | | | | | | | |

Table S56: Multi-level model estimates for (log) household consumption of *Coffee (cups)* with household- and district-level covariates.

|  | **Coffee (cups)** | | **Coffee (cups)** | | **Coffee (cups)** | | **Coffee (cups)** | |
| --- | --- | --- | --- | --- | --- | --- | --- | --- |
| *Predictors* | *Estimates* | *CI* | *Estimates* | *CI* | *Estimates* | *CI* | *Estimates* | *CI* |
| Intercept | 2.26 ^***^ | 2.14 – 2.39 | -0.63 | -1.45 – 0.19 | -0.56 | -1.41 – 0.29 | -1.78 ^*^ | -3.52 – -0.04 |
| Income |  |  | 0.32 ^***^ | 0.23 – 0.40 | 0.33 ^***^ | 0.23 – 0.43 | 0.33 ^***^ | 0.23 – 0.42 |
| Urban |  |  | -0.12 ^*^ | -0.25 – -0.00 | -0.12 | -0.25 – 0.01 | -0.12 | -0.25 – 0.01 |
| Household Size |  |  |  |  | 0.02 | -0.14 – 0.18 | 0.03 | -0.13 – 0.19 |
| Household Structure |  |  |  |  | -0.08 | -0.19 – 0.03 | -0.09 | -0.20 – 0.02 |
| Cooking Facility |  |  |  |  | -0.02 | -0.16 – 0.11 | -0.02 | -0.16 – 0.11 |
| Infrastructure |  |  |  |  |  |  | 0.11 | -0.01 – 0.22 |
| Travel time to Cities |  |  |  |  |  |  | 0.12 | -0.07 – 0.32 |
| % Working Women in Urban Areas |  |  |  |  |  |  | 0.13 | -3.90 – 4.16 |
| **Random Effects** | | | | | | | | |
| σ^2^ | 0.69 | | 0.67 | | 0.67 | | 0.67 | |
| (τ_2_)^2^ | 0.23 _District:State_ | | 0.20 _District:State_ | | 0.20 _District:State_ | | 0.21 _District:State_ | |
| (τ_1_)^2^ | 0.05 _State_ | | 0.04 _State_ | | 0.04 _State_ | | 0.03 _State_ | |
| ICC | 0.29 | | 0.26 | | 0.26 | | 0.26 | |
| N | 41 _District_ | | 41 _District_ | | 41 _District_ | | 41 _District_ | |
|  | 33 _State_ | | 33 _State_ | | 33 _State_ | | 33 _State_ | |
| Observations | 1177 | | 1177 | | 1177 | | 1177 | |
| Marginal R^2^ / Conditional R^2^ | 0.000 / 0.290 | | 0.044 / 0.295 | | 0.047 / 0.299 | | 0.053 / 0.298 | |
| ** p<0.05   ** p<0.01   *** p<0.001* | | | | | | | | |

Table S57: Multi-level model estimates for (log) household consumption of *Coffee (powder)* with household- and district-level covariates.

|  | **Coffee (powder)** | | **Coffee (powder)** | | **Coffee (powder)** | | **Coffee (powder)** | |
| --- | --- | --- | --- | --- | --- | --- | --- | --- |
| *Predictors* | *Estimates* | *CI* | *Estimates* | *CI* | *Estimates* | *CI* | *Estimates* | *CI* |
| Intercept | 4.10 ^***^ | 3.93 – 4.27 | 1.49 ^***^ | 1.14 – 1.83 | 1.59 ^***^ | 1.24 – 1.94 | 1.13 | -0.06 – 2.32 |
| Income |  |  | 0.28 ^***^ | 0.25 – 0.31 | 0.25 ^***^ | 0.22 – 0.28 | 0.25 ^***^ | 0.22 – 0.28 |
| Urban |  |  | -0.06 ^**^ | -0.10 – -0.01 | -0.04 | -0.08 – 0.00 | -0.04 | -0.09 – 0.00 |
| Household Size |  |  |  |  | 0.15 ^***^ | 0.09 – 0.21 | 0.15 ^***^ | 0.09 – 0.21 |
| Household Structure |  |  |  |  | -0.02 | -0.06 – 0.02 | -0.02 | -0.06 – 0.02 |
| Cooking Facility |  |  |  |  | 0.00 | -0.05 – 0.05 | 0.00 | -0.05 – 0.05 |
| Infrastructure |  |  |  |  |  |  | 0.04 | -0.07 – 0.14 |
| Travel time to Cities |  |  |  |  |  |  | 0.05 | -0.09 – 0.19 |
| % Working Women in Urban Areas |  |  |  |  |  |  | 0.39 | -2.91 – 3.70 |
| **Random Effects** | | | | | | | | |
| σ^2^ | 0.61 | | 0.59 | | 0.58 | | 0.58 | |
| (τ_2_)^2^ | 0.15 _District:State_ | | 0.15 _District:State_ | | 0.15 _District:State_ | | 0.15 _District:State_ | |
| (τ_1_)^2^ | 0.21 _State_ | | 0.22 _State_ | | 0.22 _State_ | | 0.22 _State_ | |
| ICC | 0.37 | | 0.39 | | 0.39 | | 0.39 | |
| N | 59 _District_ | | 59 _District_ | | 59 _District_ | | 59 _District_ | |
|  | 34 _State_ | | 34 _State_ | | 34 _State_ | | 34 _State_ | |
| Observations | 6927 | | 6927 | | 6927 | | 6927 | |
| Marginal R^2^ / Conditional R^2^ | 0.000 / 0.366 | | 0.035 / 0.411 | | 0.037 / 0.412 | | 0.037 / 0.414 | |
| ** p<0.05   ** p<0.01   *** p<0.001* | | | | | | | | |

Table S58: Multi-level model estimates for (log) household consumption of *Mineral water* with household- and district-level covariates.

|  | **Mineral Water** | | **Mineral Water** | | **Mineral Water** | | **Mineral Water** | |
| --- | --- | --- | --- | --- | --- | --- | --- | --- |
| *Predictors* | *Estimates* | *CI* | *Estimates* | *CI* | *Estimates* | *CI* | *Estimates* | *CI* |
| Intercept | 8.28 ^***^ | 7.93 – 8.64 | 2.22 ^***^ | 1.22 – 3.22 | 2.05 ^***^ | 1.02 – 3.07 | 2.20 ^*^ | 0.01 – 4.38 |
| Income |  |  | 0.64 ^***^ | 0.54 – 0.74 | 0.68 ^***^ | 0.58 – 0.79 | 0.68 ^***^ | 0.57 – 0.78 |
| Urban |  |  | -0.04 | -0.17 – 0.10 | -0.09 | -0.23 – 0.05 | -0.10 | -0.24 – 0.04 |
| Household Size |  |  |  |  | -0.30 ^**^ | -0.49 – -0.12 | -0.30 ^**^ | -0.49 – -0.11 |
| Household Structure |  |  |  |  | 0.07 | -0.05 – 0.18 | 0.06 | -0.06 – 0.18 |
| Cooking Facility |  |  |  |  | 0.07 | -0.10 – 0.23 | 0.07 | -0.10 – 0.23 |
| Infrastructure |  |  |  |  |  |  | 0.12 | -0.04 – 0.27 |
| Travel time to Cities |  |  |  |  |  |  | -0.14 | -0.40 – 0.12 |
| % Working Women in Urban Areas |  |  |  |  |  |  | -3.65 | -10.20 – 2.90 |
| **Random Effects** | | | | | | | | |
| σ^2^ | 1.65 | | 1.53 | | 1.53 | | 1.53 | |
| (τ_2_)^2^ | 0.49 _District:State_ | | 0.43 _District:State_ | | 0.42 _District:State_ | | 0.41 _District:State_ | |
| (τ_1_)^2^ | 0.89 _State_ | | 0.93 _State_ | | 0.90 _State_ | | 0.81 _State_ | |
| ICC | 0.46 | | 0.47 | | 0.46 | | 0.44 | |
| N | 58 _District_ | | 58 _District_ | | 58 _District_ | | 58 _District_ | |
|  | 34 _State_ | | 34 _State_ | | 34 _State_ | | 34 _State_ | |
| Observations | 2015 | | 2015 | | 2015 | | 2015 | |
| Marginal R^2^ / Conditional R^2^ | 0.000 / 0.457 | | 0.057 / 0.501 | | 0.062 / 0.497 | | 0.088 / 0.492 | |
| ** p<0.05   ** p<0.01   *** p<0.001* | | | | | | | | |

Table S59: Multi-level model estimates for (log) household consumption of *Cold beverage* with household- and district-level covariates.

|  | **Cold Beverage** | | **Cold Beverage** | | **Cold Beverage** | | **Cold Beverage** | |
| --- | --- | --- | --- | --- | --- | --- | --- | --- |
| *Predictors* | *Estimates* | *CI* | *Estimates* | *CI* | *Estimates* | *CI* | *Estimates* | *CI* |
| Intercept | 7.18 ^***^ | 7.08 – 7.28 | 2.21 ^***^ | 1.92 – 2.50 | 2.34 ^***^ | 2.04 – 2.65 | 2.31 ^***^ | 1.61 – 3.02 |
| Income |  |  | 0.54 ^***^ | 0.51 – 0.57 | 0.52 ^***^ | 0.49 – 0.55 | 0.52 ^***^ | 0.49 – 0.55 |
| Urban |  |  | -0.03 | -0.07 – 0.01 | -0.06 ^**^ | -0.11 – -0.02 | -0.06 ^**^ | -0.10 – -0.02 |
| Household Size |  |  |  |  | 0.02 | -0.03 – 0.08 | 0.02 | -0.03 – 0.08 |
| Household Structure |  |  |  |  | -0.02 | -0.06 – 0.02 | -0.02 | -0.06 – 0.02 |
| Cooking Facility |  |  |  |  | 0.10 ^***^ | 0.05 – 0.14 | 0.10 ^***^ | 0.05 – 0.14 |
| Infrastructure |  |  |  |  |  |  | 0.01 | -0.04 – 0.06 |
| Travel time to Cities |  |  |  |  |  |  | -0.00 | -0.08 – 0.08 |
| % Working Women in Urban Areas |  |  |  |  |  |  | -0.80 | -3.05 – 1.44 |
| **Random Effects** | | | | | | | | |
| σ^2^ | 0.53 | | 0.44 | | 0.44 | | 0.44 | |
| (τ_2_)^2^ | 0.10 _District:State_ | | 0.07 _District:State_ | | 0.07 _District:State_ | | 0.07 _District:State_ | |
| (τ_1_)^2^ | 0.07 _State_ | | 0.06 _State_ | | 0.06 _State_ | | 0.06 _State_ | |
| ICC | 0.24 | | 0.23 | | 0.23 | | 0.23 | |
| N | 70 _District_ | | 70 _District_ | | 70 _District_ | | 70 _District_ | |
|  | 35 _State_ | | 35 _State_ | | 35 _State_ | | 35 _State_ | |
| Observations | 6514 | | 6514 | | 6514 | | 6514 | |
| Marginal R^2^ / Conditional R^2^ | 0.000 / 0.242 | | 0.160 / 0.353 | | 0.163 / 0.354 | | 0.163 / 0.355 | |
| ** p<0.05   ** p<0.01   *** p<0.001* | | | | | | | | |

Table S60: Multi-level model estimates for (log) household consumption of *Fruit juice* with household- and district-level covariates.

|  | **Fruit Juice** | | **Fruit Juice** | | **Fruit Juice** | | **Fruit Juice** | |
| --- | --- | --- | --- | --- | --- | --- | --- | --- |
| *Predictors* | *Estimates* | *CI* | *Estimates* | *CI* | *Estimates* | *CI* | *Estimates* | *CI* |
| Intercept | 6.94 ^***^ | 6.84 – 7.04 | 2.29 ^***^ | 1.94 – 2.64 | 2.42 ^***^ | 2.06 – 2.79 | 2.08 ^***^ | 1.26 – 2.89 |
| Income |  |  | 0.50 ^***^ | 0.46 – 0.54 | 0.49 ^***^ | 0.45 – 0.53 | 0.49 ^***^ | 0.45 – 0.53 |
| Urban |  |  | -0.03 | -0.07 – 0.02 | -0.06 ^*^ | -0.11 – -0.00 | -0.05 ^*^ | -0.11 – -0.00 |
| Household Size |  |  |  |  | 0.01 | -0.05 – 0.08 | 0.02 | -0.05 – 0.08 |
| Household Structure |  |  |  |  | -0.05 ^*^ | -0.09 – -0.01 | -0.05 ^*^ | -0.10 – -0.01 |
| Cooking Facility |  |  |  |  | 0.08 ^**^ | 0.02 – 0.14 | 0.08 ^**^ | 0.02 – 0.14 |
| Infrastructure |  |  |  |  |  |  | 0.01 | -0.05 – 0.07 |
| Travel time to Cities |  |  |  |  |  |  | 0.05 | -0.04 – 0.15 |
| % Working Women in Urban Areas |  |  |  |  |  |  | 0.84 | -1.68 – 3.37 |
| **Random Effects** | | | | | | | | |
| σ^2^ | 0.58 | | 0.51 | | 0.51 | | 0.51 | |
| (τ_2_)^2^ | 0.13 _District:State_ | | 0.12 _District:State_ | | 0.12 _District:State_ | | 0.12 _District:State_ | |
| (τ_1_)^2^ | 0.06 _State_ | | 0.04 _State_ | | 0.04 _State_ | | 0.04 _State_ | |
| ICC | 0.25 | | 0.24 | | 0.23 | | 0.23 | |
| N | 67 _District_ | | 67 _District_ | | 67 _District_ | | 67 _District_ | |
|  | 34 _State_ | | 34 _State_ | | 34 _State_ | | 34 _State_ | |
| Observations | 4874 | | 4874 | | 4874 | | 4874 | |
| Marginal R^2^ / Conditional R^2^ | 0.000 / 0.249 | | 0.127 / 0.333 | | 0.131 / 0.334 | | 0.133 / 0.336 | |
| ** p<0.05   ** p<0.01   *** p<0.001* | | | | | | | | |

Table S61: Multi-level model estimates for (log) household consumption of *Cooked meals* with household- and district-level covariates.

|  | **Cooked Meals** | | **Cooked Meals** | | **Cooked Meals** | | **Cooked Meals** | |
| --- | --- | --- | --- | --- | --- | --- | --- | --- |
| *Predictors* | *Estimates* | *CI* | *Estimates* | *CI* | *Estimates* | *CI* | *Estimates* | *CI* |
| Intercept | 2.11 ^***^ | 2.00 – 2.21 | -0.76 ^***^ | -1.06 – -0.46 | -1.06 ^***^ | -1.37 – -0.75 | -1.73 ^***^ | -2.48 – -0.97 |
| Income |  |  | 0.31 ^***^ | 0.28 – 0.34 | 0.37 ^***^ | 0.33 – 0.40 | 0.37 ^***^ | 0.33 – 0.40 |
| Urban |  |  | -0.06 ^**^ | -0.10 – -0.02 | 0.01 | -0.04 – 0.05 | 0.01 | -0.03 – 0.05 |
| Household Size |  |  |  |  | -0.01 | -0.06 – 0.05 | -0.00 | -0.06 – 0.05 |
| Household Structure |  |  |  |  | -0.03 | -0.07 – 0.01 | -0.03 | -0.07 – 0.01 |
| Cooking Facility |  |  |  |  | -0.22 ^***^ | -0.27 – -0.18 | -0.22 ^***^ | -0.27 – -0.18 |
| Infrastructure |  |  |  |  |  |  | 0.09 ^**^ | 0.03 – 0.14 |
| Travel time to Cities |  |  |  |  |  |  | 0.04 | -0.04 – 0.13 |
| % Working Women in Urban Areas |  |  |  |  |  |  | -2.48 ^*^ | -4.76 – -0.20 |
| **Random Effects** | | | | | | | | |
| σ^2^ | 0.84 | | 0.81 | | 0.80 | | 0.80 | |
| (τ_2_)^2^ | 0.11 _District:State_ | | 0.10 _District:State_ | | 0.10 _District:State_ | | 0.10 _District:State_ | |
| (τ_1_)^2^ | 0.08 _State_ | | 0.08 _State_ | | 0.07 _State_ | | 0.04 _State_ | |
| ICC | 0.18 | | 0.18 | | 0.17 | | 0.15 | |
| N | 67 _District_ | | 67 _District_ | | 67 _District_ | | 67 _District_ | |
|  | 35 _State_ | | 35 _State_ | | 35 _State_ | | 35 _State_ | |
| Observations | 10054 | | 10054 | | 10054 | | 10054 | |
| Marginal R^2^ / Conditional R^2^ | 0.000 / 0.180 | | 0.039 / 0.215 | | 0.049 / 0.215 | | 0.057 / 0.199 | |
| ** p<0.05   ** p<0.01   *** p<0.001* | | | | | | | | |

Table S62: Multi-level model estimates for (log) household consumption of *Cooked meals (free)* with household- and district-level covariates.

|  | **Cooked Meals Free** | | **Cooked Meals Free** | | **Cooked Meals Free** | | **Cooked Meals Free** | |
| --- | --- | --- | --- | --- | --- | --- | --- | --- |
| *Predictors* | *Estimates* | *CI* | *Estimates* | *CI* | *Estimates* | *CI* | *Estimates* | *CI* |
| Intercept | 2.85 ^***^ | 2.74 – 2.96 | 2.43 ^***^ | 1.76 – 3.10 | 2.57 ^***^ | 1.82 – 3.32 | 1.04 | -0.12 – 2.19 |
| Income |  |  | 0.04 | -0.03 – 0.12 | 0.06 | -0.03 – 0.15 | 0.06 | -0.03 – 0.15 |
| Urban |  |  | 0.07 | -0.02 – 0.17 | 0.09 | -0.01 – 0.19 | 0.10 | -0.00 – 0.20 |
| Household Size |  |  |  |  | 0.10 | -0.04 – 0.24 | 0.09 | -0.05 – 0.23 |
| Household Structure |  |  |  |  | -0.14 ^**^ | -0.24 – -0.05 | -0.14 ^**^ | -0.23 – -0.05 |
| Cooking Facility |  |  |  |  | -0.09 | -0.20 – 0.03 | -0.07 | -0.19 – 0.04 |
| Infrastructure |  |  |  |  |  |  | 0.16 ^***^ | 0.09 – 0.24 |
| Travel time to Cities |  |  |  |  |  |  | 0.13 ^*^ | 0.01 – 0.25 |
| % Working Women in Urban Areas |  |  |  |  |  |  | -3.75 ^*^ | -6.88 – -0.62 |
| **Random Effects** | | | | | | | | |
| σ^2^ | 0.59 | | 0.59 | | 0.59 | | 0.59 | |
| (τ_2_)^2^ | 0.09 _District:State_ | | 0.09 _District:State_ | | 0.09 _District:State_ | | 0.11 _District:State_ | |
| (τ_1_)^2^ | 0.05 _State_ | | 0.06 _State_ | | 0.06 _State_ | | 0.01 _State_ | |
| ICC | 0.20 | | 0.20 | | 0.21 | | 0.17 | |
| N | 54 _District_ | | 54 _District_ | | 54 _District_ | | 54 _District_ | |
|  | 34 _State_ | | 34 _State_ | | 34 _State_ | | 34 _State_ | |
| Observations | 1351 | | 1351 | | 1351 | | 1351 | |
| Marginal R^2^ / Conditional R^2^ | 0.000 / 0.200 | | 0.003 / 0.202 | | 0.011 / 0.216 | | 0.036 / 0.198 | |
| ** p<0.05   ** p<0.01   *** p<0.001* | | | | | | | | |

Table S63: Multi-level model estimates for (log) household consumption of *Snacks* with household- and district-level covariates.

|  | **Snacks** | | **Snacks** | | **Snacks** | | **Snacks** | |
| --- | --- | --- | --- | --- | --- | --- | --- | --- |
| *Predictors* | *Estimates* | *CI* | *Estimates* | *CI* | *Estimates* | *CI* | *Estimates* | *CI* |
| Intercept | 5.76 ^***^ | 5.67 – 5.85 | 1.46 ^***^ | 1.33 – 1.58 | 1.63 ^***^ | 1.50 – 1.75 | 1.00 ^***^ | 0.53 – 1.47 |
| Income |  |  | 0.48 ^***^ | 0.47 – 0.49 | 0.44 ^***^ | 0.43 – 0.45 | 0.44 ^***^ | 0.43 – 0.45 |
| Urban |  |  | 0.02 ^**^ | 0.00 – 0.03 | 0.01 | -0.00 – 0.02 | 0.01 | -0.00 – 0.02 |
| Household Size |  |  |  |  | 0.09 ^***^ | 0.07 – 0.10 | 0.09 ^***^ | 0.07 – 0.10 |
| Household Structure |  |  |  |  | -0.00 | -0.01 – 0.01 | -0.00 | -0.02 – 0.01 |
| Cooking Facility |  |  |  |  | 0.05 ^***^ | 0.03 – 0.07 | 0.05 ^***^ | 0.03 – 0.07 |
| Infrastructure |  |  |  |  |  |  | 0.05 ^**^ | 0.01 – 0.08 |
| Travel time to Cities |  |  |  |  |  |  | 0.07 ^*^ | 0.01 – 0.13 |
| % Working Women in Urban Areas |  |  |  |  |  |  | -0.11 | -1.78 – 1.55 |
| **Random Effects** | | | | | | | | |
| σ^2^ | 0.47 | | 0.40 | | 0.40 | | 0.40 | |
| (τ_2_)^2^ | 0.07 _District:State_ | | 0.06 _District:State_ | | 0.06 _District:State_ | | 0.06 _District:State_ | |
| (τ_1_)^2^ | 0.06 _State_ | | 0.05 _State_ | | 0.05 _State_ | | 0.05 _State_ | |
| ICC | 0.22 | | 0.22 | | 0.22 | | 0.23 | |
| N | 71 _District_ | | 71 _District_ | | 71 _District_ | | 71 _District_ | |
|  | 35 _State_ | | 35 _State_ | | 35 _State_ | | 35 _State_ | |
| Observations | 46784 | | 46784 | | 46784 | | 46784 | |
| Marginal R^2^ / Conditional R^2^ | 0.000 / 0.218 | | 0.150 / 0.340 | | 0.151 / 0.341 | | 0.157 / 0.350 | |
| ** p<0.05   ** p<0.01   *** p<0.001* | | | | | | | | |

Table S64: Multi-level model estimates for (log) household consumption of *Chips* with household- and district-level covariates.

|  | **Chips** | | **Chips** | | **Chips** | | **Chips** | |
| --- | --- | --- | --- | --- | --- | --- | --- | --- |
| *Predictors* | *Estimates* | *CI* | *Estimates* | *CI* | *Estimates* | *CI* | *Estimates* | *CI* |
| Intercept | 4.83 ^***^ | 4.70 – 4.96 | 1.28 ^***^ | 1.04 – 1.52 | 1.49 ^***^ | 1.24 – 1.75 | 1.28 ^***^ | 0.57 – 2.00 |
| Income |  |  | 0.38 ^***^ | 0.36 – 0.41 | 0.35 ^***^ | 0.33 – 0.38 | 0.35 ^***^ | 0.32 – 0.38 |
| Urban |  |  | 0.05 ^***^ | 0.02 – 0.08 | 0.03 | -0.00 – 0.06 | 0.03 | -0.00 – 0.06 |
| Household Size |  |  |  |  | 0.07 ^***^ | 0.03 – 0.11 | 0.07 ^***^ | 0.03 – 0.11 |
| Household Structure |  |  |  |  | -0.02 | -0.06 – 0.01 | -0.02 | -0.06 – 0.01 |
| Cooking Facility |  |  |  |  | 0.09 ^***^ | 0.06 – 0.12 | 0.09 ^***^ | 0.06 – 0.12 |
| Infrastructure |  |  |  |  |  |  | 0.01 | -0.05 – 0.06 |
| Travel time to Cities |  |  |  |  |  |  | 0.03 | -0.06 – 0.11 |
| % Working Women in Urban Areas |  |  |  |  |  |  | 1.96 | -0.30 – 4.22 |
| **Random Effects** | | | | | | | | |
| σ^2^ | 0.57 | | 0.52 | | 0.52 | | 0.52 | |
| (τ_2_)^2^ | 0.12 _District:State_ | | 0.10 _District:State_ | | 0.10 _District:State_ | | 0.10 _District:State_ | |
| (τ_1_)^2^ | 0.14 _State_ | | 0.11 _State_ | | 0.11 _State_ | | 0.10 _State_ | |
| ICC | 0.31 | | 0.29 | | 0.29 | | 0.28 | |
| N | 70 _District_ | | 70 _District_ | | 70 _District_ | | 70 _District_ | |
|  | 35 _State_ | | 35 _State_ | | 35 _State_ | | 35 _State_ | |
| Observations | 12712 | | 12712 | | 12712 | | 12712 | |
| Marginal R^2^ / Conditional R^2^ | 0.000 / 0.310 | | 0.074 / 0.340 | | 0.076 / 0.343 | | 0.083 / 0.340 | |
| ** p<0.05   ** p<0.01   *** p<0.001* | | | | | | | | |

Table S65: Multi-level model estimates for (log) household consumption of *Pickles* with household- and district-level covariates.

|  | **Pickles** | | **Pickles** | | **Pickles** | | **Pickles** | |
| --- | --- | --- | --- | --- | --- | --- | --- | --- |
| *Predictors* | *Estimates* | *CI* | *Estimates* | *CI* | *Estimates* | *CI* | *Estimates* | *CI* |
| Intercept | 5.21 ^***^ | 5.08 – 5.34 | 2.33 ^***^ | 2.11 – 2.54 | 2.46 ^***^ | 2.23 – 2.68 | 1.69 ^***^ | 1.04 – 2.34 |
| Income |  |  | 0.32 ^***^ | 0.30 – 0.34 | 0.28 ^***^ | 0.25 – 0.30 | 0.28 ^***^ | 0.25 – 0.30 |
| Urban |  |  | -0.08 ^***^ | -0.11 – -0.05 | -0.05 ^***^ | -0.08 – -0.02 | -0.05 ^***^ | -0.08 – -0.02 |
| Household Size |  |  |  |  | 0.19 ^***^ | 0.15 – 0.23 | 0.19 ^***^ | 0.15 – 0.23 |
| Household Structure |  |  |  |  | -0.01 | -0.03 – 0.02 | -0.01 | -0.03 – 0.02 |
| Cooking Facility |  |  |  |  | -0.02 | -0.05 – 0.01 | -0.02 | -0.05 – 0.01 |
| Infrastructure |  |  |  |  |  |  | 0.04 | -0.01 – 0.09 |
| Travel time to Cities |  |  |  |  |  |  | 0.10 ^*^ | 0.02 – 0.18 |
| % Working Women in Urban Areas |  |  |  |  |  |  | 0.28 | -1.85 – 2.41 |
| **Random Effects** | | | | | | | | |
| σ^2^ | 0.42 | | 0.38 | | 0.38 | | 0.38 | |
| (τ_2_)^2^ | 0.10 _District:State_ | | 0.09 _District:State_ | | 0.09 _District:State_ | | 0.09 _District:State_ | |
| (τ_1_)^2^ | 0.14 _State_ | | 0.11 _State_ | | 0.11 _State_ | | 0.10 _State_ | |
| ICC | 0.36 | | 0.34 | | 0.35 | | 0.33 | |
| N | 65 _District_ | | 65 _District_ | | 65 _District_ | | 65 _District_ | |
|  | 35 _State_ | | 35 _State_ | | 35 _State_ | | 35 _State_ | |
| Observations | 12149 | | 12149 | | 12149 | | 12149 | |
| Marginal R^2^ / Conditional R^2^ | 0.000 / 0.365 | | 0.064 / 0.387 | | 0.071 / 0.394 | | 0.078 / 0.386 | |
| ** p<0.05   ** p<0.01   *** p<0.001* | | | | | | | | |

Table S66: Multi-level model estimates for (log) household consumption of *Sauce* with household- and district-level covariates.

|  | **Sauce** | | **Sauce** | | **Sauce** | | **Sauce** | |
| --- | --- | --- | --- | --- | --- | --- | --- | --- |
| *Predictors* | *Estimates* | *CI* | *Estimates* | *CI* | *Estimates* | *CI* | *Estimates* | *CI* |
| Intercept | 5.34 ^***^ | 5.23 – 5.46 | 1.56 ^***^ | 1.12 – 2.00 | 1.64 ^***^ | 1.19 – 2.09 | 0.75 | -0.18 – 1.68 |
| Income |  |  | 0.39 ^***^ | 0.35 – 0.44 | 0.37 ^***^ | 0.32 – 0.42 | 0.36 ^***^ | 0.32 – 0.41 |
| Urban |  |  | 0.07 ^*^ | 0.00 – 0.13 | 0.06 | -0.01 – 0.13 | 0.06 | -0.01 – 0.13 |
| Household Size |  |  |  |  | 0.11 ^**^ | 0.03 – 0.19 | 0.11 ^**^ | 0.03 – 0.19 |
| Household Structure |  |  |  |  | -0.02 | -0.08 – 0.04 | -0.02 | -0.08 – 0.04 |
| Cooking Facility |  |  |  |  | 0.07 | -0.01 – 0.16 | 0.07 | -0.01 – 0.15 |
| Infrastructure |  |  |  |  |  |  | 0.05 | -0.01 – 0.12 |
| Travel time to Cities |  |  |  |  |  |  | 0.11 ^*^ | 0.01 – 0.22 |
| % Working Women in Urban Areas |  |  |  |  |  |  | 1.62 | -1.10 – 4.34 |
| **Random Effects** | | | | | | | | |
| σ^2^ | 0.60 | | 0.56 | | 0.56 | | 0.56 | |
| (τ_2_)^2^ | 0.11 _District:State_ | | 0.09 _District:State_ | | 0.10 _District:State_ | | 0.09 _District:State_ | |
| (τ_1_)^2^ | 0.08 _State_ | | 0.06 _State_ | | 0.06 _State_ | | 0.07 _State_ | |
| ICC | 0.24 | | 0.22 | | 0.22 | | 0.22 | |
| N | 62 _District_ | | 62 _District_ | | 62 _District_ | | 62 _District_ | |
|  | 33 _State_ | | 33 _State_ | | 33 _State_ | | 33 _State_ | |
| Observations | 3568 | | 3568 | | 3568 | | 3568 | |
| Marginal R^2^ / Conditional R^2^ | 0.000 / 0.243 | | 0.081 / 0.283 | | 0.082 / 0.283 | | 0.091 / 0.294 | |
| ** p<0.05   ** p<0.01   *** p<0.001* | | | | | | | | |


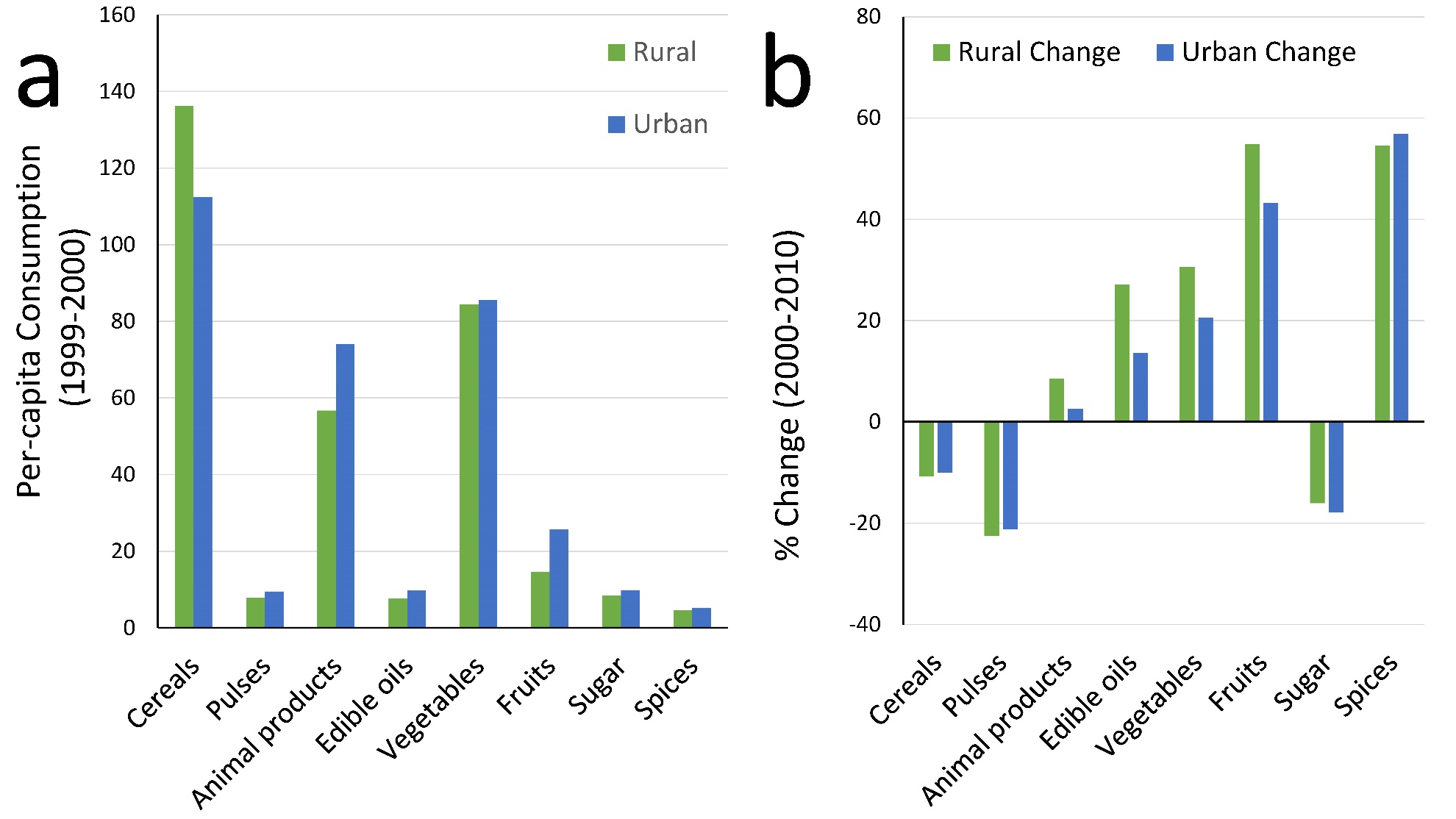


Figure S1: a) Quantities of food consumed (kg) in urban areas are generally higher than quantities consumed in rural areas for all commodities but cereals. b) declining per capita consumption of cereals, pulses, and sugar in urban as well as rural areas and increasing per capita consumption of other food commodities (such as, animal products, oils, and fruits and vegetables) in urban as well as rural areas, and more rapid changes in per-capita consumption in rural areas than in urban areas for most food groups except sugar and spices. Data Source: National Sample Survey Office (NSSO).


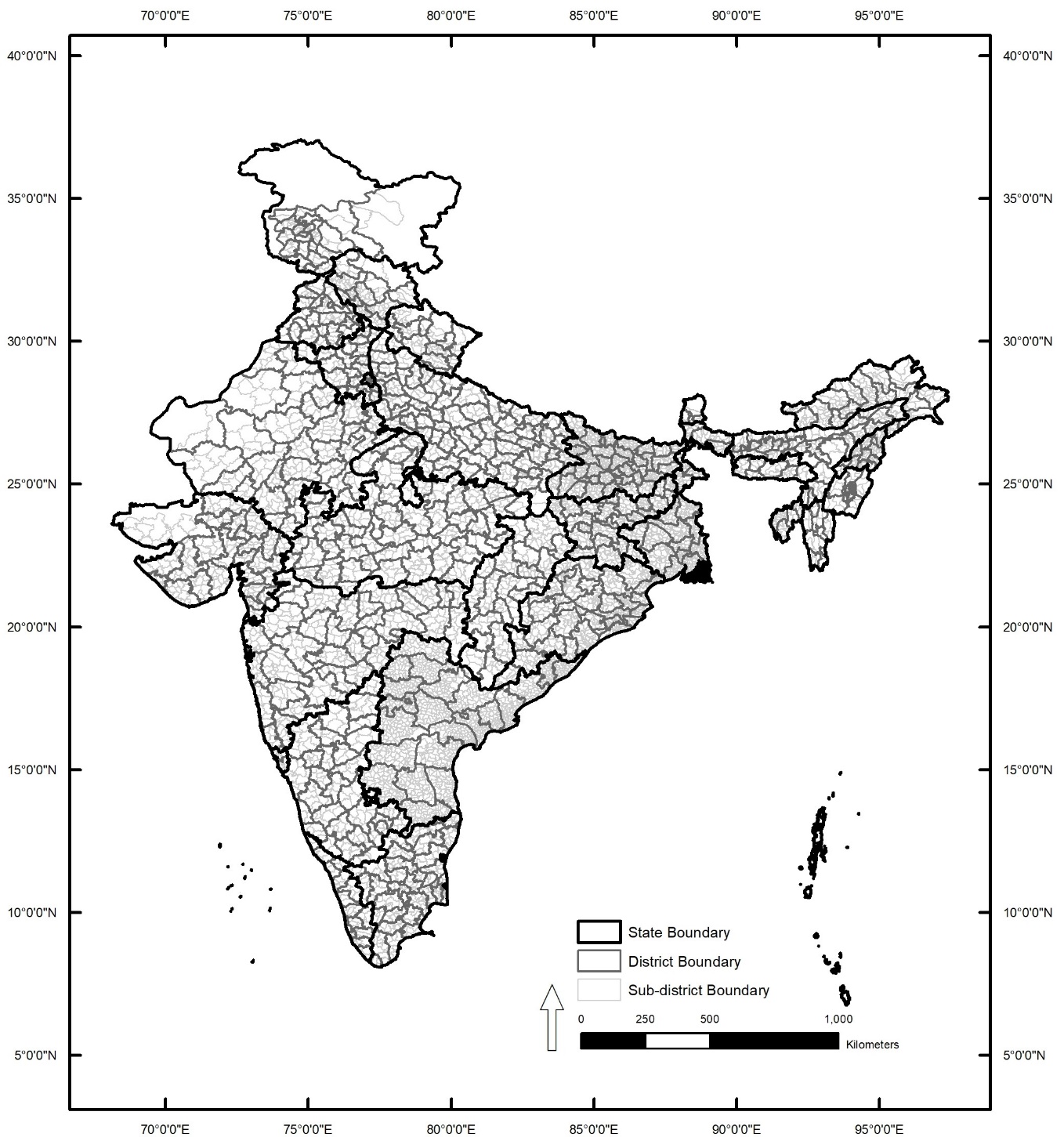


Figure S2: State and union territory (UT), district, and sub-district boundaries in India as per the 2011 census.


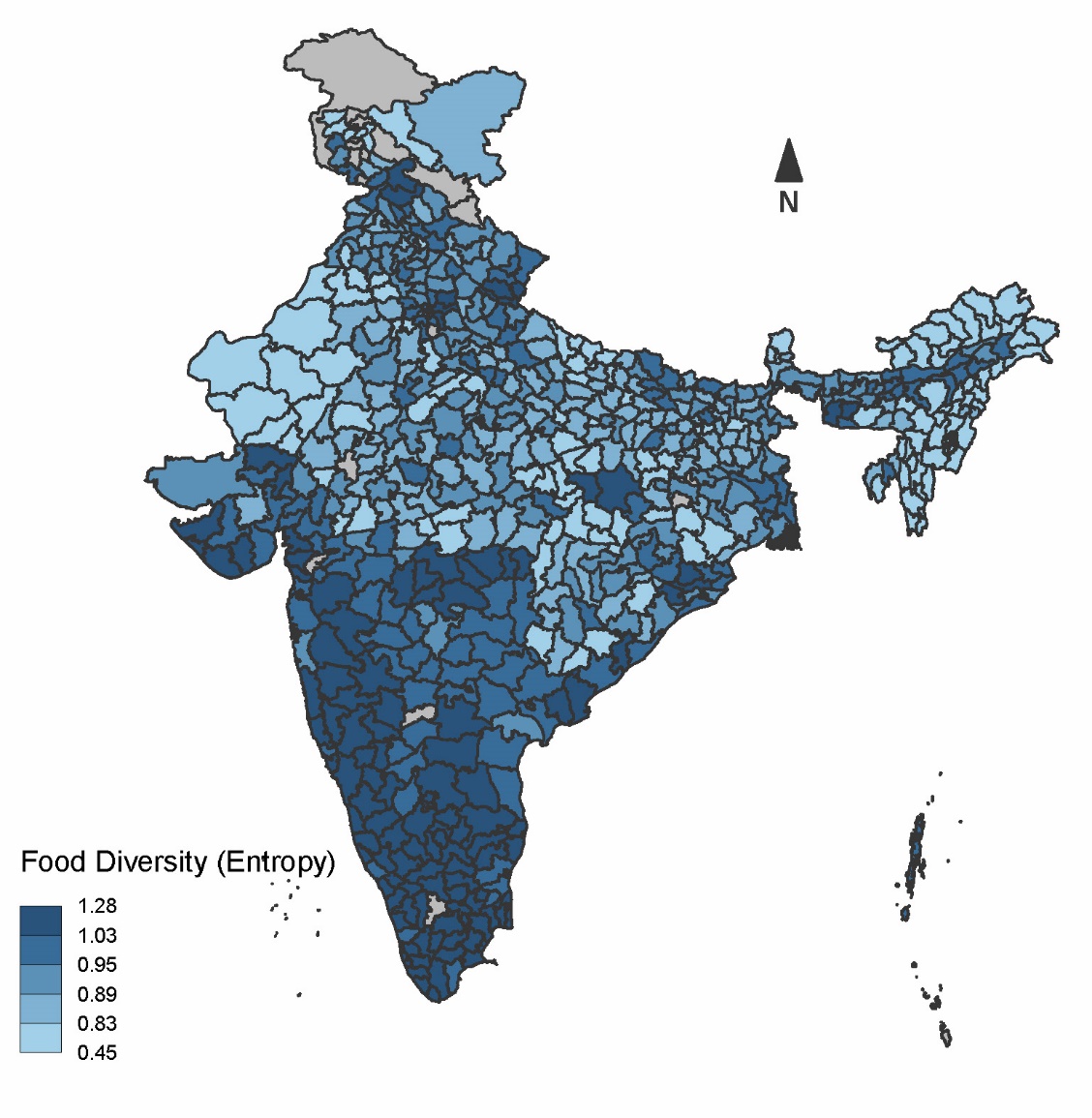


Figure S3: District-level bootstrap estimates of average food consumption diversity across India. Districts with no data are shown with gray color.


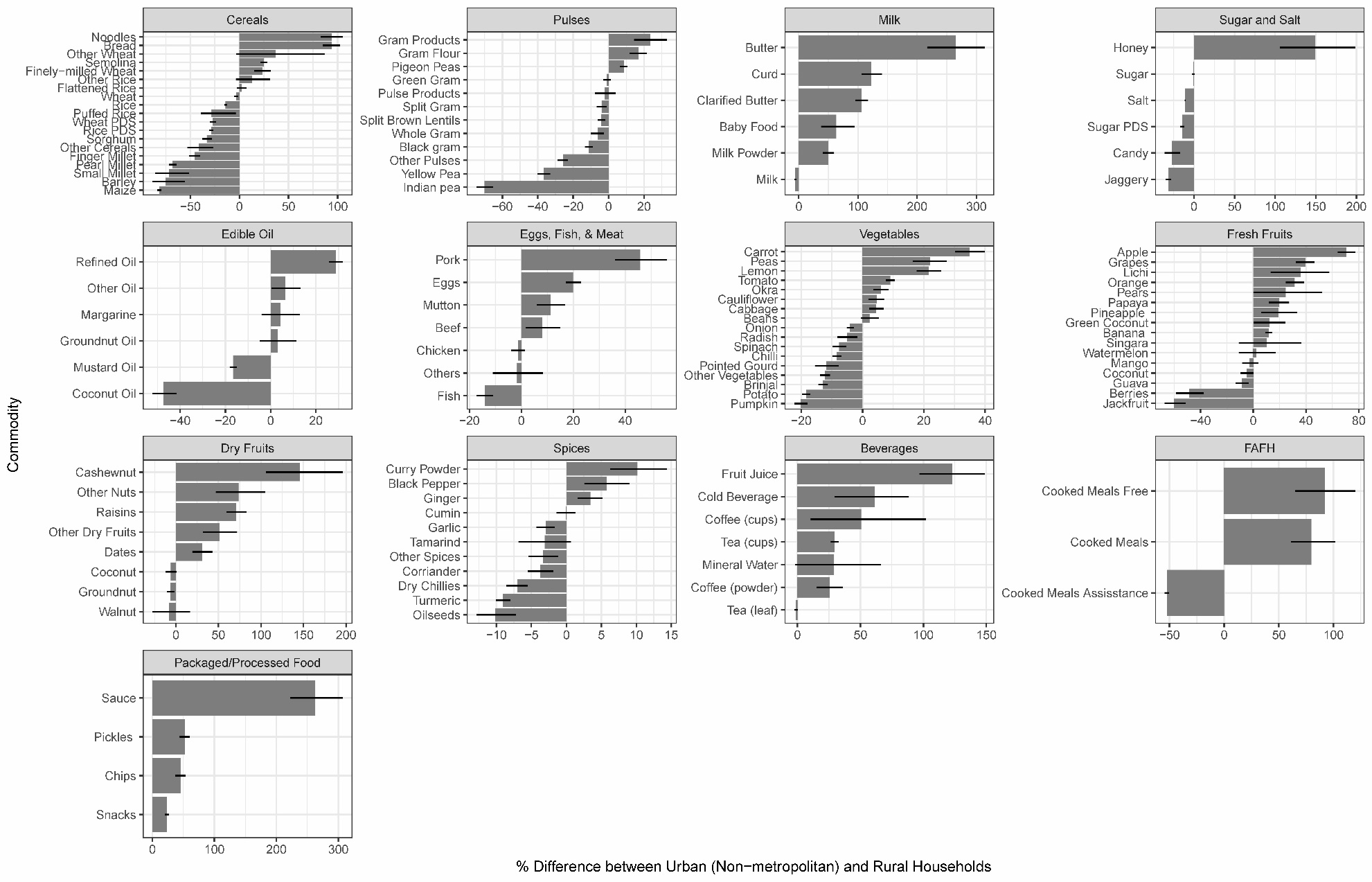


Figure S4: Bootstrap difference in average household consumption between urban (non-metropolitan) and rural areas by commodity (n=124) and commodity types. The x-axes show average bootstrap estimates of percentage difference by which average consumption by households in urban areas differs over households in rural areas. The black line at the end of each bar shows the 95% confidence interval.


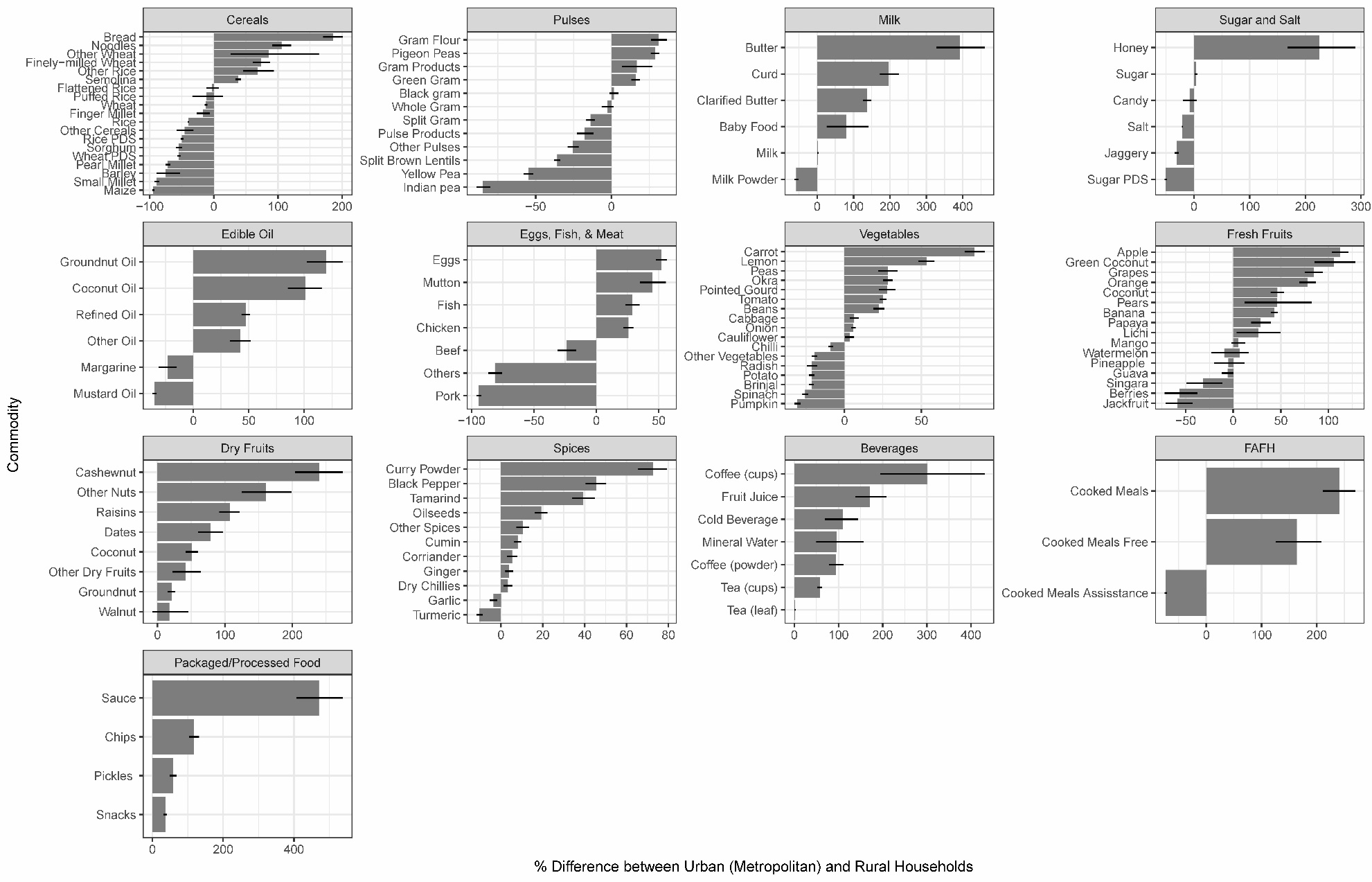


Figure S5: Bootstrap difference in average household consumption between urban (metropolitan) and rural areas by commodity (n=124) and commodity types. The x-axes show average bootstrap estimates of percentage difference by which average consumption by households in urban areas differs over households in rural areas. The black line at the end of each bar shows the 95% confidence interval.


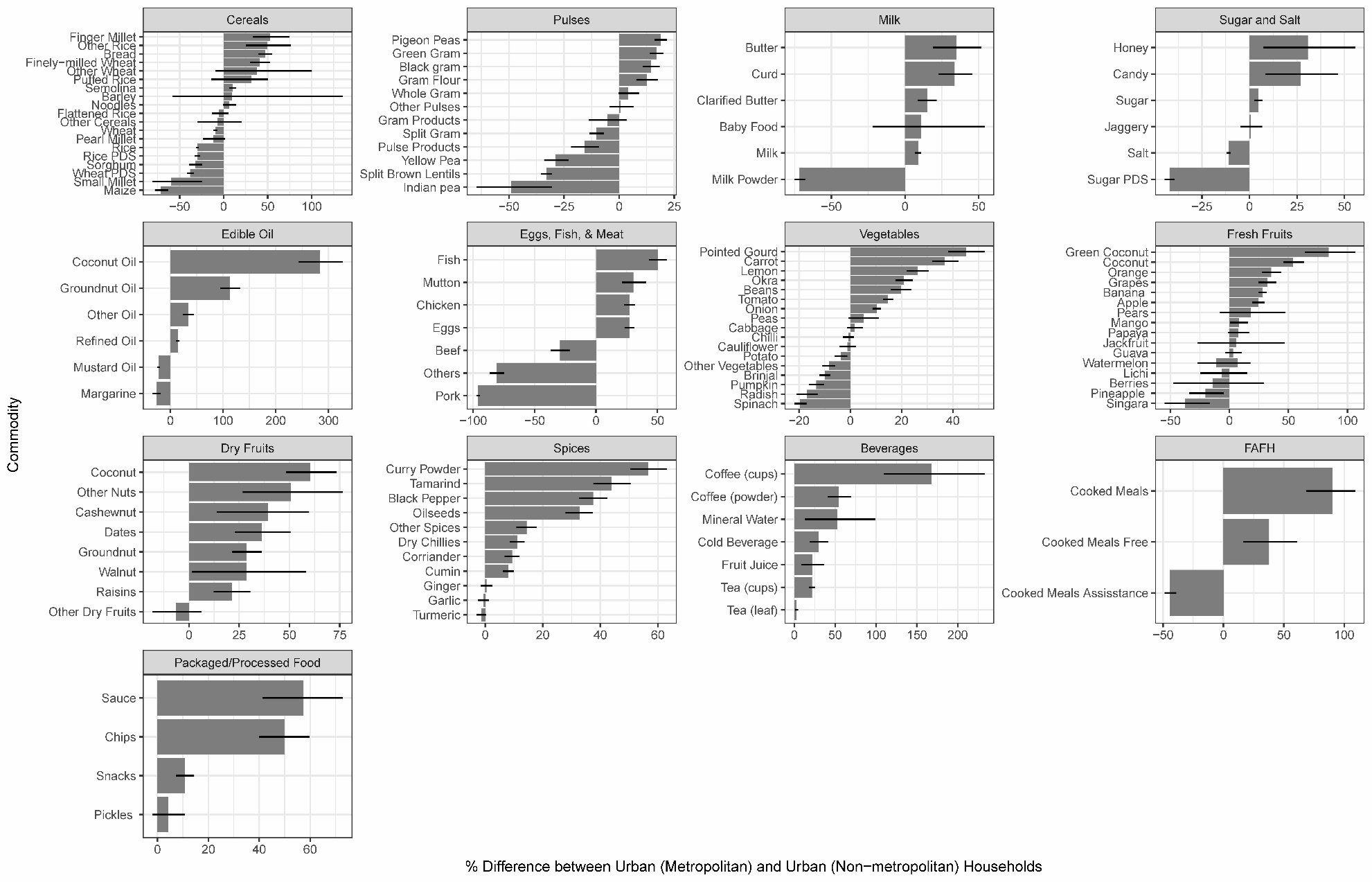


Figure S6: Bootstrap difference in average household consumption between urban metropolitan and urban non-metropolitan areas by commodity (n=124) and commodity types. The x-axes show average bootstrap estimates of percentage difference by which average consumption by households in urban metropolitan areas differs over households in urban non-metropolitan areas. The black line at the end of each bar shows the 95% confidence interval.


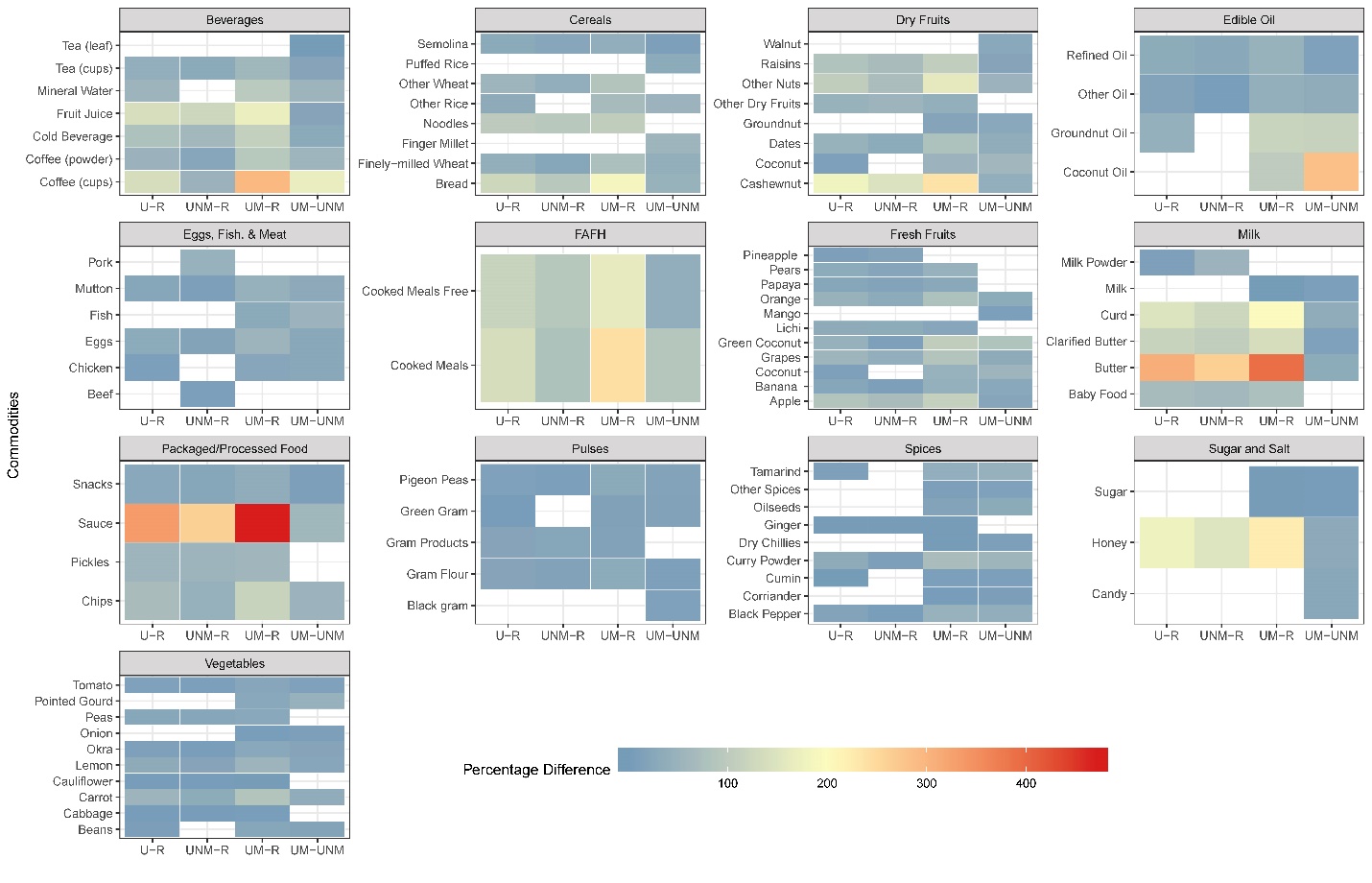


Figure S7: Positive influence of urbanization observed on quantities of food consumed by households for food commodities organized by 13 groups. Colors show the average bootstrap estimates of percentage difference with which average consumption by households in urban areas (U: Urban; UNM: Urban Non-metropolitan; UM: Urban Metropolitan) exceeds average consumption by households in rural areas (R: Rural). Figure also shows the percentage difference with which average consumption by households in urban metropolitan areas (UM) exceeds consumption by households in urban metropolitan (UM) areas

# **S1 Data sources and variables**

The present study examined urbanization and food consumption in India, using multi-scale datasets from different sources. Here, consumer expenditure survey with other available datasets captured different aspects of urbanization and food consumption. Table S1.1 provides a description and sources of these datasets.

| **S. No.** | **Dataset** | **Scale** | **Time** | **Source** | **Relevant Variables** | **URL** |
| --- | --- | --- | --- | --- | --- | --- |
| 1. | Consumer Expenditure Survey (Round 68^th^) | Household-level | 2011-2012 | National Sample Survey Office | Quantities of food consumed, place of residence (urban/rural), geographical information (state and district), household income, household size, household member’s age, and cooking facility. | <http://microdata.gov.in/nada43/index.php/home> |
| 2. | Census, 2011 | State and district level | 2011 | Census of India | Urban and total population, female population working in urban areas, and cities with greater than 1 million population size. | <https://censusindia.gov.in/> |
| 3. | Global Human Settlements Layer (GHSL) | Pixel level (~38m spatial resolution) | 2014 | EC-JRC | Built-up area | <http://cidportal.jrc.ec.europa.eu/ftp/jrc-opendata/GHSL/GHS_BUILT_LDSMT_GLOBE_R2015B/> |
| 4. | DMSP/OLS nighttime lights | Pixel level (~1km spatial resolution) | 2011 | Zhang et al. ^1^ | Nighttime lights intensity | <https://yale.box.com/s/xwai7cjwpfuenqze8aa8jsxiizinwrpz> |
| 5. | Global Accessibility | Pixel level (~1km spatial resolution) | 2000 | Nelson ^2^ | Travel time to cities | <http://forobs.jrc.ec.europa.eu/products/gam/download.php> |

# **S2 Methodological description**

## **S2.1 Bootstrap estimation**

The present study used nonparametric bootstrap estimation to examine differences in average food consumption and consumption diversity between urban (U) and rural (R) areas, and between urban metropolitan (UM), urban non-metropolitan (UNM), and rural (R) areas in India^3^. Bootstrapping is essential because quantities consumed across households often do not follow a normal distribution. This estimation approach involves random sampling of the data with replacement with sample size equal to the number of observations. Accordingly, the analysis involved estimating bootstrapped mean estimates U, R, UM, and UNM groups. Then, it estimated the percentage difference between the mean quantities for each pair of groups. We repeated this procedure 1,000 times to generate a distribution of difference (%) in average consumption between two groups. From this distribution, we estimated the mean percentage difference and 95% confidence intervals. Using this approach, we examined differences for 124 food commodities and the food diversity index.

## **S2.2 Quantities of food consumed**

Quantity of food consumed is defined as food bought by a household that may be eaten (consumed) or wasted. We grouped all the food commodities from the consumer expenditure survey into 13 categories: cereals, pulses, milk, sugar and salt, edible oil, eggs fish and meat, vegetables, fresh fruits, dry fruits, spices, beverages, food prepared away from home (FAFH), and packaged/processed food (Table S2.2.1). The analysis excluded cereal substitutes, given a lack of detail in the survey. We examined differences in average quantities of food consumed between U and R areas, and between UM, UNM, and R areas across 124 commodities using bootstrapping. Subsequently, the analysis identified the commodities for which average quantities consumed increased along with the urban progression (UM > UNM > R or U > R). In addition to the urban-rural comparisons, we estimated commodity-wise Pearson’s correlation coefficients (*r*) between (log) average quantities consumed and urbanization (as the share of urban population to total population) at the state and district levels. Similarly, we also examined commodity-wise correlations between (log) average quantities and (log) income at the household level. Finally, we estimated OLS regression models using data at the household level and accounting for household income (represented by total expenditure) and state-level effects (such as price effects) to identify commodities for which a unique urban effect exists (UM > UNM > R or U > R). Here we estimated multilevel models to assess the robustness of the results.

Table S2.2.1: List of 124 food commodities classified into 13 food groups.

| S.No. | Name in the Survey | Name (modified) | Food Group |
| --- | --- | --- | --- |
| 1 | Rice-PDS(Kg) | Rice PDS | Cereals |
| 2 | Rice-other sources(Kg) | Rice | Cereals |
| 3 | Chira(Kg) | Flattened Rice | Cereals |
| 4 | Khoi, lava(Kg) | Puffed Rice | Cereals |
| 5 | Muri(Kg) | Puffed Rice | Cereals |
| 6 | Other rice products(Kg) | Other Rice | Cereals |
| 7 | Wheat/atta - P.D.S (Kg) | Wheat PDS | Cereals |
| 8 | Wheat/atta - other sources | Wheat | Cereals |
| 9 | Maida(Kg) | Finely-milled Wheat | Cereals |
| 10 | suji, rawa(Kg) | Semolina | Cereals |
| 11 | sewai, noodles(Kg) | Noodles | Cereals |
| 12 | bread(bakery)(Kg) | Bread | Cereals |
| 13 | other wheat products(Kg) | Other Wheat | Cereals |
| 14 | jowar & products(Kg) | Sorghum | Cereals |
| 15 | bajra & products(Kg) | Pearl Millet | Cereals |
| 16 | maize & products(Kg) | Maize | Cereals |
| 17 | barley & products(Kg) | Barley | Cereals |
| 18 | small millet & products(Kg) | Small Millet | Cereals |
| 19 | ragi & products(Kg) | Finger Millet | Cereals |
| 20 | other cereals(Kg) | Other Cereals | Cereals |
| 21 | arhar (tur)(Kg) | Pigeon Peas | Pulses |
| 22 | gram : split Kg | Split Gram | Pulses |
| 23 | gram : whole Kg | Whole Gram | Pulses |
| 24 | moong(Kg) | Green Gram | Pulses |
| 25 | masur(Kg) | Split Brown Lentils | Pulses |
| 26 | urd(Kg) | Black gram | Pulses |
| 27 | peas(Kg).x | Yellow Pea | Pulses |
| 28 | khesari(Kg) | Indian pea | Pulses |
| 29 | other pulses(Kg) | Other Pulses | Pulses |
| 30 | gram products(kg) | Gram Products | Pulses |
| 31 | besan(Kg) | Gram Flour | Pulses |
| 32 | other pulse products(Kg) | Pulse Products | Pulses |
| 33 | milk : liquid(litre) | Milk | Milk |
| 34 | baby food(Kg) | Baby Food | Milk |
| 35 | milk : cond./powder(Kg) | Milk Powder | Milk |
| 36 | curd(Kg) | Curd | Milk |
| 37 | ghee(Kg) | Clarified Butter | Milk |
| 38 | butter(Kg) | Butter | Milk |
| 39 | salt | Salt | Sugar and Salt |
| 40 | sugar - PDS | Sugar PDS | Sugar and Salt |
| 41 | sugar - other sources | Sugar | Sugar and Salt |
| 42 | gur | Jaggery | Sugar and Salt |
| 43 | candy, misri | Candy | Sugar and Salt |
| 44 | honey | Honey | Sugar and Salt |
| 45 | vanaspati, margarine(Kg) | Margarine | Edible Oil |
| 46 | mustard oil(Kg) | Mustard Oil | Edible Oil |
| 47 | groundnut - oil (Kg) | Groundnut Oil | Edible Oil |
| 48 | coconut oil(Kg) | Coconut Oil | Edible Oil |
| 49 | refined oil [sunflower, soyabean, saffola, etc] | Refined Oil | Edible Oil |
| 50 | edible oil : others | Other Oil | Edible Oil |
| 51 | eggs(no) | Eggs | Eggs, Fish, & Meat |
| 52 | fish, prawn(Kg) | Fish | Eggs, Fish, & Meat |
| 53 | goat meat /mutton(Kg) | Mutton | Eggs, Fish, & Meat |
| 54 | beef / buffalo meat(Kg) | Beef | Eggs, Fish, & Meat |
| 55 | pork(Kg) | Pork | Eggs, Fish, & Meat |
| 56 | chicken(Kg) | Chicken | Eggs, Fish, & Meat |
| 57 | others ( birds, crab, oyster, tortoise etc | Others | Eggs, Fish, & Meat |
| 58 | potato(Kg) | Potato | Vegetables |
| 59 | onion(Kg) | Onion | Vegetables |
| 60 | tomato(Kg) | Tomato | Vegetables |
| 61 | brinjal(Kg) | Brinjal | Vegetables |
| 62 | radish(Kg) | Radish | Vegetables |
| 63 | carrot(Kg) | Carrot | Vegetables |
| 64 | palak/other leafy vegetables(Kg) | Spinach | Vegetables |
| 65 | green chillies(Kg) | Chilli | Vegetables |
| 66 | lady's finger(Kg) | Okra | Vegetables |
| 67 | parwal/patal, kundru(Kg) | Pointed Gourd | Vegetables |
| 68 | cauliflower(Kg) | Cauliflower | Vegetables |
| 69 | cabbage(Kg) | Cabbage | Vegetables |
| 70 | gourd, pumpkin(Kg) | Pumpkin | Vegetables |
| 71 | peas(Kg).y | Peas | Vegetables |
| 72 | beans, barbati(Kg) | Beans | Vegetables |
| 73 | lemon(no.) | Lemon | Vegetables |
| 74 | other vegetables(Kg) | Other Vegetables | Vegetables |
| 75 | banana (no.) | Banana | Fresh Fruits |
| 76 | jackfruit(kgs) | Jackfruit | Fresh Fruits |
| 77 | watermelon(kg) | Watermelon | Fresh Fruits |
| 78 | pineapple (no.) | Pineapple | Fresh Fruits |
| 79 | coconut(no.) | Coconut | Fresh Fruits |
| 80 | green coconut(no.) | Green Coconut | Fresh Fruits |
| 81 | guava(Kg) | Guava | Fresh Fruits |
| 82 | singara(Kg) | Singara | Fresh Fruits |
| 83 | orange, mausambi (no.) | Orange | Fresh Fruits |
| 84 | papaya(Kg) | Papaya | Fresh Fruits |
| 85 | mango(kg) | Mango | Fresh Fruits |
| 86 | kharbooza(Kg) | Watermelon | Fresh Fruits |
| 87 | pears/nashpati(Kg) | Pears | Fresh Fruits |
| 88 | berries(Kg) | Berries | Fresh Fruits |
| 89 | leechi(Kg) | Lichi | Fresh Fruits |
| 90 | apple(Kg) | Apple | Fresh Fruits |
| 91 | grapes(Kg) | Grapes | Fresh Fruits |
| 92 | coconut:copra(kg) | Coconut | Dry Fruits |
| 93 | groundnut(Kg) | Groundnut | Dry Fruits |
| 94 | dates(Kg) | Dates | Dry Fruits |
| 95 | cashewnut(Kg) | Cashewnut | Dry Fruits |
| 96 | walnut(Kg) | Walnut | Dry Fruits |
| 97 | other nuts(Kg) | Other Nuts | Dry Fruits |
| 98 | raisin, (kishmish, monacca etc)(Kg) | Raisins | Dry Fruits |
| 99 | other dry fruits (Kg) | Other Dry Fruits | Dry Fruits |
| 100 | ginger (gm) | Ginger | Spices |
| 101 | garlic (gm) | Garlic | Spices |
| 102 | geera(gm) | Cumin | Spices |
| 103 | dhania(gm) | Coriander | Spices |
| 104 | turmeric (gm) | Turmeric | Spices |
| 105 | black pepper (gm) | Black Pepper | Spices |
| 106 | dry chillies (gm) | Dry Chillies | Spices |
| 107 | tamarind (gm) | Tamarind | Spices |
| 108 | curry powder (gm) | Curry Powder | Spices |
| 109 | oilseeds (gm) | Oilseeds | Spices |
| 110 | other spices (gm) | Other Spices | Spices |
| 111 | tea : cups(no) | Tea (cups) | Beverages |
| 112 | tea : leaf (gm) | Tea (leaf) | Beverages |
| 113 | coffee : cups(no) | Coffee (cups) | Beverages |
| 114 | coffee : powder (gm) | Coffee (powder) | Beverages |
| 115 | mineral water(litre) | Mineral Water | Beverages |
| 116 | cold beverage : bottled/canned (litre) | Cold Beverage | Beverages |
| 117 | fruit juice and shake (litre) | Fruit Juice | Beverages |
| 118 | cooked meals purchased (no.) | Cooked Meals | FAFH |
| 119 | cooked meals received free in workplace(no.) | Cooked Meals Free | FAFH |
| 120 | cooked meals received as assistance (no.) | Cooked Meals Assistance | FAFH |
| 121 | papad, bhujia, namkeen,mixture, chanachur | Snacks | Packaged/Processed Food |
| 122 | chips(gm) | Chips | Packaged/Processed Food |
| 123 | pickles (gm) | Pickles | Packaged/Processed Food |
| 124 | sauce,jam,jelly(gm) | Sauce | Packaged/Processed Food |

## **S2.3 Diversity of food consumed**

Recent research has quantified food and dietary diversity using Shannon’s Entropy Index (SHI) ^4–6^. SHI reflects two aspects of diversity: the number of *k* distinct groups (richness) and the evenness of the distribution of entities across these groups (evenness). The index ranges from ≈0 to *ln(k)*. To quantify household-level food diversity, we transformed the food consumption data to specify which of the food commodities a given household consumes out of all the food commodities (n=124). We then calculated the food diversity index based on SHI for each household. Here we demonstrate the index calculated for six hypothetical households and considering four food groups: cereals, fruits, vegetables, meat (Table S2.3.1). In this example, we assume a total of 24 commodities: cereals (6), fruits (6), vegetables (6), and meat (6). The example shows a positive correlation between food diversity and the number of food commodities consumed. Moreover, unequal distribution leads to lower food diversity than equal distribution. A comparison of household #2 and household #3, each consuming a total of four commodities, exemplifies the impact of evenness on the food diversity index.

Table S2.3.1: Food diversity index for three hypothetical households consuming food items belonging to four food groups.

| Household No. | Cereals | Fruits | Vegetables | Meat | Food Diversity Index |
| --- | --- | --- | --- | --- | --- |
| 1 | 1 | 0 | 0 | 0 | ~0.13 |
| 2 | 1 | 1 | 1 | 1 | ~0.53 |
| 3 | 4 | 0 | 0 | 0 | ~0.30 |
| 4 | 4 | 1 | 1 | 1 | ~0.70 |
| 5 | 5 | 3 | 3 | 2 | ~1.05 |
| 6 | 6 | 6 | 6 | 6 | ~1.39 |

We used a bootstrapping approach to examine differences in food diversity index across U, UM, UNM, and R areas. To further examine the association between food consumption diversity and urbanization, we estimated Pearson’s correlation coefficients between average bootstrapped food diversity index and urbanization at the district and state levels. Additionally, we analyzed the correlations between (log) income and food diversity index at the household level. Finally, the analysis examined the presence of a unique urban effect using OLS regression—such that diversity increases from R to UM areas—after controlling for income and state-level differences.

## **S2.4 Multi-dimensional urbanization influences on food consumption**

While urbanization influences can vary across food commodities, existing literature suggests that urbanization positively influences food diversity^7–10^. We first examined whether urbanization as a demographic share and living in urban areas have a positive influence on food diversity. Next, we examined whether aspects of urbanization, including market access, infrastructure, the proportion of working women in urban areas, and norms and institutions better explain household food diversity in India compared to the urban population share.

Given the multi-scalar influences of urbanization and stratified sampling design of the survey dataset, the assumption of uncorrelated observations in OLS estimation can be problematic. Specifically, the food diversity of households within a region can be correlated and associated with the region’s influence on food diversity. Correlations between households violate the assumption of independent errors. Consequently, we investigated urbanization influences on food diversity in India using multilevel modeling^11^. We used null models, also known as unconditional means models, to identify the baseline multilevel model. Null models exclude independent variables and help in understanding the spatial structuring of the outcome variable. We estimated three null models with varying spatial structuring: (1) with district-level random intercepts using Eq. (1), (2) with state-level random intercepts using Eq. (2), and (3) with district-level random intercepts that are nested within states also with random intercepts, as expressed by Eq. (3).

$E_{ij}=\gamma_{00}+\mu_{0j}+\varepsilon_{ij}$ (1)

Where, $E_{ij}$ is the food diversity index at the household level (*i*), $\gamma_{00}$ is the fixed intercept term that remains constant across districts (*j*), $\mu_{0j}$is the district-specific effect on the intercept that varies randomly with a zero mean and $\tau^{2}$ variance, and $\varepsilon_{ij}$is the normally-distributed error term with zero mean and $\sigma^{2}$ variance.

$E_{ik}=\gamma_{00}+\mu_{0k}+\varepsilon_{ik}$ (2)

Where, $\gamma_{00}$ is the fixed intercept term that remains constant across states (*k*), $\mu_{0k}$is the state-specific effect on the intercept that varies randomly with a zero mean and $\tau^{2}$ variance, and $\varepsilon_{ij}$is the normally-distributed error term with zero mean and $\sigma^{2}$ variance.

$E_{ijk}=\delta_{000}+\mu_{00k}+\gamma_{0jk}+\varepsilon_{ijk}$ (3)

Where, $\delta_{000}$ is the fixed intercept term, $\mu_{00k}$is the state-specific effect on the intercept that varies randomly with a zero mean and ${\tau_{1}}^{2}$ variance, $\gamma_{0jk}$is the district-specific effect on the intercept that varies randomly with a zero mean and ${\tau_{2}}^{2}$ variance, and $\varepsilon_{ijk}$is the normally-distributed error term with zero mean and $\sigma^{2}$ variance.

We then estimated interclass correlation coefficients (ICC)—the proportion of variation in the food diversity index due to between districts and states variations relative to the total variation using Eq. (4).

$ICC= \frac{\tau^{2}}{\tau^{2}+ \sigma^{2}}$ (4)

Where $\tau^{2}$is the variance between regions and $\sigma^{2}$ is the variance within regions. Similarly, we also estimated ICC for the three-level model using Eq. (5).

$ICC= \frac{{\tau_{1}}^{2}+{\tau_{2}}^{2}}{{\tau_{1}}^{2}+{\tau_{2}}^{2}+ \sigma^{2}}$ (5)

Next, we added the household-level and district-level predictors to examine urbanization influences. Here we used variables on household size, structure (standard deviation of all household members’ age), income (total expenditure), cooking facility, and place of residence (UM, UNM, or R). Previous research has highlighted the influence of these factors on aspects of food consumption diversity^10,12,13^. For example, increases in income can drive changes in food consumption diversity through multi-scalar and multi-dimensional influences on food supply and demand^14^. Initial increases in personal income can relax budgetary constraints and yield greater access to food leading to dietary diversification^15^. At the same time, economic development (in low and middle-income countries) and changes in demand can drive technological improvements to increase agricultural productivity influencing food supply^16,17^. Similarly, decreases in household size can relax budgetary constraints, leading to more diverse food consumption^18–20^. Previous studies also attribute more women working outside of the home to changes in food consumption. At the household level, women working outside of the home increases the value of time and puts convenience at a premium^7^. Finally, improved market access and associated changes in access to food also influence consumption diversity^21,22^. After controlling for household-level characteristics, we compared two multi-level models: (1) with urbanization (demographic share) level at the district-level and (2) with variables commonly associated with urbanization, namely infrastructure, market access, the proportion of working women in urban areas, and norms and institutions. Excluding household-level predictors and institutions (state-level), we measured all variables at the district level.

**References**

1. Zhang, Q., Pandey, B. & Seto, K. C. A Robust Method to Generate a Consistent Time Series From DMSP/OLS Nighttime Light Data. *IEEE Transactions on Geoscience and Remote Sensing* **54**, 5821–5831 (2016).

2. Nelson, A. Estimated travel time to the nearest city of 50,000 or more people in year 2000. *Ispra, Italy* (2008).

3. Davison, A. C. & Hinkley, D. V. *Bootstrap methods and their application*. vol. 1 (Cambridge university press, 1997).

4. Remans, R., Wood, S. A., Saha, N., Anderman, T. L. & DeFries, R. S. Measuring nutritional diversity of national food supplies. *Global Food Security* **3**, 174–182 (2014).

5. Chaudhary, A., Gustafson, D. & Mathys, A. Multi-indicator sustainability assessment of global food systems. *Nature communications* **9**, 1–13 (2018).

6. Sharma, A. & Chandrasekhar, S. Impact of commuting by workers on household dietary diversity in rural India. *Food Policy* **59**, 34–43 (2016).

7. Cockx, L., Colen, L. & De Weerdt, J. From corn to popcorn? Urbanization and dietary change: Evidence from rural-urban migrants in Tanzania. *World Development* **110**, 140–159 (2018).

8. Liu, J., Shively, G. E. & Binkley, J. K. Access to variety contributes to dietary diversity in China. *Food Policy* **49**, 323–331 (2014).

9. Popkin, B. M. Urbanization, Lifestyle Changes and the Nutrition Transition. *World Development* **27**, 1905–1916 (1999).

10. Thiele, S. & Weiss, C. Consumer demand for food diversity: evidence for Germany. *Food Policy* **28**, 99–115 (2003).

11. Finch, W. H., Bolin, J. E. & Kelley, K. *Multilevel modeling using R*. (Crc Press, 2016).

12. Bren d’Amour, C. *et al.* Urbanization, processed foods, and eating out in India. *Global Food Security* **25**, 100361 (2020).

13. Ikudayisi, A., Okoruwa, V. & Omonona, B. From the lens of food accessibility and dietary quality: Gaining insights from urban food security in Nigeria. *OUTLOOK ON AGRICULTURE* doi:10.1177/0030727019866462.

14. Reardon, T. & Timmer, C. P. Five inter-linked transformations in the Asian agrifood economy: Food security implications. *Global Food Security* **3**, 108–117 (2014).

15. Pingali, P. Westernization of Asian diets and the transformation of food systems: Implications for research and policy. *Food Policy* **32**, 281–298 (2007).

16. Keys, E. & McConnell, W. J. Global change and the intensification of agriculture in the tropics. *Global Environmental Change* **15**, 320–337 (2005).

17. Reardon, T. *et al.* Urbanization, diet change, and transformation of food supply chains in Asia. *Michigan: Global Center for Food Systems Innovation* (2014).

18. Abdulai, A., Jain, D. K. & Sharma, A. K. Household Food Demand Analysis in India. *Journal of Agricultural Economics* **50**, 316–327 (1999).

19. Deaton, A. & Paxson, C. Economies of Scale, Household Size, and the Demand for Food. *Journal of Political Economy* **106**, 897–930 (1998).

20. West, D. A. & Price, D. W. The Effects of Income, Assets, Food Programs, and Household Size on Food Consumption. *American Journal of Agricultural Economics* **58**, 725–730 (1976).

21. Liao, C. *et al.* City Level of Income and Urbanization and Availability of Food Stores and Food Service Places in China. *PLOS ONE* **11**, e0148745 (2016).

22. Schiff, N. Cities and product variety: evidence from restaurants. *J Econ Geogr* **15**, 1085–1123 (2015).
